# Nonsense-mediated mRNA decay safeguards telomeres in pluripotent stem cells

**DOI:** 10.1038/s41556-026-01912-0

**Published:** 2026-03-24

**Authors:** Marta Markiewicz-Potoczny, Si Young Lee, Soniya Chatterjee, Justin W. Mabin, Anna Zinsser, Ranjodh Sandhu, Gianna Tricola, J. Robert Hogg, Eros Lazzerini Denchi

**Affiliations:** 1 Department of Biochemistry and Molecular Genetics, University of Illinois, Chicago, IL USA; 2 National Institutes of Health, National Cancer Institute, Bethesda, MD USA; 3 Children’s National Research and Innovation Campus, Washington, DC USA; 4 National Institutes of Health, National Heart, Lung, and Blood Institute, Bethesda, MD USA; 5 Johns Hopkins School of Medicine, Johns Hopkins University, Baltimore, MD USA

**Keywords:** Telomeres, RNA decay

## Abstract

Telomeres are protective DNA caps at chromosome ends that prevent cells from mistakenly recognizing them as broken DNA. These structures are safeguarded by a protein complex called Shelterin, particularly through the TRF2 protein encoded by Trf2. Surprisingly, in mouse embryonic stem cells, TRF2 is not essential for telomere protection, suggesting that other mechanisms compensate for its loss. Here we show that a cellular quality control system called nonsense-mediated mRNA decay (NMD), which normally eliminates defective RNA molecules, plays an unexpected role in maintaining telomere integrity in pluripotent cells. Through a genome-wide genetic screen, we discovered that NMD is essential for cell survival when TRF2 is absent. NMD accomplishes this by degrading an aberrant form of the messenger RNA encoded by Trf1, which produces the TRF1 protein, another Shelterin component. Without NMD, this aberrant RNA produces a truncated, harmful version of TRF1 that interferes with normal telomere protection. Our findings reveal that embryonic stem cells use a unique strategy for chromosome end protection, linking RNA quality control to genome stability in a previously unrecognized way.

## Abstract (web summary)

Markiewicz-Potoczny et al. report that the nonsense-mediated mRNA decay pathway protects telomeres in the absence of core Shelterin component TRF2 by regulating the stability and abundance of TRF1 at telomeres.

## Main

Telomeres are nucleoprotein structures that cap the ends of linear chromosomes, protecting them from being recognized as DNA double-strand breaks^1–4^. In mammalian cells, telomeres consist of tandem repeats of the TTAGGG sequence bound by the Shelterin complex, which is essential for maintaining telomere integrity^1,4–7^. Within Shelterin, *Trf2* (telomeric repeat-binding factor 2) plays a central role in suppressing non-homologous end joining (NHEJ)-mediated chromosome fusions^1,8^. In somatic cells, loss of TRF2 triggers rapid telomere dysfunction, NHEJ activation at every chromosome end, and genomic instability^1,7,9^. However, mouse embryonic stem (mES) cells exhibit a unique tolerance to TRF2 depletion^10,11^. In these cells, *Trf2* deletion does not trigger a strong DNA damage response, NHEJ is not activated, and cells remain viable and can be propagated indefinitely, pointing to fundamental differences in telomere protection mechanisms between pluripotent and differentiated cells.

Although telomere protection has been mainly attributed to the Shelterin complex, emerging evidence indicates that RNA metabolism also contributes to genome stability, including at repetitive and telomere-proximal regions^12–16^. Several RNA surveillance pathways have been implicated in modulating telomeric RNA species and preventing the accumulation of aberrant RNA–DNA structures at chromosome ends, suggesting that post-transcriptional control influences telomere function^16–20^.

The nonsense-mediated mRNA decay (NMD) pathway is a conserved RNA surveillance mechanism that degrades transcripts harbouring premature termination codons (PTCs)^21,22^. Beyond quality control, NMD fine-tunes normal gene expression, influences lineage decisions, shapes neuronal and immune functions, and buffers cells against genotoxic stress contributing to broader post-transcriptional gene regulatory networks that influence cellular identity and genome stability^15,16,23–27^. The central effector of the pathway is UPF1 (Up-frameshift protein 1), an RNA helicase that serves as a platform for RNA decay complex assembly and activation in response to translation termination^21,28^. UPF1 activation requires phosphorylation, a process mediated by the SMG1 (Suppressors with morphogenetic defects in genitalia 1) kinase^29–32^. Once phosphorylated, UPF1 recruits additional downstream effectors: SMG5, SMG6 and SMG7^33,34^. SMG6 exerts endonucleolytic cleavage activity on the target mRNA, while the SMG5–SMG7 complex promotes both SMG6 activity and exonucleolytic degradation through the recruitment of cellular exonucleases^35–37^. UPF1 has been shown to interact with telomere proteins, promote telomere replication and facilitate the removal of RNA–DNA hybrids (R-loops) at telomeres, contributing to genome integrity. These findings suggest that the NMD pathway may have a critical role at telomeres that extends beyond its canonical function in RNA surveillance^16,17,19,20,38^.

Here, we show that the NMD pathway tightly controls the expression of the telomere-associated gene *Trf1*. A clustered regularly interspaced short palindromic repeats (CRISPR)–Cas9 synthetic lethality screen revealed that NMD factors are essential for cell viability in the absence of TRF2. We found that the loss of TRF2 in NMD-deficient cells leads to the accumulation of DNA damage factors at telomeres, frequent formation of end-to-end chromosomal fusions and loss of cell viability. Mechanistically, NMD-depleted cells accumulate a truncated, dominant-negative *Trf1* transcript in which exon 8 is skipped (TRF1^ΔE8^), leading to the production of a truncated, dominant-negative TRF1^ΔE8^ protein. TRF1^ΔE8^ lacks the DNA-binding MYB domain, yet retains the dimerization domain, therefore enabling displacement of the full-length TRF1 (TRF1^FL^) from telomeres. In our model, NMD deficiency lowers TRF1 availability at telomeres and, when combined with TRF2 loss, results in telomere dysfunction and cell death. Together, our finding uncovers a previously unrecognized post-transcriptional mechanism of telomere protection in mES cells.

## Results

### The NMD pathway is synthetically lethal with TRF2 in mES cells

**Fig. 1:**
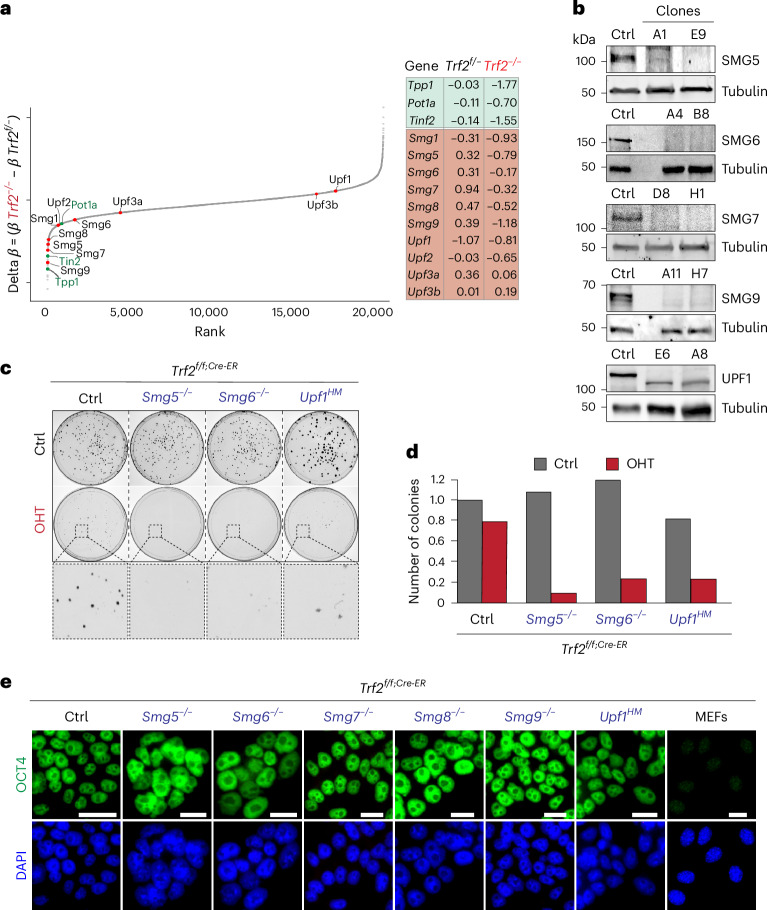
Loss of NMD leads to synthetic lethality in Trf2-deficient ES cells. **a**, A genome-wide CRISPR–Cas9 screen was performed on *Trf2*^−/−^ *and Trf2*^*f*/−^ ES cells. A rank-ordered plot shows the distribution of the difference between *β* scores (*y* axis, Delta *β* = *β* *Trf2*^−/−^ − *β* *Trf2*^*f*/−^) for all the genes targeted (about 23,000) (gene rank position, *x* axis). Genes previously been reported as synthetically lethal with *Trf2* are labelled in green, and the ones identified in this study are labelled in pink. **b**, Western blot showing efficient depletion of SMG5, SMG6, SMG7, SMG9 and UPF1. For each gene, two independent knockout clones as well as a non-edited control (Ctrl) are shown. Note that gene editing for *Upf1* results in a generation of a truncated protein leading to a hypomorph (HM) allele. Representative images from minimum two independent experiments per genotype are shown. **c**, Clonogenic survival assay was performed on cells of the indicated genotypes. OHT treatment induced TRF2 depletion. Cells were stained using crystal violet to count number of colonies (*n* = 2 biological replicates). **d**, Quantification of colonies shown in **c**: the number of colonies for each genotype was normalized to the number of colonies in the control (Ctrl) sample. For the TRF2-depleted samples (OHT) a magnification of a randomly chosen portion of the plate is shown. **e**, IF staining for the pluripotency marker OCT4 in ES cells of indicated genotypes, and mouse embryonic fibroblasts (MEFs), as a negative control. Representative images of three independently performed experiments are shown. Unprocessed blots and images are available in the Source data. Source data

To identify genes required for telomere protection in the absence of TRF2, we performed a genome-wide CRISPR–Cas9 synthetic lethal screen in *Trf2*-deficient (*Trf2*^−/−^) and *Trf2*-proficient (*Trf2*^*f*/−^) mES cells (Fig. 1a). We identified guide RNAs (gRNAs) that were selectively depleted in *Trf2*^−/−^ cells (*β* score <−0.75) but had little or no effect in controls (*β* score >0). This approach recovered known telomere-associated factors, including *Tpp1* and *Pot1a*, previously shown to be essential for proliferation in the absence of TRF2^10,11^. In addition, we identified five components of the NMD pathway (Fig. 1a and Supplementary Table 1).

**Extended Data Fig. 1:**
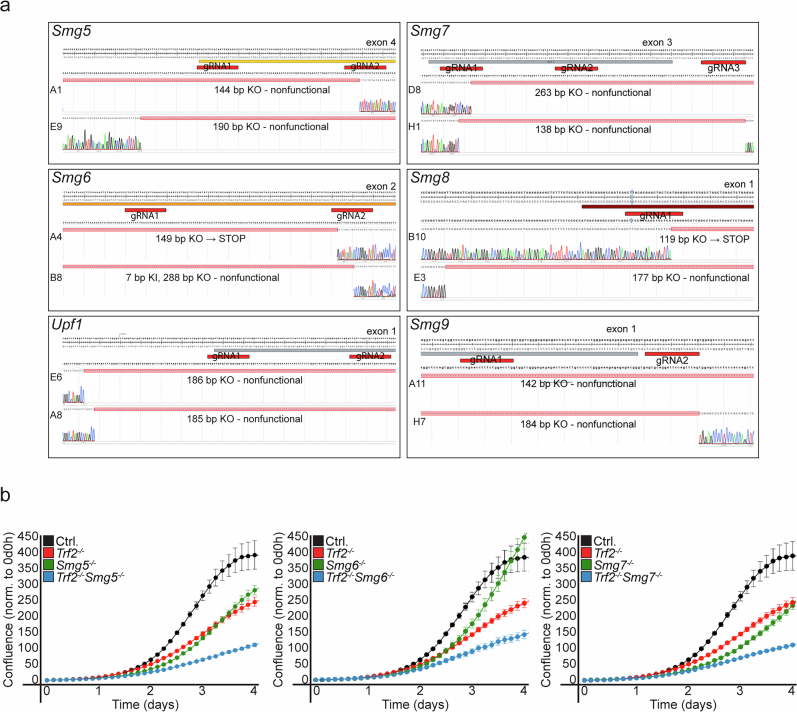
Generation and validation of NMD knockout ESC lines. **a**, Schematic of *Smg5, Smg6, Smg7, Smg8, Smg9* and *Upf1* loci. Sanger sequencing confirms gene editing in two clones per gene. gRNAs targeting regions are indicated in red. **b**, Growth curves of ESCs of indicated genotypes monitored by Incucyte S3 following OHT-mediated TRF2 deletion (day 0). Data are mean ± s.d. from 49 images per condition. Colors: Ctrl (black), *Trf2*^−/−^ (red), *Smg* knockouts (green), double mutants (blue).

**Extended Data Fig. 2:**
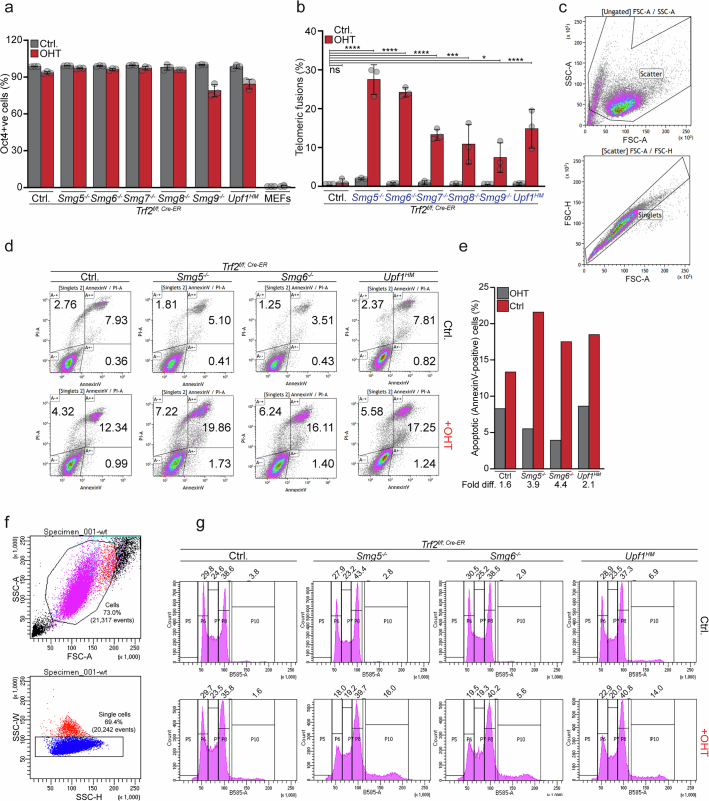
Increased end-to-end fusions in NMD-deficient *Trf2*^*−/−*^ ESCs. **a**, Quantification of OCT4+ve cells in indicated genotypes; MEFs serve as negative control. Related to Fig. 1e. Data from three independent experiments. More than 656 cells per genotype (n = 3 biological replicates) were scored. Details on the exact cell number per genotype are in Source Data File. **b**, Percentage of telomere fusions calculated from a total of >19 metaphases per genotype. More than 1981 chromosomes per genotype were scored. Details on the exact chromosome number per genotype are in Source Data File. Related to Fig. 2e. Statistical analysis (n = 3 biological replicates) by one-way ANOVA; *P = 0.0117 ns, not significant, P > 0.9999; ****P = 0.0001; ***P = 0.001; *P = 0.0124. **c**, Representative gating strategy for all genotypes in (**d**) is shown. **d**, Annexin-V and Propidium Iodide co-staining analysis by flow cytometry is shown for TRF2-proficient and TRF2-deficient cells in control (Ctrl) and NMD-deficient cells (Smg5^−/−^, Smg6^−/−^ and Upf1^HM^). Representative strategy from minimum three independent experiments per genotype are shown. **e**, Percentage of apoptotic cells positively stained with Annexin-V is shown. **f**, Representative gating strategy for all genotypes in (**g**) is shown**. g**, Cell cycle analysis by Propidium Iodide incorporation in fixed cells of indicated genotypes. Representative strategy from minimum three independent experiments per genotype are shown.

To validate this result, we generated knockout clones for the NMD factors *Smg5-9* as well as *Upf1* in *Trf2*^*Flox/Cre-ER*^ mES cells, using different gRNAs from those in the initial screen. We confirmed complete depletion of SMG5–9 (Fig. 1b–d and Extended Data Fig. 1a,b), as well as gene editing for *Upf1*, which produced a truncated protein that we consider a hypomorphic allele (*Upf1*^*HM*^) (Fig. 1b and Extended Data Fig. 1a). Loss of individual NMD components had no effect on ES cell proliferation, in line with what was reported for *Smg5*-, *Smg6*- and *Smg7*-deficient ES cells^23,24^ (Fig. 1c,d and Extended Data Fig. 1b). Next, we tested whether depletion of TRF2 in NMD-deficient cells resulted in loss of cell viability. Colony formation and cell proliferation assays (Fig. 1c,d and Extended Data Fig. 1b) showed that codepletion of TRF2 and NMD factors results in strong growth suppression, suggesting a synthetic lethal interaction. The reduced level of proliferation was associated with increased levels of apoptosis (Extended Data Fig. 2c–e), as well as an impaired cell cycle progression of cells codepleted for TRF2 and NMD factors (Extended Data Fig. 2f,g). Importantly, all the knockouts analysed retained OCT4 expression (Fig. 1e and Extended Data Fig. 2a), indicating pluripotency maintenance and lack of differentiation.

### *Trf2*^−/−^ NMD-deficient ES cells exhibit telomeric DNA damage and frequent end-to-end fusions

**Fig. 2:**
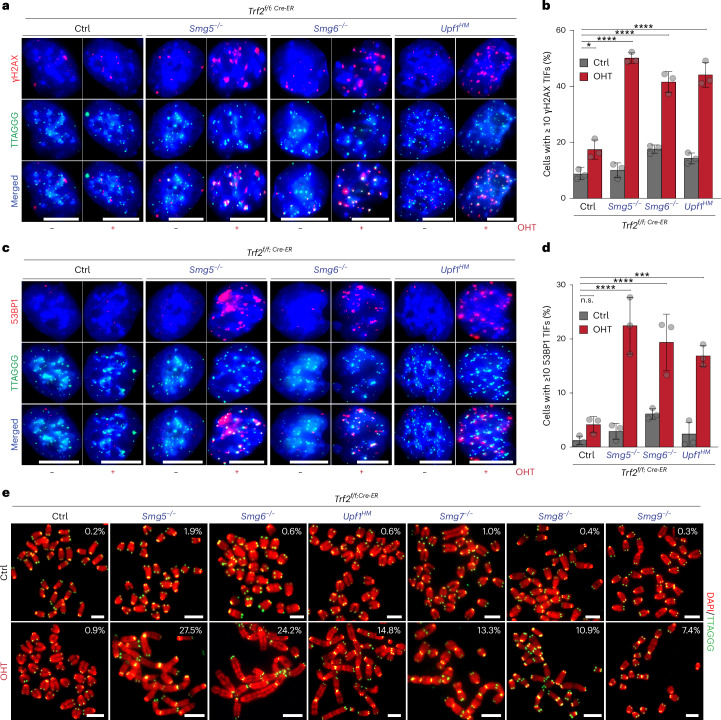
NMD is required for telomere protection in the absence of TRF2. **a**, Representative IF–FISH showing γH2AX (red) and telomeric DNA (green) in NMD-proficient cells (Ctrl) and NMD-deficient ES cells with or without OHT treatment to induce TRF2 depletion. **b**, Quantification of telomere dysfunction-induced foci (TIFs) in **a**, defined as ≥10 γH2AX foci colocalizing with telomeres. Data are mean ± s.d. from three biological replicates. Statistical analysis (*n* = 3 biological replicates) by one-way ANOVA; *P = 0.0117, ****P = 0.0001. **c**, Representative IF–FISH images showing 53BP1 (red) and telomeric DNA (green) in the indicated genotypes ± OHT. **d**, Quantification of TIFs as in **b** using 53BP1 foci colocalization. Data are mean ± s.d. from three biological replicates. Statistical analysis (*n* = 3 biological replicates) by one-way ANOVA; n.s., not significant (*P* = 0.9153), ***P = 0.0002, ****P = 0.0001. In **b**–**d**, more than 551 cells in total per genotype were scored. For details on the exact cell number per genotype, see the Source data. **e**, Representative metaphases from ES cells with indicated genotypes ± OHT. Telomeric fusions per metaphase are indicated within each panel. More than 1,981 chromosomes in total per genotype were scored (*n* = 3 biological replicates). For details on the exact chromosome number per genotype, see the  Source data. For additional data, see Extended Data Fig. 2b. Source numerical data, unprocessed blots and images are available in the Source data. Source data

To determine whether the synthetic lethality between *Trf2* and NMD is driven by telomere-specific defects, we asked whether telomeres are recognized as sites of DNA damage in ES cells lacking both TRF2 and NMD factors. Our data show that individual depletion of TRF2 or NMD factors did not result in the accumulation of DNA damage markers γH2AX (Fig. 2a,b) and 53BP1 (Fig. 2c,d) at telomeres. However, codepletion of TRF2 and NMD components resulted in a marked increase in the localization of both DNA damage markers at telomeres (Fig. 2a–d). These data suggest that NMD activity contributes to telomere protection in TRF2-depleted cells.

Next, we assessed whether TRF2-depleted telomeres, in the absence of NMD activity, undergo end-to-end chromosome fusions. Metaphase analysis confirmed that the deletion of *Trf2* is not sufficient to trigger telomere fusions (Fig. 2e) and revealed that NMD single knockouts show no increase in fusions relative to the parental cell line (Fig. 2e and Extended Data Fig. 2b). By contrast, TRF2 depletion in the context of an NMD-deficient background resulted in frequent fusions (Fig. 2e and Extended Data Fig. 2b). The frequency of telomeric fusions varied among the different NMD-deficient backgrounds. *Smg5*^−/−^ and *Smg6*^−/−^ showed the highest fusion rates (27.5% and 24.2%), followed by *Upf1*^*HM*^ and *Smg7*^−/−^ (14.8% and 13.3%), while *Smg8*^−/−^ and *Smg9*^−/−^ exhibited lower levels (10.9% and 7.4%) (Fig. 2e and Extended Data Fig. 2b). Despite these differences, all the *Trf2*^−/−^ *NMD*^−/−^ cell lines analysed showed frequent telomere fusions and failed to proliferate (Figs. 1c and 2e and Extended Data Figs. 1b and 2b). Thus, the observed DNA damage activation at telomeres and frequent chromosome fusions provide a mechanistic basis for the synthetic lethality between *Trf2* loss and impaired NMD in ES cells.

### Acute NMD inhibition in ES cells results in telomeric fusions

**Fig. 3:**
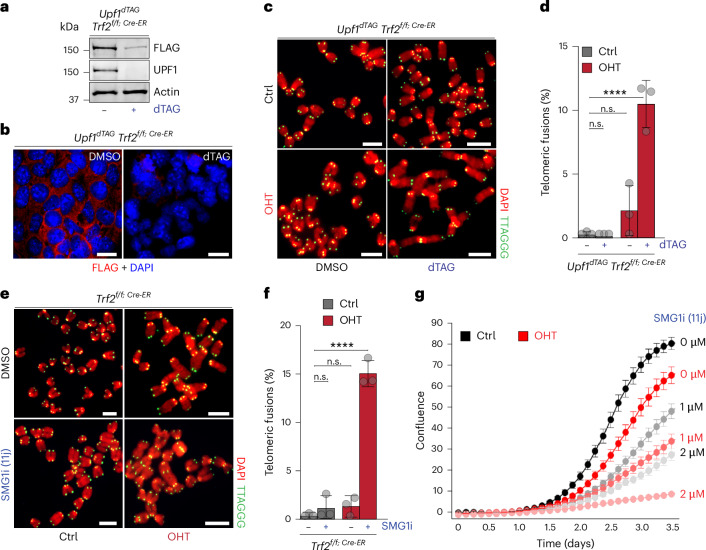
Effects of acute NMD depletion on viability and telomere integrity in *Trf2*^−/−^ ES cells. **a**, Western blot showing expression of UPF1 in *Upf1*^*dTAG*^ ES cells either treated with dimethyl sulfoxide (DMSO) (−) or treated with the small molecule dTAG-13 (+). Detection performed by antibody detecting endogenous UPF1, or by anti-FLAG antibody detecting *Upf1*^*dTAG-FLAG*^. Representative images from minimum two independent experiments per genotype are shown. **b**, FLAG IF in UPF1^dTAG^ treated with DMSO or dTAG-13 (dTAG). **c**, FISH for telomeric DNA (green) and DAPI staining (red) on metaphases derived from TRF2-proficient (Ctrl) or TRF2-deficient (OHT) ES cells, either left untreated (DMSO) or treated with dTAG-13 (dTAG) to degrade UPF1. **d**, Quantification of telomeric fusions shown in **c**, mean ± s.d. from three (*n* = 3) biological replicates. More than 1,656 chromosomes per genotype were scored. For details on the exact chromosome number per genotype, see the Source data. Statistical analysis by one-way ANOVA; n.s., not significant (*P* > 0.9999; *P* = 0.3962), ****P = 0.0001. **e**, Representative metaphases from ESCs treated with the SMG1 inhibitor 11j ± OHT. **f**, Quantification of telomeric fusions in **e**, mean ± s.d., *n* = 3 biological replicates. Statistical analysis by one-way ANOVA; n.s., not significant (*P* = 0.7548, *P* = 0.6116), ****P = 0.0001. More than 2,130 chromosomes per genotype were scored. For details on the exact chromosome number per genotype, see the Source data. **g**, Cell proliferation of TRF2-proficient (black lines) and TRF2-deficient (OHT-treated, red lines) cells in the presence of increasing concentrations of the SMG1 inhibitor (SMG1i). Cells were either untreated (0 μM) or treated with the indicated doses of SMG1i. Proliferation was monitored by confluence using the Incucyte S3 system. Data represent the mean ± s.d derived from the analysis of 49 images per condition. Representative images from minimum two independent experiments per genotype are shown. Source numerical data, unprocessed blots and images are available in Source data.

**Extended Data Fig. 3:**
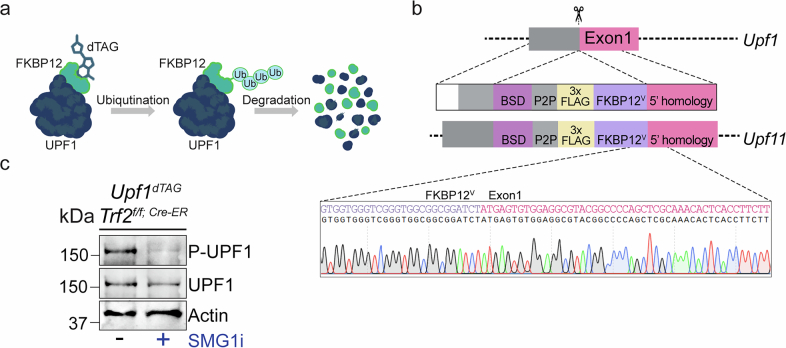
Generation of a degradable UPF1 mECS cell line. **a**, Schematic of the dTAG degron system. The dTAG ligand recruits UPF1-FKBP12F36V to the CRBN E3 ubiquitin ligase complex, triggering targeted protein degradation^1^. **b**, Schematic of the FLAG-dTAG knock-in strategy at the endogenous *Upf1* locus and validation of successful knock-in by Sanger sequencing. Chromatogram shows the integration site of the FLAG-FKBP12F36V within exon 1 of *Upf1* in a correctly targeted mESC clone. **c**, Western blot showing expression of P-UPF1 and UPF1 in UPF1^dTAG^ ESCs either treated with DMSO (-) or treated with the SMG1 inhibitor (SMG1i). Representative images from minimum three independent experiments are shown.

To rule out clonal selection artefacts, we acutely inhibited NMD using two approaches. To this end, we used two independent approaches. First, we introduced a TAG degron (dTAG)^39^ at the endogenous *Upf1* locus (Extended Data Fig. 3a,b) in *Trf2*^*Flox/Cre-ER*^ mES cells. Upon addition of the dTAG ligand, UPF1 is rapidly degraded as confirmed by western blot and immunofluorescence (IF) (Fig. 3a,b). In these cells, TRF2 was first depleted with OHT, then UPF1 was degraded through dTAG treatment for 48 h. Notably, in this setting, codepletion of TRF2 and UPF1 also resulted in frequent telomeric fusions (~12%) (Fig. 3c,d), comparable to those observed in constitutive knockout cell lines (Fig. 2e and Extended Data Fig. 2b). This is in striking contrast to the phenotype of cells depleted for either *Trf2* or *Upf1* alone (Fig. 2e and Extended Data Fig. 2b). This result indicates that acute disruption of NMD is sufficient to compromise telomere protection, excluding a confounding effect arising during clonal selection of NMD-deficient cells. As an alternative method, we pharmacologically inhibited the NMD pathway using the SMG1 inhibitor 11j (SMG1i), a pyrimidine analogue that blocks UPF1 phosphorylation^40^ (Extended Data Fig. 3c). We first deleted TRF2 through OHT treatment, followed by treatment with the NMD-inhibitor 11j (1 µM) for an additional 48 h. Similar to the data obtained by genetic ablation, SMG1i treatment in TRF2-depleted ES cells led to frequent telomeric fusions (~15%) (Fig. 3e,f). Consistent with these findings, we also observed a notable growth defect in cells treated with SMG1i (1 μM and 2 μM) in conjunction with OHT-mediated TRF2 deletion (Fig. 3g). Collectively, these data further corroborate the notion that NMD activity contributes to telomere protection in mES cells in the absence of TRF2.

### NMD deficiency results in accumulation of a dominant-negative TRF1 isoform (TRF1^ΔE8^) by preventing its degradation

**Fig. 4:**
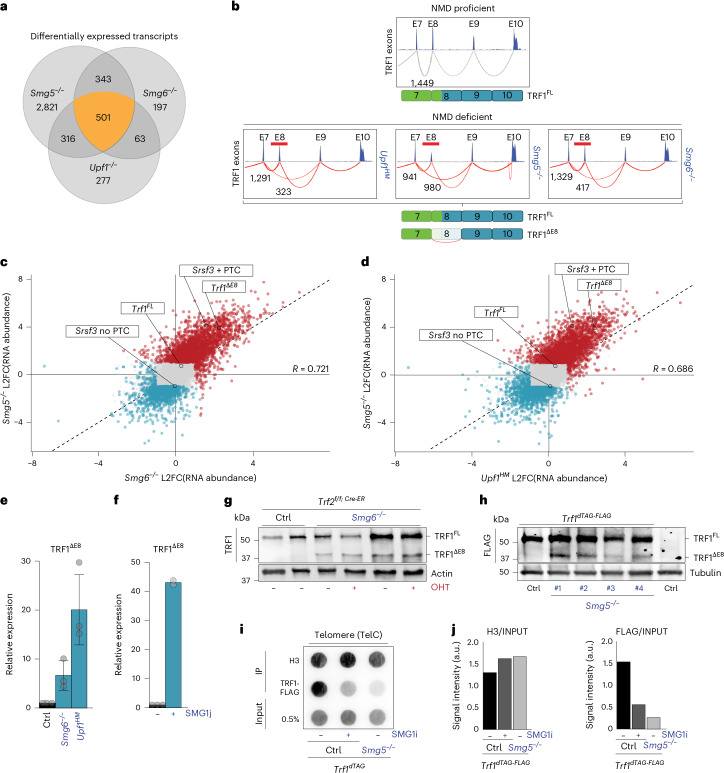
NMD loss leads to expression of a dominant-negative TRF1^ΔE8^ isoform. **a**, Venn diagram illustrating the overlap and unique sets of differentially expressed transcript isoforms in *Smg5*^−/−^, *Smg6*^−/−^ and *Upf1*^*HM*^ ES cells compared with NMD-proficient controls. Numbers indicate the total number of isoforms that are differentially expressed either uniquely in one genotype or shared across two or more conditions. **b**, Sashimi plots showing splicing events across exons 7–10 of the *Trf1* transcript in wild type (NMD-proficient) and *Upf1*^*HM*^, *Smg6*^−/−^ *and Smg5*^−/−^ ES cells. Junction read numbers are shown. Note the presence of a splice variant lacking exon 8 (*Trf1*^*ΔE8*^) in NMD-deficient ES cells. **c**, Scatter plot showing differential RNA abundance (L2FC) in *Smg5*^−/−^ (*y* axis) and *Smg6*^−/−^ (*x* axis) ES cells relative to wild-type controls. Each point represents a transcript isoform. Isoforms significantly upregulated are shown in red, downregulated in blue and non-significantly changed in grey. NMD-sensitive (PTC-containing) and NMD-insensitive isoforms of *Trf1* and *Srsf3* are labelled. The Pearson correlation coefficient (*R*), indicating the similarity in isoform expression changes between the two genotypes, is shown on the right. **d**, The same as **c**, but comparing *Smg5*^−/−^ (*y* axis) and *Upf1*^*HM*^ (*x* axis) ES cells. **e**, Detection of the splice variant lacking exon 8 (*Trf1*^*ΔE8*^) by RT–qPCR in NMD-proficient (Ctrl), *Smg6*^−/−^ and *Upf1*^*HM*^ ES cells. Data are mean ± s.d. from three biological replicates; statistical analysis by one-way ANOVA. **f**, Detection of the splice variant lacking exon 8 (*Trf1*^*ΔE8*^) by RT–qPCR in cells either left untreated or treated with the SMG1 inhibitor, 11j. Two biologically independent SMG1 inhibitory experiments were performed; data points are shown on the graph. **g**, Western blot analysis for TRF1 expression in NMD-proficient (Ctrl) cells and *Smg6*^−/−^ ES cells. Actin was used as a loading control. **h**, Western blot analysis for the expression of the TRF1–dTAG–FLAG fusion protein in cells in which the endogenous TRF1 gene was either untagged (WT) or tagged with a dTAG–FLAG construct (TRF1^dTAG^). Cell lysates were collected from SMG5-proficient cells (Ctrl). For **g** and **h**, representative images from three independent experiments per genotype are shown. **i**, ChIP assay performed on TRF1^dTAG^ ES cells that are SMG5-proficient (Ctrl) or deficient (*Smg5*^−/−^), in the presence (+) of, or in the absence of the SMG1 inhibitor 11j. Input DNA (0.5% of the total DNA used), as well as DNA pulled down with H3 or FLAG, was hybridized with a radio-labelled telomeric probe (TelC). **j**, ChIP and input signals from **i** were quantified using ImageJ, and the ChIP signal was normalized with the corresponding input signal for both H3 and FLAG. Source numerical data and unprocessed blots are available in the Source data.

**Extended Data Fig. 4:**
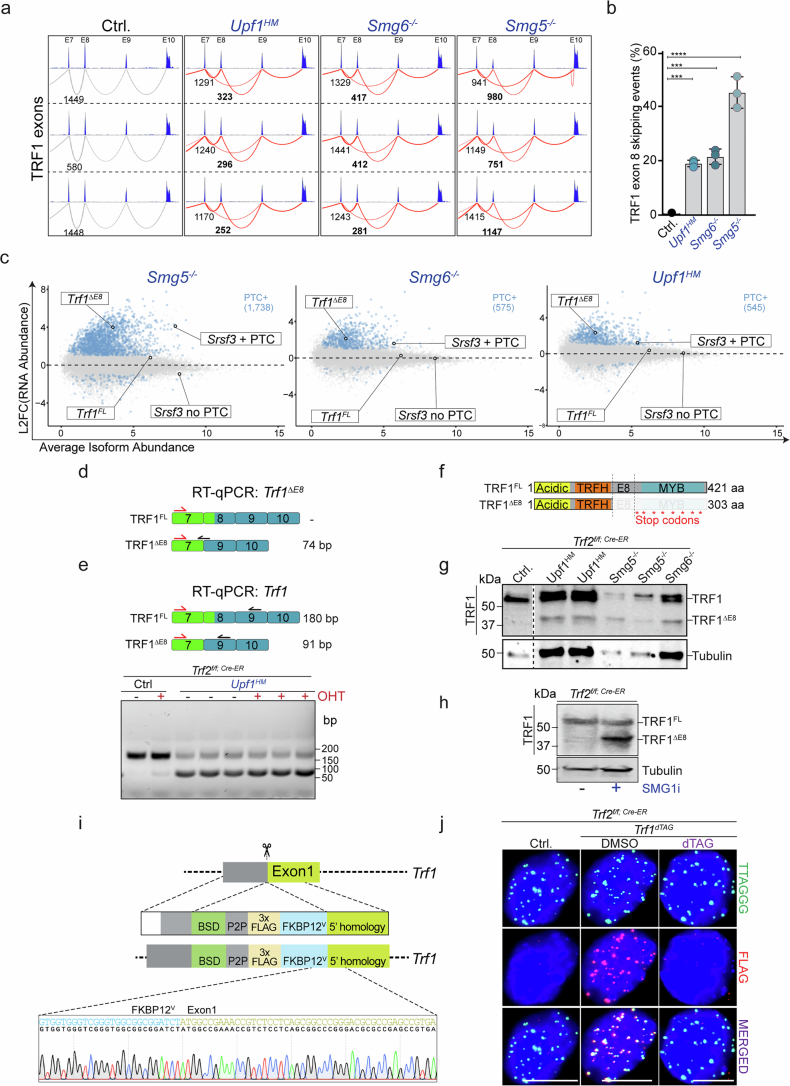
Characterization of TRF1 exon 8 skipping events in ESCs. **a**, Sashimi plots of TRF1 splicing across exons 7–10 in Ctrl and NMD-deficient ESCs. Triplicates (n = 3) and junction read numbers are shown. Top panel as in Fig. 4b. **b**, Graph showing quantification of exon 8 skipping across genotypes as percentage of total splicing events. Statistical analysis (n = 3 biological replicates) by one-way ANOVA; ***P = 0.0004, ***P = 0.0002; ****P = 0.00001. **c**, Scatter plots showing the relationship between differential RNA isoform abundance (log_2_ fold change, y axis) and average isoform expression (x axis) in *Smg5*^−/−^ (left), *Smg6*^−/−^ (middle), and *Upf1*^HM^ (right) ESCs relative to wild-type cells. Each point represents a transcript isoform. Significantly upregulated isoforms are highlighted in red, significantly downregulated in blue, and nonsignificant changes are shown in gray. NMD-sensitive (PTC-containing) and NMD-insensitive isoforms of *Trf1* and *Srsf3* are labeled. **d**, Schematic of the RT-PCR strategy used in Fig. 4e and Fig. 4f to detect the splice variant lacking exon 8 (TRF1^DE8^) using a forward primer on Exon 7 and a reverse primer spanning the exon 7 - exon 9 junction. **e**, A RT-PCR strategy used to detect TRF1^FL^ and TRF1^ΔE8^. Representative agarose gel showing the amplification products in NMD-proficient (Ctrl) and NMD-deficient (*Upf1*^*HM*^) cells. **f**, Schematic of TRF1^FL^ and TRF1^ΔE8^ isoforms. Skipping exon 8 causes frameshift and premature stop codon in exon 9. **g**, Western blot analysis using an antibody against TRF1 (top panel) and Tubulin as a loading control (bottom panel) on total cell lysates from ESCs of the indicated genotypes. Bands corresponding to full-length TRF1 (TRF1^FL^) and the truncated isoform lacking exon 8 (TRF1^ΔE8^) are indicated. Representative images from minimum three independent experiments are shown. **h**, Western blot analysis using an antibody against TRF1 (top panel) and Tubulin as a loading control (bottom panel) on total cell lysates from control ESCs untreated (-) or treated (+) with SMG1 inhibitor (11j). Bands corresponding to full-length TRF1 (TRF1^FL^) and the truncated isoform lacking exon 8 (TRF1^ΔE8^) are indicated. Representative images from minimum three independent experiments are shown. **i**, Schematic of the FLAG-dTAG knock-in strategy at the endogenous *Trf1* locus. Successful integration was validated by Sanger sequencing. **j**, Immunofluorescence combined with FISH (IF-FISH) for telomeric DNA (TTAGGG, green) and FLAG (red) in control ESCs (Ctrl) and ESCs harboring the FLAG-dTAG construct at the *Trf1* locus (*Trf1*^*dTAG*^), either treated with DMSO or dTAG.

RNA sequencing (RNA-seq) analysis of *Smg5*^−/−^*, Smg6*^−/−^ and *Upf1*^*HM*^ cell lines identified genes deregulated by NMD loss. Differential isoform expression analysis in *Smg5*^−/−^*, Smg6*^−/−^ and *Upf1*^*HM*^ revealed that loss of SMG5 had the most pronounced effect compared with the depletion of other NMD factors (Fig. 4a), consistent with previous reports^23,37,41^. This analysis identified 501 transcript isoforms that were differentially expressed across all three genotypes (Fig. 4a and Supplementary Table 2). The depletion of NMD factors led to both upregulation and downregulation of RNA isoforms, including well-established NMD targets such as *Srsf3* (Fig. 4c,d and Extended Data Fig. 4c). Notably, among the genes significantly affected upon NMD inhibition was *Trf1*, which encodes the telomere-associated protein TRF1 (Fig. 4b–d and Extended Data Fig. 4a–c). Transcriptome analysis revealed that NMD-deficient cell lines accumulate a splice variant of *Trf1* lacking exon 8 (*Trf1*^*ΔE8*^) (Fig. 4b and Extended Data Fig. 4a). This isoform is predicted to be a NMD target owing to the presence of PTCs in exon 9 (Extended Data Fig. 4f). Based on isoform abundances in our RNA-seq dataset, we estimate that TRF1^ΔE8^ isoform accounts for approximately 20–50% of total *Trf1* transcripts in NMD-deficient cells (Extended Data Fig. 4a–c). Transcripts upregulated in NMD-deficient cells were highly enriched for PTC-containing isoforms, including *Trf1*^*ΔE8*^ and PTC containing *Srsf3* isoforms, indicating that the majority of the observed expression changes were due to inhibition of NMD activity^42,43^ (Fig. 4c,d and Extended Data Fig. 4c).

To confirm this observation, we designed a reverse transcription quantitative polymerase chain reaction (RT–qPCR) strategy to amplify selectively the *Trf1*^*ΔE8*^ splice variant targeting the exon 7–9 junction (Extended Data Fig. 4d,e). Using this approach, we confirmed that *Smg6*-, *Upf1*- and *Smg5*-depleted cell lines display elevated expression of the TRF1^ΔE8^ isoform (Fig. 4e and Extended Data Fig. 4d). Furthermore, amplification of *Trf1* transcripts using primers complementary to exon 7 and exon 9 revealed the presence of a smaller isoform corresponding to the size of *Trf1*^*ΔE8*^ in NMD-deficient cells (Extended Data Fig. 4e). Sequencing analysis confirmed that the smaller isoform corresponds to the *Trf1*^*ΔE8*^ splice variant. Finally, we tested whether acute inhibition of the NMD pathway results in accumulation of the *Trf1*^*ΔE8*^ isoform. For this experiment we treated wild-type cells with SMG1i, and we consistently observed a significant (>40-fold) accumulation of the *Trf1*^*ΔE8*^ isoform (Fig. 4f), supporting the notion that the expression of *Trf1* isoforms is tightly regulated by the NMD pathway.

Given the abundance of the *Trf1*^*ΔE8*^ isoform in NMD deficient cells, we next asked whether this isoform is translated into a protein. The predicted truncated protein expressed by *Trf1*^*ΔE8*^ retains the N-terminal acidic domain and the TRF dimerization domain (TRFH) but lacks the C-terminal DNA binding domain (Myb) (for schematics, see Extended Data Fig. 4f). Using an antibody raised against TRF1, we detected a truncated TRF1 protein in *Smg6*-deficient cells that was not present in wild-type control cells (Fig. 4g). The size of this protein is consistent with the predicted molecular weight (~34.2 kDa) of the *Trf1*^*ΔE8*^ translation product (Extended Data Fig. 4f). Similarly, *Smg5*-deficient and *Upf1*^*HM*^ cells (Extended Data Fig. 4g), as well as cells treated with SMG1i, showed accumulation of the truncated protein (Extended Data Fig. 4h).

Given that TRF1 binding to telomeres requires homodimerization, and that the truncated protein encoded by the *Trf1*^*ΔE8*^ isoform retains the TRFH dimerization domain (TRFH) but lacks the DNA binding Myb domain, we reasoned that this truncated protein could act as a ‘dominant negative’ by sequestering full-length TRF1 away from telomeres, similar to previously engineered dominant-negative *Trf1* alleles^44^. To test this hypothesis, we performed chromatin immunoprecipitation (ChIP) experiments to determine whether NMD-deficient cells expressing the *Trf1*^*ΔE8*^ have a reduced level of TRF1 bound to telomeres. To avoid confounding effects due to potential differences in antibody affinity between *Trf1* isoforms, we tagged the endogenous *Trf1* locus with a FLAG epitope using CRISPR-mediated gene editing. The resulting cells (see Extended Data Fig. 4i for schematics) expressed FLAG-tagged TRF1 that localized to telomeres (Extended Data Fig. 4j). Depletion of SMG5 in these cells led to the expected accumulation of a truncated TRF1 protein detectable with the FLAG antibody (Fig. 4h). Using these cells, we quantified the fraction of telomeric DNA associated with TRF1 by ChIP with an anti-FLAG antibody. This analysis revealed that TRF1 binding to telomeres was substantially reduced (~3-fold) in NMD-deficient cells compared with NMD-proficient control cells (Fig. 4i,j). Similarly, treatment with SMG1i also caused a marked reduction in TRF1 association with telomeres (Fig. 4i,j). Together, these results suggest that NMD activity regulates TRF1 localization to telomeres by controlling expression of the *Trf1*^*ΔE8*^ isoform.

### TRF1^ΔE8^ drives telomere dysfunction in TRF2-depleted ES cells

**Fig. 5:**
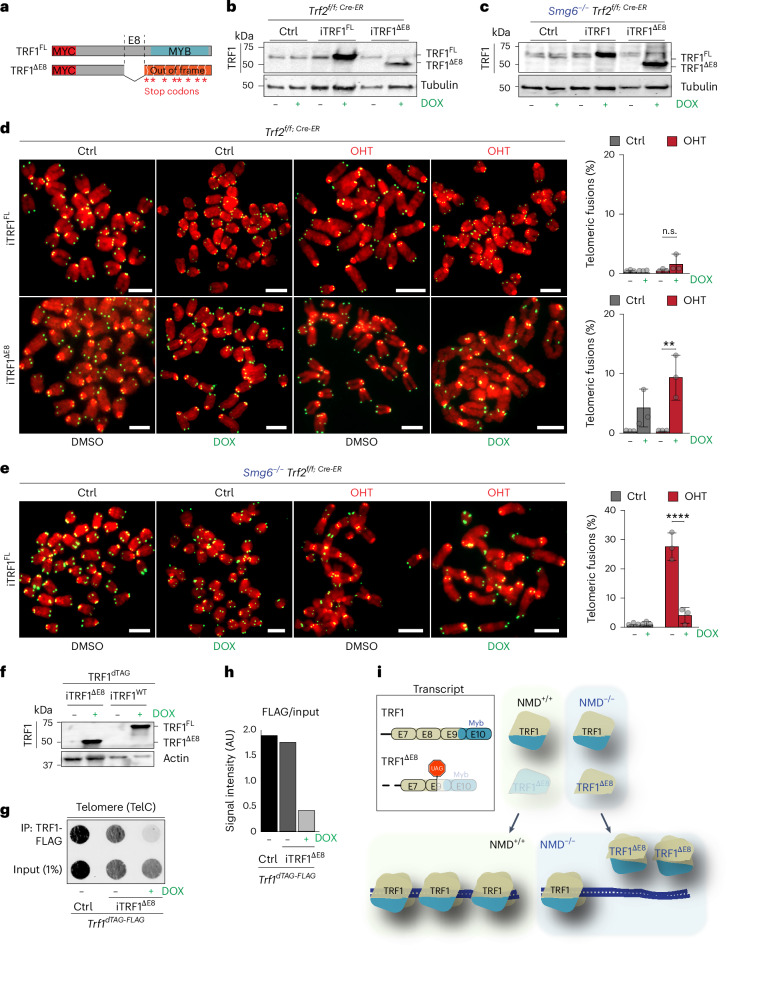
Elevated TRF1 levels prevent telomere fusions in NMD-deficient cells. **a**, Schematic of Myc-tagged TRF1^FL^ and TRF1^ΔE8^ DOX-inducible constructs. Stop codons resulting from exon 8 skipping events are indicated in the *Trf1*^*ΔE8*^ construct. **b**,**c**, Expression of TRF1 and, as a loading control, tubulin in NMD-proficient cells (**b**) and SMG6-deficient cells (**c**). DOX treatment results in the expression of full length TRF1 in iTRF1^FL^ cells and of the TRF1^ΔE8^ variant in the iTRF1^ΔE8^ cells. **d**, Metaphases derived from Ctrl or *Trf2*^−/−^ ES cells expressing TRF1^FL^ or TRF1^ΔE8^ ± DOX. Percentage of telomere fusions were calculated from a total of >17 metaphases per genotype. More than 678 chromosomes per genotype (*n* = 3 biological replicates) were scored. For details on the exact chromosome number per genotype, see the Source Data. Mean ± s.d., one-way ANOVA; n.s., not significant (*P* ≥ 0.9950), **P = 0.0013. **e**, Metaphases from *Smg6*^−/−^ or *Smg6*^−/−^ *Trf2*^−/−^ ES cells expressing TRF1^FL^ ± DOX. The percentage of telomere fusions was calculated from a total of >19 metaphases per genotype. More than 880 chromosomes per genotype (*n* = 3 biological replicates) were scored. For details on the exact chromosome number per genotype, see the Source Data. Mean ± s.d., one-way ANOVA; ****P = 0.0001. **f**, Expression of TRF1^FL^ and iTRF1^ΔE8^ without (−) and upon DOX treatment (+DOX), with actin as a loading control. **g**, ChIP assay performed on TRF1^dTAG^ ES cells (Ctrl.), untreated TRF1^dTAG^ iTRF1^ΔE8^ (−) and treated with DOX (+DOX). Input DNA (1% of the total DNA used), as well as DNA pulled down with FLAG, was hybridized with a radio-labelled telomeric probe (TelC). A representative image from three independent experiments per genotype is shown. **h**, ChIP and input signals from **g** were quantified using ImageJ, the ChIP signal was normalized with the corresponding input signal for FLAG. **i**, Model: NMD activity restrict the expression of the *Trf1*^*ΔE8*^ splice variant. Upon NMD inhibition, the *Trf1*^*ΔE8*^ transcript accumulates, producing TRF1^ΔE8^ protein, which binds full-length TRF1, and interferes with its binding to telomeres, resulting in telomere deprotection. Source numerical data, unprocessed blots and images are available in the Source data.

To test whether expression of *Trf1*^*ΔE8 i*^s sufficient to trigger telomere dysfunction and induce telomere fusions in TRF2-depleted mES cells, we generated cells in which either *Trf1*^*ΔE8*^ or full-length *Trf1* (*Trf1*^*FL*^) can be expressed in a doxycycline (DOX)-inducible manner (Fig. 5a–c). To assess the impact of these constructs on telomere integrity, we performed metaphase analysis. Overexpression of TRF1^FL^, in either the presence or absence of TRF2, did not induce telomere fusions (Fig. 5d). By contrast, DOX-mediated induction of TRF1^ΔE^ was sufficient to trigger frequent telomere fusions in TRF2-depleted cells, similar to what was observed in NMD-deficient cells (Fig. 5d). Notably, TRF1^ΔE8^ expression also led to telomere fusions in the presence of TRF2, suggesting that high levels of this truncated isoform are sufficient to disrupt telomere protection even when TRF2 is present. However, when TRF2 depletion was combined with TRF1^ΔE8^ expression, the frequency of telomere fusions reached levels comparable to those observed in NMD-deficient cells (Fig. 2e and Extended Data Fig. 2b), indicating that the *Trf1* C-terminal truncated protein probably acts in a dominant-negative manner.

### TRF1 overexpression is sufficient to restore NMD-mediated telomere protection

**Extended Data Fig. 5:**
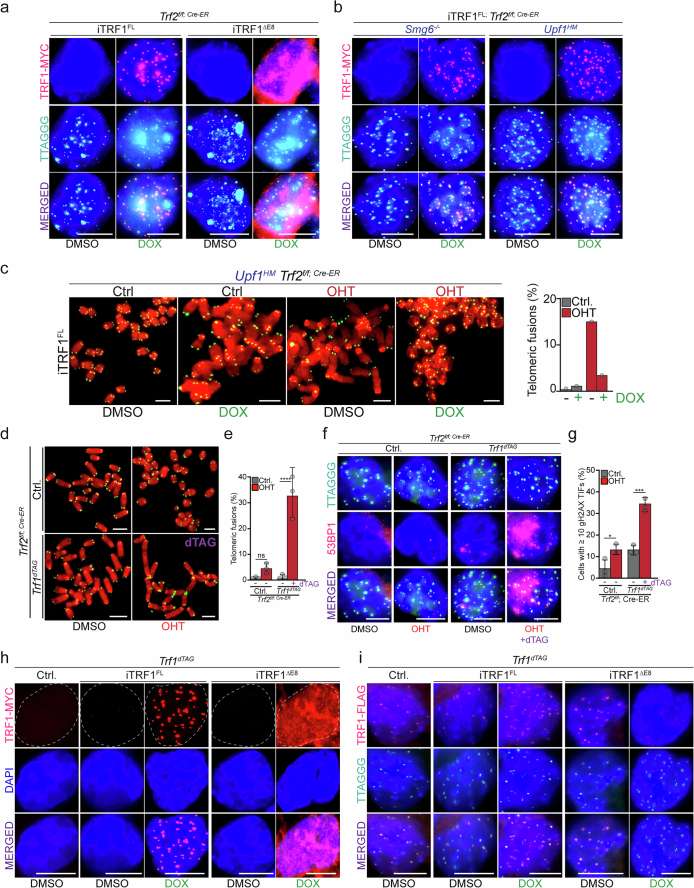
Localization of TRF1^FL^ and TRF1^ΔE8^. **a**, Immunofluorescence combined with FISH (IF-FISH) showing the expression and localization of Myc-tagged inducible TRF1 constructs (red) and telomeric DNA (TTAGGG, green) in cells with stable integration of either full-length TRF1 (iTRF1^FL^) or TRF1 lacking exon 8 (iTRF1^ΔE8^). Cells were treated with doxycycline (DOX) to induce construct expression or with DMSO as a control. More than 155 cells per genotype were scored. Details on the exact cell number per genotype are in Source Data File. **b**, IF-FISH for Myc (red) and telomeric DNA (TTAGGG, green) performed in *Smg6*^*−/−*^ or *Upf1*^*HM*^ ESCs stably expressing full-length inducible TRF1 (iTRF1^FL^), treated with either DMSO or doxycycline (DOX). More than 201 cells per genotype were scored. Details on the exact cell number per genotype are in Source Data File. **c**, Metaphase spreads from *Upf1*^*HM*^ and *Upf1*^*HM*^ *Trf2*^*−/−*^ ESCs either untreated (DMSO) or expressing ectopic full-length TRF1 (TRF1^FL^) (DOX). Quantification of telomeric fusions is shown. More than 248 chromosomes per genotype (n = 1 biological replicate) were scored. Details on the exact cell number per genotype are in Source Data File. **d**, Metaphase spreads from *Trf2*^*f/f*^ *and Trf2*^*f/f*^ *Trf2*^*dTAG*^ ESCs either untreated (DMSO) or treated with OHT and dTAG. Quantification of telomeric fusions is shown (**e**). More than 834 chromosomes per genotype (n = 3 biological replicates) were scored. Details on the exact cell number per genotype are in Source Data File. Statistical analysis by one-way ANOVA; ns, not significant, P = 0.6850, ****P = 0.00001. **f**, Representative Immunofluorescence-Fluorescence In Situ Hybridization (IF–FISH) showing gH2AX (red) and telomeric DNA (green) in *Trf2*^*f/f*^ (Ctrl.) and *Trf2*^*f/f*^ *Trf2*^*dTAG*^ ESCs with or without 4-hydroxytamoxifen (OHT) treatment to induce TRF2 depletion, and without or with dTAG treatment ( + dTAG) to induce TRF1 depletion. **g**, Quantification of telomere dysfunction-induced foci (TIFs) in (**f**), defined as ≥10 gH2AX foci colocalizing with telomeres. Data are mean ± s.d. from three (n = 3) biological replicates. Statistical analysis by one-way ANOVA; *P = 0.0342; ***P = 0.001. More than 155 cells per genotype were scored. Details on the exact cell number per genotype are in Source Data File. **h**, Immunofluorescence for Myc (red) in control *Trf1*^*dTAG*^ ESCs (Ctrl.), or *Trf1*^*dTAG*^ cells stably expressing full-length inducible TRF1 (iTRF1^FL^) or truncated TRF1 (iTRF1^DE8^), treated with either DMSO or doxycycline (DOX). **i**, IF-FISH for FLAG (red) and telomeric DNA (TTAGGG, green) performed in cells of indicated genotypes and under conditions as in (**h**).

The data outlined above suggest that accumulation of TRF1^ΔE8^ is sufficient to phenocopy the telomere deprotection phenotype seen in TRF2/NMD codepleted cells. To directly test whether reduced levels of TRF1 at telomeres are the primary cause for the loss of telomere dysfunction in this context, we asked whether increasing the levels of TRF1^FL^ in NMD-deficient cells would be sufficient to suppress the accumulation of telomere fusions upon TRF2 depletion. To this end, we introduced a DOX-inducible TRF1^FL^ expression construct in *Smg6*-deficient, TRF2 conditional mES cells. In this genetic background, as expected, TRF2 depletion triggers frequent telomere fusions (Fig. 5e). Notably, TRF1 expression was sufficient to almost completely suppress telomere fusions, reducing their frequency from ~30% to 5% (Fig. 5e). A similar suppression of telomere fusions was observed in *Upf1*^*HM*^ cells (Extended Data Fig. 5b,c).

To further evaluate whether expression of TRF1^ΔE8^ is sufficient to reduce TRF1 occupancy at telomeres and induce telomere fusions, we performed both ChIP (Fig. 5f–h) and IF (Extended Data Fig. 5h,i) for full-length TRF1 in cells expressing an ectopic TRF1 allele lacking exon 8. In both assays, we observed decreased levels of full-length TRF1 at telomeres, accompanied by a concomitant increase in telomere fusion events (Fig. 5f–i). These findings indicate that the TRF1^ΔE8^ isoform contributes to telomere fusions by reducing the effective TRF1 protection at chromosome ends.

Consistent with these findings, we depleted TRF2 and acutely degraded TRF1 using a dTAG degron strategy (Extended Data Fig. 4i,j, Extended Data Fig. 5d–g). In our setting, we observed high levels of telomere fusions in the double-depleted cells and high levels of γH2AX TIF-positive cells (Extended Data Fig. 5d–g), similarly to what was reported previously^11^. These results are consistent with the range of telomere fusions we detect in NMD-deficient cell lines (Fig. 2e and Extended Data Fig. 2b), supporting the idea that impaired TRF1 function contributes substantially to the observed telomere instability.

Collectively, these data demonstrate that, in the context of NMD deficiency, TRF2 loss leads to telomere fusions primarily due to reduced levels of TRF1 at telomeres. Consistent with this, increasing TRF1^FL^ levels is sufficient to prevent telomere fusions in TRF2/NMD codepleted cells. Furthermore, expression of the dominant-negative TRF1^ΔΕ8^ isoform is sufficient to trigger telomere fusions even in NMD-proficient cells. Thus, we conclude that the NMD pathway safeguards telomere integrity in ES cells by preventing the accumulation of the *Trf1*^*ΔE8*^ isoform, which displaces endogenous TRF1 from telomeres and compromises their protection. This is consistent with previous findings showing that the expression of a truncated dominant-negative TRF1 mutant inhibits the binding of full-length TRF1 to telomeres^44^. Interestingly, a dominant-negative allele of *Trf1* with mutated dimerization domain also interfered with telomeric localization of the full-length TRF1 in >80% nuclei^45^.

## Discussion

Here, we describe a role for the NMD pathway in telomere protection in mES cells. We found that, while depletion of TRF2 does not cause overt telomere deprotection in mES cells, its loss in NMD-deficient cells leads to the accumulation of DNA damage factors at telomeres and the frequent formation of end-to-end chromosomal fusions. While TRF2 depletion alone does not cause telomere deprotection in mES cells, its loss in NMD-deficient cells leads to DNA damage accumulation and frequent chromosome fusions, with individual NMD components contributing differentially to this process (Fig. 2e and Extended Data Fig. 2b).

Transcriptome analysis of cells depleted for SMG5, SMG6 and UPF1 identified a set of core NMD targets in mES cells, which was, as expected, highly enriched for transcript isoforms containing premature translation termination codons^46^. Strikingly, we found that *Trf1*, a critical telomere-associated protein, is strongly regulated by NMD in mES cells. In NMD-deficient cells, approximately 20–50% of *Trf1* transcripts skip exon 8, generating a truncated protein that lacks the C-terminal MYB DNA-binding domain but retains the TRFH dimerization domain. Our data suggest that this truncated isoform can heterodimerize with full-length TRF1, sequestering it away from telomeres in a dominant-negative manner. Consequently, TRF1 levels at telomeres are notably reduced in NMD-deficient cells (Fig. 5i). Based on ectopic expression experiments, we conclude that this truncated isoform is sufficient to account for the telomere deprotection phenotype observed in the absence of NMD. We propose a model in which the NMD pathway safeguards telomere integrity by preventing the accumulation of a dominant-negative *Trf1* transcript that produces a dominant-negative TRF1 protein. In this model, NMD deficiency lowers TRF1 availability at telomeres and, when combined with TRF2 loss, results in catastrophic telomere dysfunction and cell death.

These findings raise important questions regarding telomere homeostasis. One is whether modulation of NMD activity could provide a strategy to control TRF1 levels. Given that TRF1 acts as a negative regulator of telomerase activity^44,47,48^, transient suppression of NMD might be harnessed to enhance telomerase function in specific contexts. Supporting this idea, persistent DNA damage has been shown to suppress NMD activity^22^, suggesting a feedback mechanism to promote telomere healing through telomerase activation in cells experiencing telomere dysfunction.

Another notable implication is the unexpected observation that TRF1 and TRF2 have overlapping functions in mES cells, where TRF1 appears capable of compensating for TRF2 loss. This stands in sharp contrast to findings in somatic cells, where TRF2 is essential for telomere protection, and its depletion leads to extensive telomere deprotection despite the continued presence of TRF1^1,8,49^. This raises the question: why can’t TRF1 fulfil a similar protective role in somatic cells? One possibility is that TRF1 gains additional functions in mES cells via post-translational modifications or pluripotency-specific protein interactions, enabling it to promote T-loop formation or directly suppress ATM signalling functions typically attributed to TRF2. Alternatively, the high expression of TRF1 in pluripotent stem cells^50–52^ may allow it to compensate for TRF2 loss by sheer abundance. In this model, TRF1 may possess a weak intrinsic ability to substitute for TRF2, which becomes functionally relevant only when TRF1 is expressed at high levels.

Finally, our data provide an intriguing connection between the NMD pathway and genome integrity. Several studies have implicated the NMD pathway in regulatory functions in stem cell biology and genome stability^20,22,23,26,27,53^. Notably, NMD has been implicated in telomere biology in mammalian cells through its function in controlling a telomeric repeat-containing RNA (TERRA) that accumulates in cancer cells utilizing alternative lengthening of telomere, and plays roles in telomere homeostasis and DNA damage signalling^16,54–56^. Our data add a new piece to this puzzle showing that, in mES cells, the NMD pathway plays an essential role in ensuring TRF2-independent telomere protection. Our study identifies a synthetic lethal interaction between TRF2 and NMD, revealing how post-transcriptional regulation controls telomere protection in pluripotent stem cells.

## Methods

### ES cell derivation and culture

*Trf2*^*f/*f^*-Rosa26-creER* ES cell derivation and growth conditions were previously described^10^. To induce Cre activity, 4-hydroxytamoxifen (OHT; 0.6 nM; Sigma) was added 96 h before cell collection unless otherwise specified. Where indicated, cells were treated with the NMD inhibitor 11j (1 µM; Fisher Scientific, 50-225-9662), the dTAG-13 degrader (0.5 µM; MilliporeSigma, SML2601-1MG) or DOX (500 ng ml^−1^, MilliporeSigma, D9891) for 48 h before collection. To generate knockout cell lines, ES cells were nucleofected (Mouse Embryonic Stem Cell Nucleofector Kit, Lonza, VPH-1001) with a cocktail containing three synthetic gRNAs (GKOv2 kits, Synthego, now EditCo) and Cas9 Nuclease V3 (100 µg; IDT, 1081060). Clonal lines were isolated, screened by PCR and validated by Sanger sequencing and immunoblotting. Primers used for PCR amplification were

*Smg5* - forward (F): 5′- GAGCTTGTCACATGAGAGGTCT-3′

*Smg5* - reverse (R): 5′- ACTCGCACCCATTTGGAGAG-3′

*Smg6* - forward (F): 5′- CCTTTGGGACCTCGACTTTT-3′

*Smg6* - reverse (R): 5′- TTGCTGCATGTTCCGCAC-3′

*Smg7* - forward (F): 5′- CTCCAAAGAGCTGCCTTAGGT-3′

*Smg7* - reverse (R): 5′- GGCTTTGCTTTGGTGGATGG-3′

*Smg8* - forward (F): 5′- CCATACCTCCGCGAAGTGAA-3′

*Smg8* - reverse (R): 5′- GCGGAGGAGGGATTTCACAA-3′

*Smg9* - forward (F): 5′- GCCTCTGGTTTGTTTGTGGG-3′

*Smg9* - reverse (R): 5′- TGAGCCCACCTCCCCTTTAT-3′

*Upf1* - forward (F): 5′- TCTAACTGGGACCTGGCTCA-3′

*Upf1* - reverse (R): 5′- CTCAGAGCTCAGAACCGGC-3′.

Two independent clones were analysed per gene. dTAG cell lines were established as previously described^39^. N-terminal FKBP12^F36V^–FLAG fusions were introduced at the endogenous *Upf1* and *Trf1* loci. Degradation of the fusion proteins was confirmed by treatment with dTAG-13 (0.5 µM).

### Cell growth analysis

Cell proliferation was monitored in real time using the IncuCyte S3 system (Essen Bioscience) based on confluence measurements. ES cells were imaged every 3 h with a 10× objective. At least two biologically independent replicates were analysed per condition.

### Clonogenic assay

ES cells were dissociated into a single-cell suspension and seeded at low density (500 cells per well in a 6-well plate). Cells were cultured undisturbed for 7 days to allow colony formation, then fixed with methanol and stained with crystal violet.

### DOX-inducible Trf1 expression from PiggyBac vectors

PiggyBac PB-TRE-mTRF1^FL^ or PB-TRE-mTRF1^ΔE8^ constructs were cloned from the PB-TRE-dCas9-VPR plasmid (63800, Addegene)^12^. ES cells were cotransfected with the PiggyBac and a transposase-expressing plasmid using Lipofectamine 2000 (Thermo Fisher Scientific, 11668019) for stable integration.

### Western blotting

Cells were lysed in 2× Laemmli buffer and proteins separated by sodium dodecyl sulfate–polyacrylamide gel electrophoresis on 4–20% TGX stain-free gels (Bio-Rad, 4568093). Proteins were transferred to nitrocellulose membranes and probed with antibodies against SMG5 (1:1,000; Abcam, ab33033), SMG6 (1:1,000; Abcam, ab87539), SMG7 (1:1,000; Bethyl Laboratory, A302-170A), SMG9 (1:1,000; Abcam, ab85659), UPF1 (1:1,000; Bethyl, A301-902A), TRF1 (1:200; Abcam, ab192629), actin (1:1,000; MilliporeSigma, A5441) or tubulin (1:5,000; MilliporeSigma, T5168). Detection used horseradish peroxidase- or DyLight-conjugated secondary antibodies and the ChemiDoc MP system (Bio-Rad).

### IF and IF–fluorescence in situ hybridization (FISH)

Cells were fixed with 2% paraformaldehyde and stained with primary antibodies (1:1,000): OCT3/4 (Santa Cruz, sc-5279), γH2AX (Millipore, 05-636), 53BP1 (Novus, NB100-304), FLAG (MilliporeSigma, F1804) and MYC (Cell Signaling, 2276). After secondary antibody incubation, cells were post-fixed, denatured at 72 °C and hybridized with AlexaFluor 488-TelC PNA probe (PNA Bio, F1004). Slides were mounted with ProLong Gold antifade (Thermo Fisher, P36931), and images were acquired using a Zeiss Axio Imager M2 and Axiocam 702 with ZEN 2.6 software. *Z*-stacks were displayed as maximum intensity projections. Figures were assembled with Adobe Illustrator 2024.

### FISH on metaphase spreads

Cells were treated with colcemid (0.2 µg ml^−1^, 2 h), swelled in 75 mM KCl, fixed in methanol:acetic acid (3:1) and dropped onto glass slides. Telomeres were detected using AlexaFluor 488-TelC PNA probe and counterstained with DAPI. Images were acquired using a Zeiss Axio Imager.

### Statistics and reproducibility

Statistical analyses were performed using GraphPad PRISM version 9.0 software (GraphPad). Data represent the mean ± s.d. of three independent experiments, unless stated otherwise. *P* ≤ 0.05 was considered statistically significant, and *P* values were assessed by one-way analysis of variance (ANOVA) followed by multiple comparisons. Sample size was not predetermined. For experiments involving quantification of positive cells (for example, OCT4 or TIFs positive) or chromosome fusions, *n* = 3 biological replicates was chosen as the minimal replicate number, and the sample size was determined by the number of positive cells within the replicates. We determined this to be sufficient owing to internal controls and low observed variability between stained samples. Data were not excluded from analysis. All replication attempts were successful, and observed data were consistent with orthogonal methods and previously known results. Cells and chromosomes used for imaging were selected randomly and analysed equally with no subsampling. Blinding was applied to all the images before quantification.

### CRISPR–Cas9 screen

Three independent genome-wide CRISPR screens were performed using a genome-wide CRISPR knockout library (73633-LVC, Addgene) in *Trf2*^−/−^ and *Trf2*^*f/−f*^ ES clones expressing Cas9 (no. 52962, Addgene), as described previously^10^. Following selection, a portion of cells was collected as the initial timepoint (T0), and the remaining cells were cultured for 14 days before collection as the final timepoint (T14). Genomic DNA was isolated using a DNeasy Blood and Tissue Kit (Qiagen, 69506), and single guide RNAs (sgRNAs) were PCR-amplified using primers containing Illumina adapters. Samples were sequenced on an Illumina NextSeq platform, and the resulting reads were analysed using MAGeCK^57^. An essentiality score (*β* score) for each gene was calculated using the MAGeCK -mle module. Average *β* scores were calculated from three independent experiments. Potential synthetically lethal genes were: (1) having a negative (<−0.75) *β* scores in the *Trf2*^−/−^ cells; (2) having a ‘neutral’ *β* score (>−0.25 and lower than 1) in the control *Trf2*^*−/f*^ ES clones; and (3) having a difference in *β* scores (control – OHT) greater than 1 in all the experiments (Supplementary Tables 1 and 4). Data were plotted using ggplot2.

### RNA extraction and RT–qPCR

RNA was extracted from 1 × 10^6^ cells using the RNeasy Plus Mini Kit (Qiagen, 74136). Then, 500 ng of RNA was reverse transcribed using PrimeScript IV 1st strand cDNA Synthesis Mix (TakaraBio, 6215A). SYBR Green PCR Master Mix (ThermoFisher, AppliedBiosciences, 4368706) was used for quantitative PCR. Primers used were

*Trf1* (exon 7) - forward (F): 5′-TGTTAATGGCCAGCAGTCT-3′

*Trf1* (exon 7/exon 9 junction) - reverse (R): 5′-CATCGTTGTTTCACCTTATTGAGGA-3′

*Trf1* (exon 9) - reverse (R): 5′-TCCACTGGTTCTTCGGTTCC-3′

*Gapdh* forward (F): 5′-TGTGTCCGTCGTGGATCTGA-3′

*Gapdh* reverse (R): 5′-TTGCTGTTGAAGTCGCAGGAG-3′.

### ChIP assay

*Trf1*^*dTAG*^ (control and 11j-treated ES cells for 48 h) and *Trf1*^*dTAG*^ *Smg5*^−/−^ samples were cross-linked using 1% formaldehyde (Thermo Fisher Scientific, cat. no. 28908) at room temperature for 3 min. The reaction was quenched with 125 mM glycine, and cells were collected by centrifugation at 250*g* for 5 min at 4 °C and sonicated using the Bioruptor sonication system Covaris S220 sonicator (Covaris). Chromatin from 4 × 10^6^ cells was used for immunoprecipitation.

In total, 30 μg of clear chromatin was incubated with Dynabeads Protein A (Thermo Fisher, cat. no. 10002D) or Dynabeads Protein G (Thermo Fisher, cat. no. 10003D) together with either 4 μg of mouse monoclonal FLAG antibody (F1804, MilliporeSigma) or 2 μg of H3 antibody (ab1791, Abcam). Inputs correspond to the 0.5% or 1% fraction of the total DNA sample used in the immunoprecipitation. The precipitated DNA was eluted and transferred to a Hybond+ membrane by dot blotting. The membrane was then hybridized with a P^32^-labelled telomeric probe recognizing TTAGGG repeats and visualized with the PhosphorImager (Typhoon biomolecular imager). The signals were quantified using ImageJ software.

### Apoptosis and cell cycle analysis

Apoptosis was assayed using the Annexin-V Apoptosis Detection Kit (Abcam, ab14150) according to the manufacturer’s instructions. In brief, cells (1 × 10^6^) were collected by centrifugation and resuspended in 500 µl of the supplied Binding Buffer II. Annexin V (5 µl) was added to each sample, followed by incubation for 5 min at room temperature in the dark. Fluorescence was analysed by flow cytometry using Cy5 excitation/emission settings on CytoFLEX S Flow Cytometer (CytoFLEX S). Cells positive for Annexin V–Cy5 were interpreted as apoptotic.

Cell-cycle distribution was assessed by propidium iodide (PI) staining of fixed cells. In brief, cells were collected, washed with phosphate-buffered saline and fixed in 70% ice-cold ethanol. Samples were stored at −20 °C for more than 2 h. After fixation, cells were washed with phosphate-buffered saline and incubated for 30 min at room temperature in PI staining solution (50 µg ml^−1^) with RNase A 100 µg ml^−1^. Samples were analysed by flow cytometry using a 488-nm laser, and DNA content was quantified in the PI channel. Cell-cycle phases (G1, S and G2/M) were determined using FlowJo software.

### Total RNA-seq

RNA extractions of control *Trf2*^*f/f*^ ES cells as well as *Smg5*^−/−^*, Smg6*^−/−^ and *Upf1*^*HM*^ were performed using RNeasy Plus Mini Kit (Qiagen) following the manufacturer’s protocol. Total RNA was then ribo-depleted, followed by library preparation using the Illumina Stranded Total RNA Prep. Paired-end libraries were pooled and sequenced on NovaSeq 6000 SP. The samples had 99–140 million pass filter reads with more than 90% of bases above the quality score of Q30. Reads of the samples were trimmed for adapters and low-quality bases using Cutadapt. The initial alignment was performed using STAR (v.2.7.6a)^58^ with reference genome mm10 and GENCODE vM27 annotation.

### Long-read annotation filtering

The long-read transcript annotation for mES cells (ES_consolidated.bed) from Pardo-Palacios et al.^59^ was obtained and converted to GTF format using UCSC Genome Browser utilities (bedToGenePred)^60^. The resulting GTF file was then filtered to retain only full splice match isoforms using a custom R script.

### Transcript assembly and filtering

Transcriptomes were assembled from individual mouse ES cell RNA-seq samples using StringTie (v.2.1.4)^61^. For each sample, alignment files in BAM format were processed using StringTie with a minimum isoform fraction threshold of 0.05 (-f 0.05) and guided by the previously filtered GTF annotation reference (-G ES_consolidated.gtf). Trimming was disabled (-t) to preserve full-length transcript structures. Assembled transcriptomes were filtered to retain only isoforms with an expression level greater than 1 transcripts per million using a custom R script (StringTie_filter_gtf.R). Filtered sample-level annotations were merged in two stages using StringTie’s–merge function: first, within each experimental condition (Smg5^−/−^, Smg6^−/−^, Upf1^HM^ and control) and then across all conditions to generate a unified annotation. The merged GTF file was sorted and indexed using IGVTools^62^. Unstranded transcripts were removed using a custom filtering script (Filter_gtf_strand.R).

### Gene model refinement and annotation

To refine transcript models and remove likely spurious or low-abundance isoforms, we applied the AnnotationCleaner pipeline^63^ (https://github.com/isaacvock/AnnotationCleaner). This filtering step produced a high-confidence annotation, which was then used to predict open reading frames and identify PTCs using factR2^64^.

### Short-read RNA-seq alignment, quantification and differential expression analysis

A STAR index was generated from the cleaned annotation, and raw FASTQ files were aligned to the custom transcriptome using STAR (v.2.7.6a)^58^. Isoform- and gene-level expression estimates were then quantified using RSEM (v.1.3.3)^65^ with corresponding custom indices. Transcript-level expression data were normalized, and log_2_ fold changes (L2FC) in RNA abundance were calculated by Degust (https://degust.erc.monash.edu/; R script provided). Significant events were called at |L2FC| >1, *P*_adj _< 0.05 in *Upf1*^*HM*^, *Smg5*^−/−^ and *Smg6*^−/−^ relative to wild-type ES cells. Finally, L2FC (RNA abundance) values were integrated with PTC annotations to facilitate downstream analysis of isoform-specific regulation and NMD sensitivity (Supplementary Table 2).

### Differential splicing analysis

Alternative splicing analysis was performed using the SpliceWiz R package^66^, beginning with the construction of a reference from the GENCODE vM27 annotation and mm39 genome. Aligned BAM files from mES cell samples were processed to quantify splicing events, followed by novel splice junction discovery using collateData(novelSplicing = TRUE, novelSplicing_requireOneAnnotatedSJ = TRUE, novelSplicing_minSamples = 3, novelSplicing_minSamplesAboveThreshold = 3, novelSplicing_countThreshold = 10, novelSplicing_useTJ = TRUE) with stringent filtering parameters. The resulting experiment was imported as a NxtSE object, and alternative splicing events were filtered for high-confidence features (default filters). Differential splicing analysis was then conducted using the ASE_DESeq(IRmode = "all") function, identifying significant percent spliced in (PSI) events (|ΔPSI| >0.15, *P*_adj_ < 0.05) in *Upf1*^*HM*^, *Smg5*^−/−^ and *Smg6*^−/−^ relative to wild-type ES cells (Supplementary Table 3).

### Reporting summary

Further information on research design is available in the Nature Portfolio Reporting Summary linked to this article.

## Online content

Any methods, additional references, Nature Portfolio reporting summaries, source data, extended data, supplementary information, acknowledgements, peer review information; details of author contributions and competing interests; and statements of data and code availability are available at 10.1038/s41556-026-01912-0.

## Supplementary information

Supplementary Information Supplementary Table 1. Gene ranking based on differential *β* score. Average *β* scores for each gene targeted in the gRNA library were calculated for TRF2-proficient and TRF2-deficient cells using the MageCK MLE module. The table lists genes ranked by their differential beta score, reflecting relative enrichment or depletion between the two conditions. Supplementary Table 2. Differential transcript expression upon NMD factor depletion. Transcript-level differential expression analysis following Smg5, Smg6 and Upf1 depletion was performed using RNA-seq data from three biologically independent experiments. L2FC, false discovery rate (FDR), average expression (AveExpr) and *P* values were calculated relative to wild-type controls. Novel transcripts were identified using StringTie. For each transcript, additional information includes: presence of a coding sequence (CDS), predicted NMD sensitivity, and key 3′ untranslated region (UTR) features—distance from the stop codon to the last exon–exon junction, number of downstream exon junctions and 3′ UTR length. Where applicable, the genomic coordinates of the PTC are indicated. The table includes two tabs: • Differentially_expressed_genes: all differentially expressed transcripts identified in any of the three NMD-depleted conditions. • *P* < 0.05_L2FC > 1.5: Subset (501) transcripts showing significant differential expression (*P* value <0.05 and |L2FC| >1.5) across all three NMD-depleted cell lines. Supplementary Table 3. Differential alternative splicing events common among NMD factor depletions. The table includes events that were significantly altered (|ΔPSI| >0.15, adjusted *P* value <0.05) in *Upf1*^*HM*^, *Smg5*^−/−^ and *Smg6*^−/−^ embryonic stem cells relative to wild-type controls. Supplementary Table 4. Read counts. This table reports raw sgRNA read counts from two biologically independent CRISPR knockout screens (E1 and E2). Each row corresponds to a single sgRNA and the gene it targets. Read counts are shown for three cell lines: A7 and D2, which are TRF2-deleted experimental clones, and E8, which is the TRF2-proficient control. For each biological replicate, samples were collected at baseline (0 days) and after 14 days of culture. These read counts form the input matrix for downstream MAGeCK analysis used to quantify sgRNA enrichment or depletion in TRF2-deleted versus TRF2-proficient backgrounds.

Reporting Summary

## Source data

Source data Fig. 1 Statistical source data.

Source data Fig. 2 Unprocessed western blots and/or gels, and images.

## Data Availability

The raw and processed RNA-seq data supporting Fig. 4 have been deposited in the NCBI Gene Expression Omnibus (GEO) and are accessible via accession number GSE300187. Source data are provided with this paper.
