# Supplementary material for: Nonsense-mediated mRNA decay safeguards telomeres in pluripotent stem cells
Source: Nat Cell Biol. 2026 Mar 24;28(4):674–83. doi: 10.1038/s41556-026-01912-0 (PMC13086586; doi:10.1038/s41556-026-01912-0)

Figure 1b Source Data

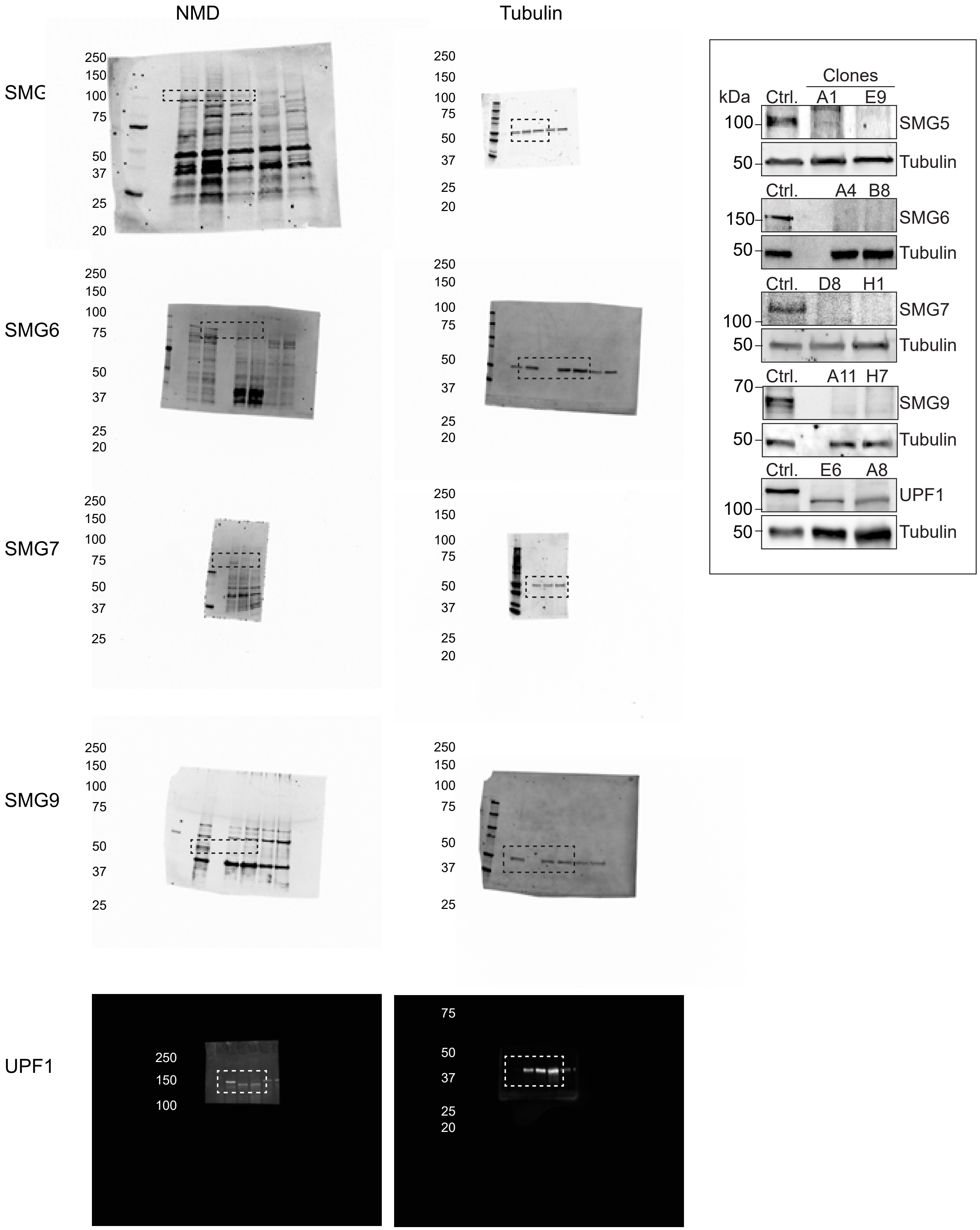

Figure 1e Source Data

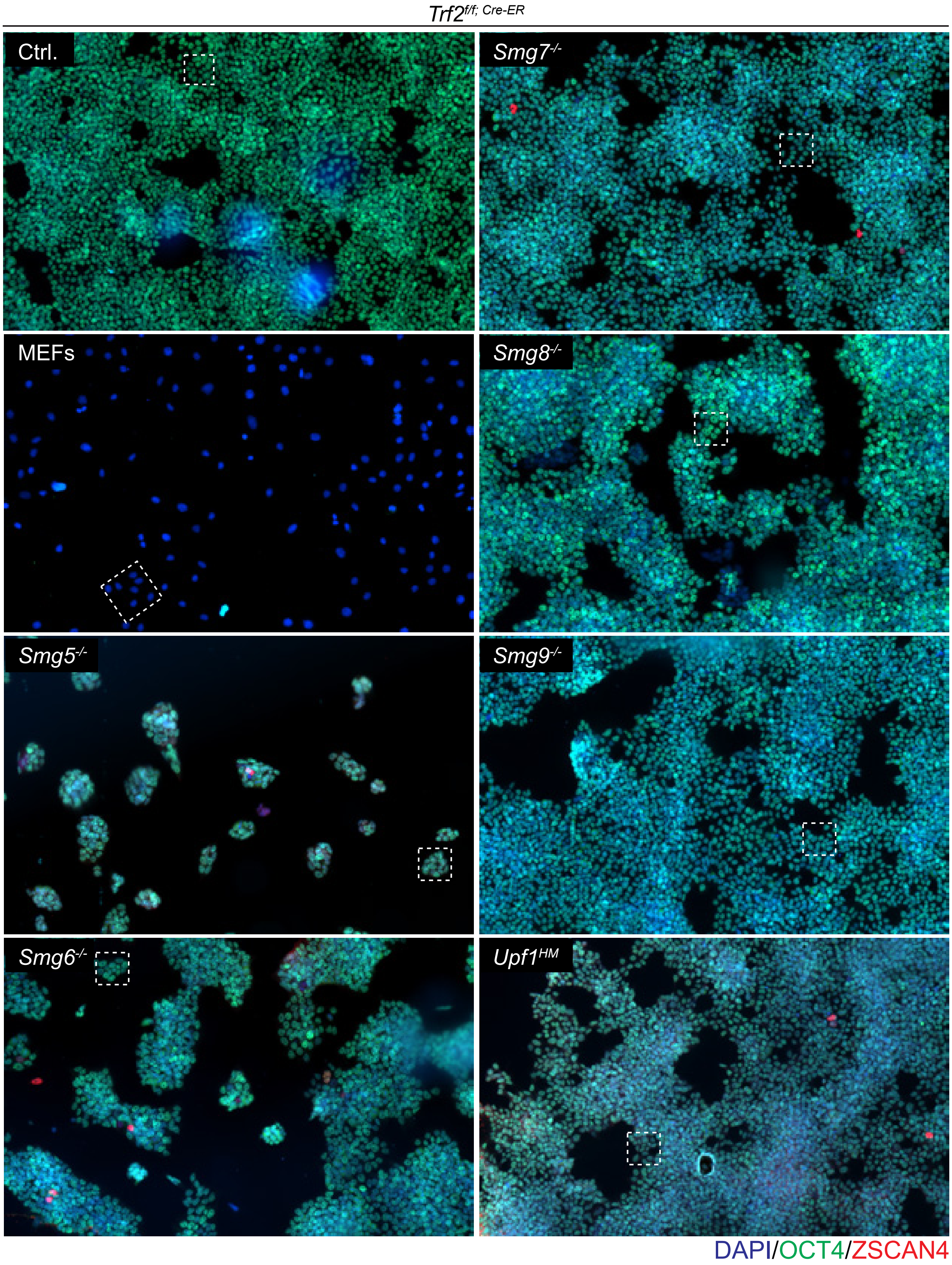

Figure 2a Source Data

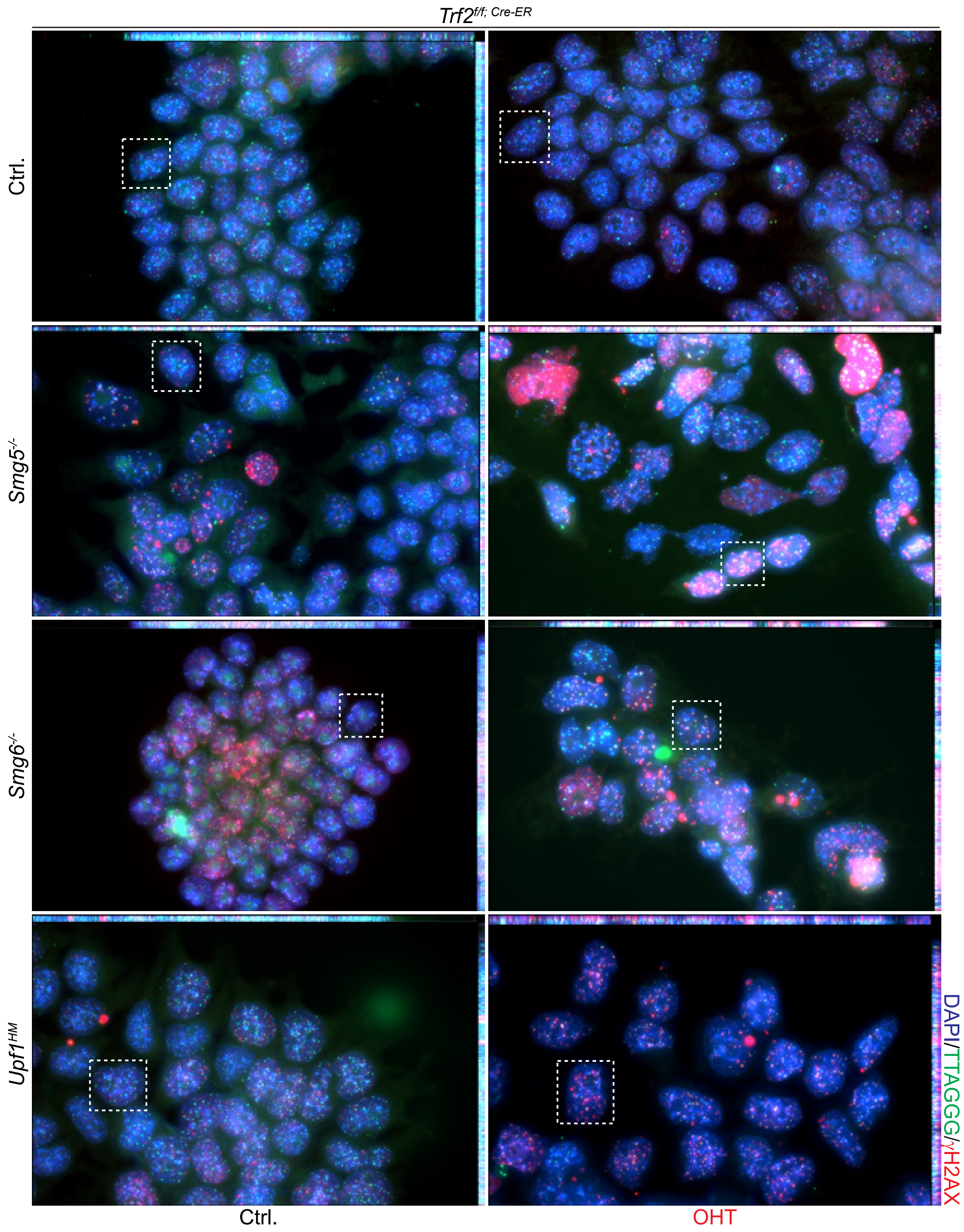

Figure 2c Source Data

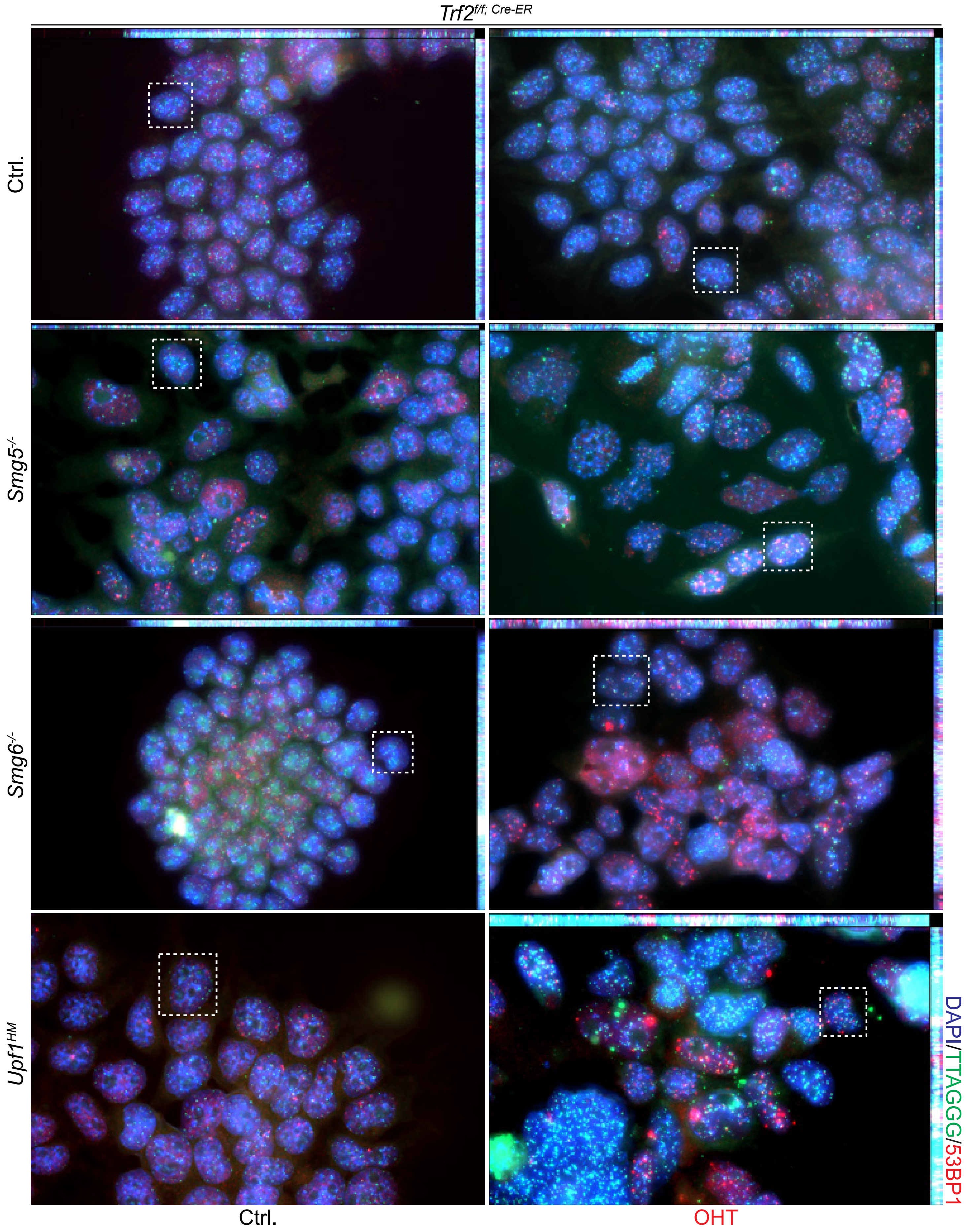

Figure 2e Source Data

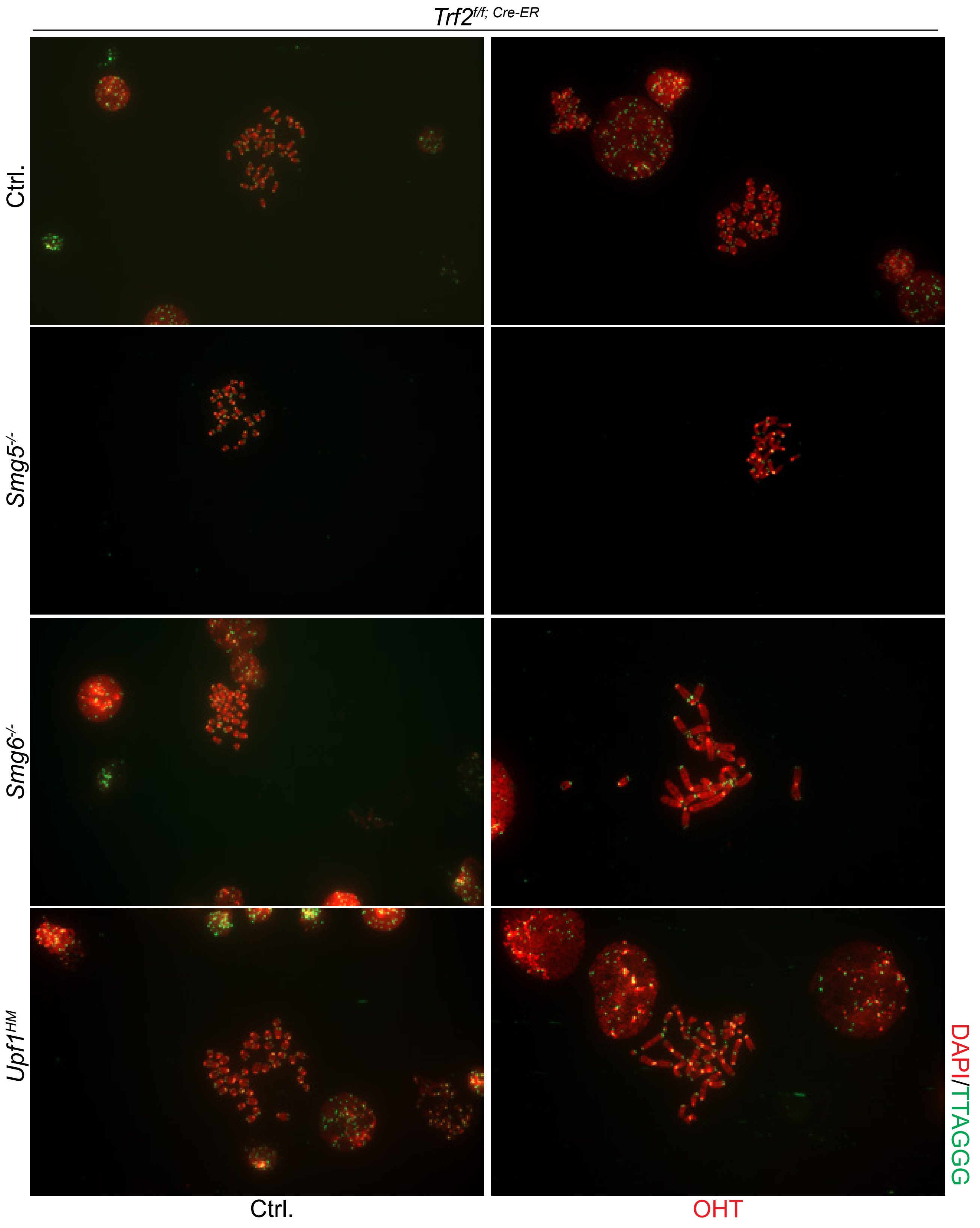

Figure 2e Source Data cont.

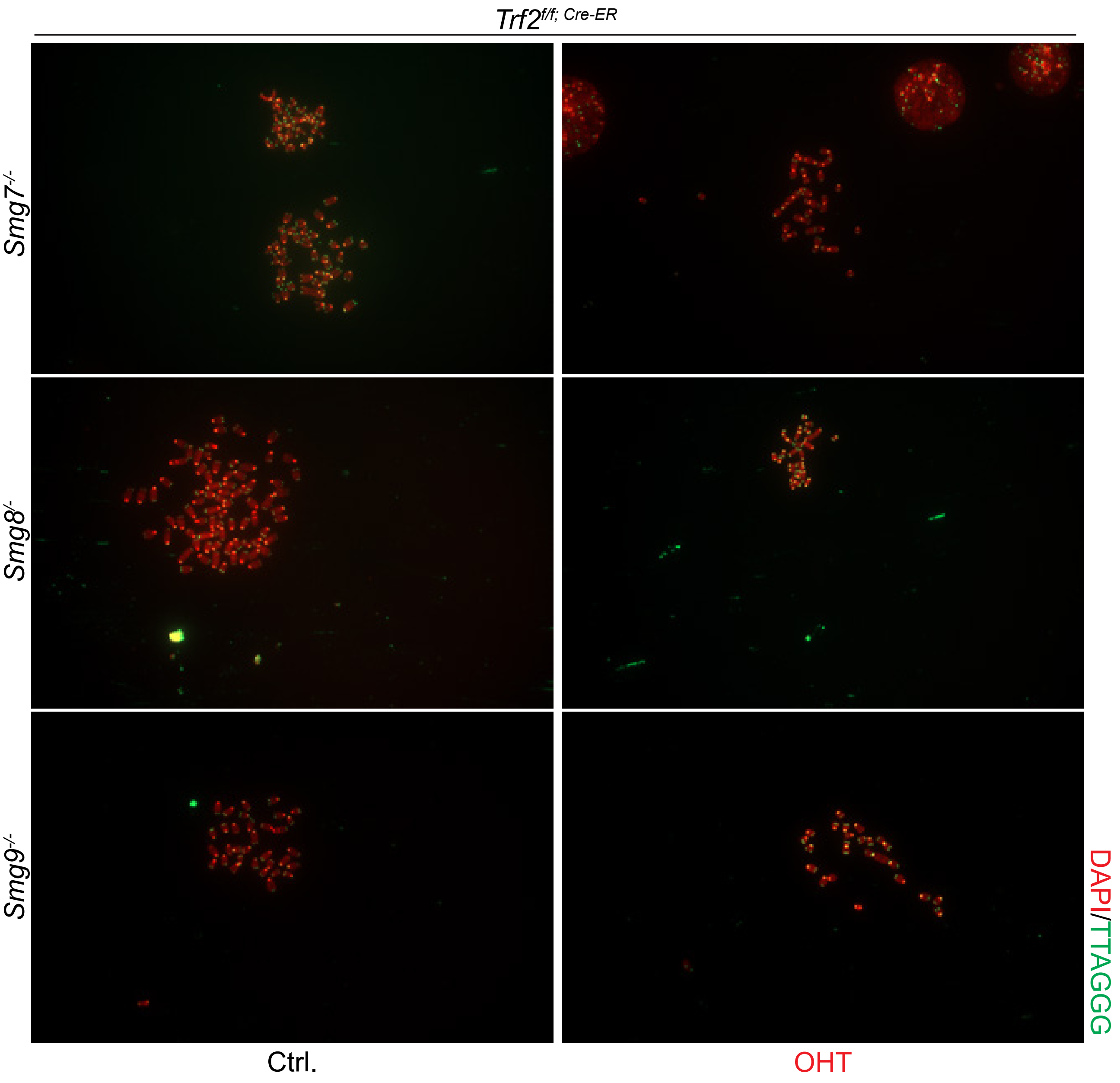

Figure 3a Source Data

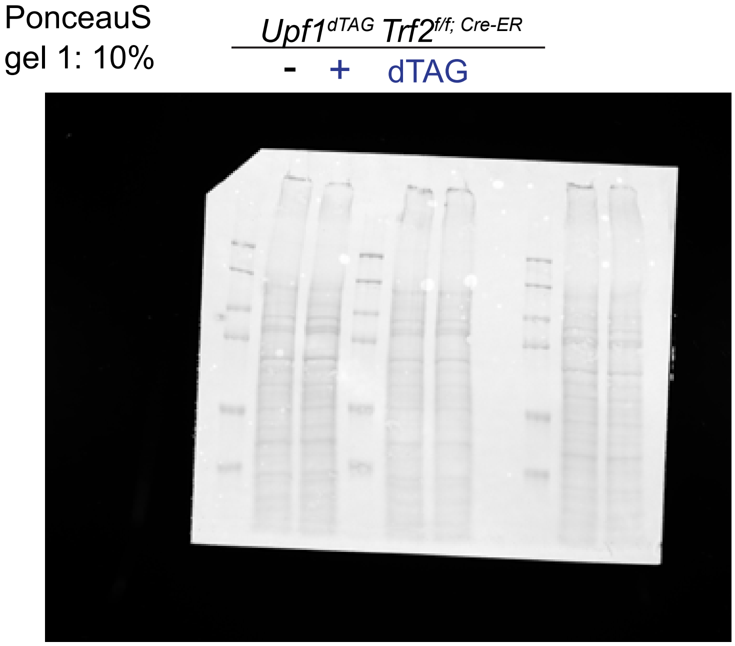


FLAG

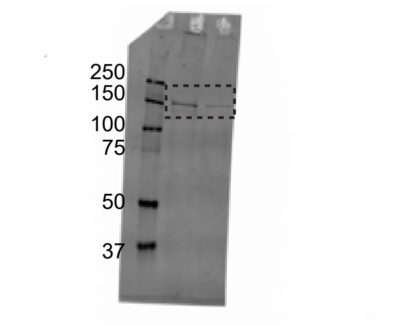


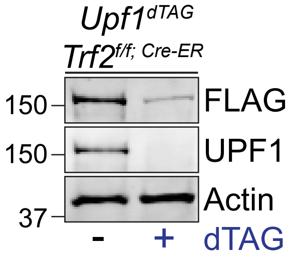


Upf1

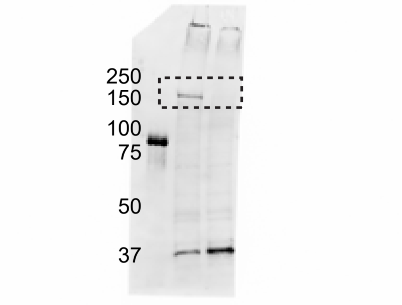


Actin

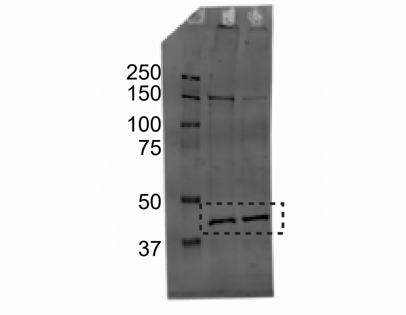

Figure 3b Source Data

*$Upf1^{dTAG}$ $Trf2^{f/f;\ Cre\text{-}ER}$*

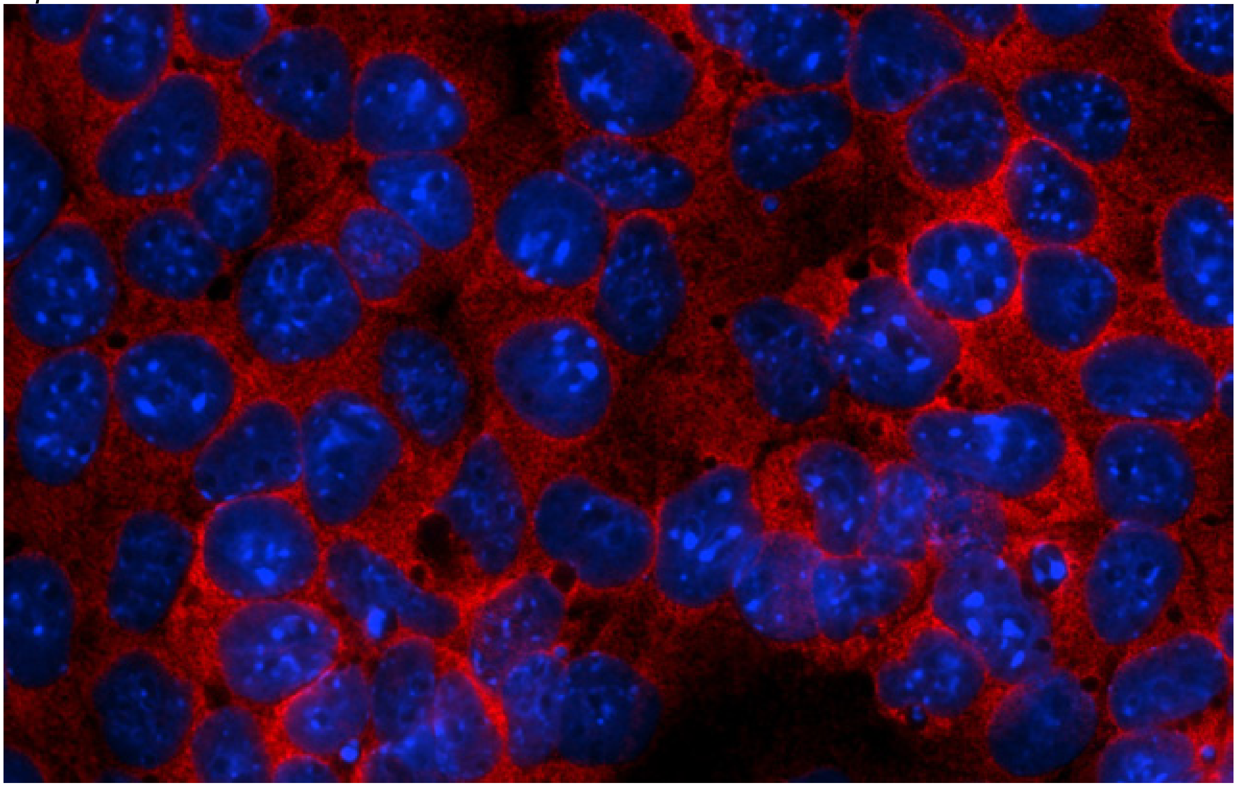

*$Upf1^{dTAG}$ $Trf2^{f/f;\ Cre\text{-}ER}$ +dTAG*

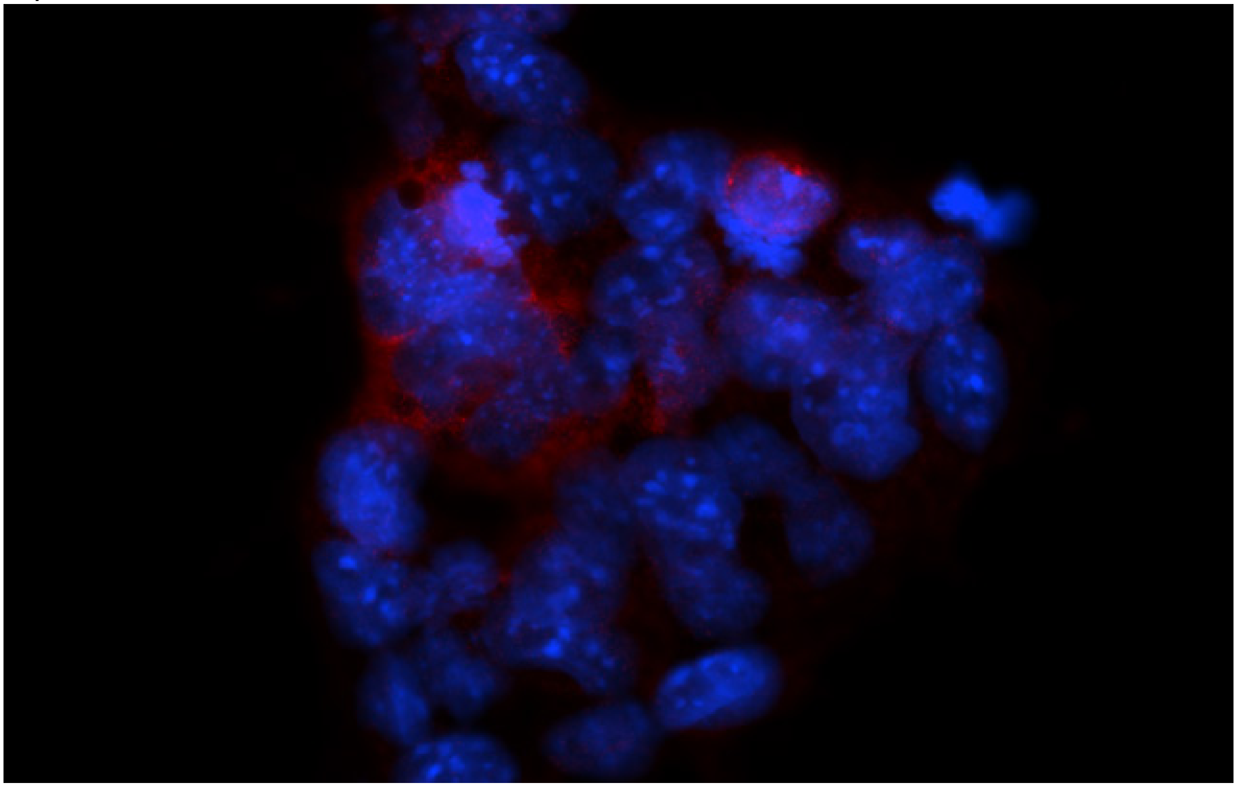

DAPI/Upf1-dTAG-FLAG

Figure 3c Source Data

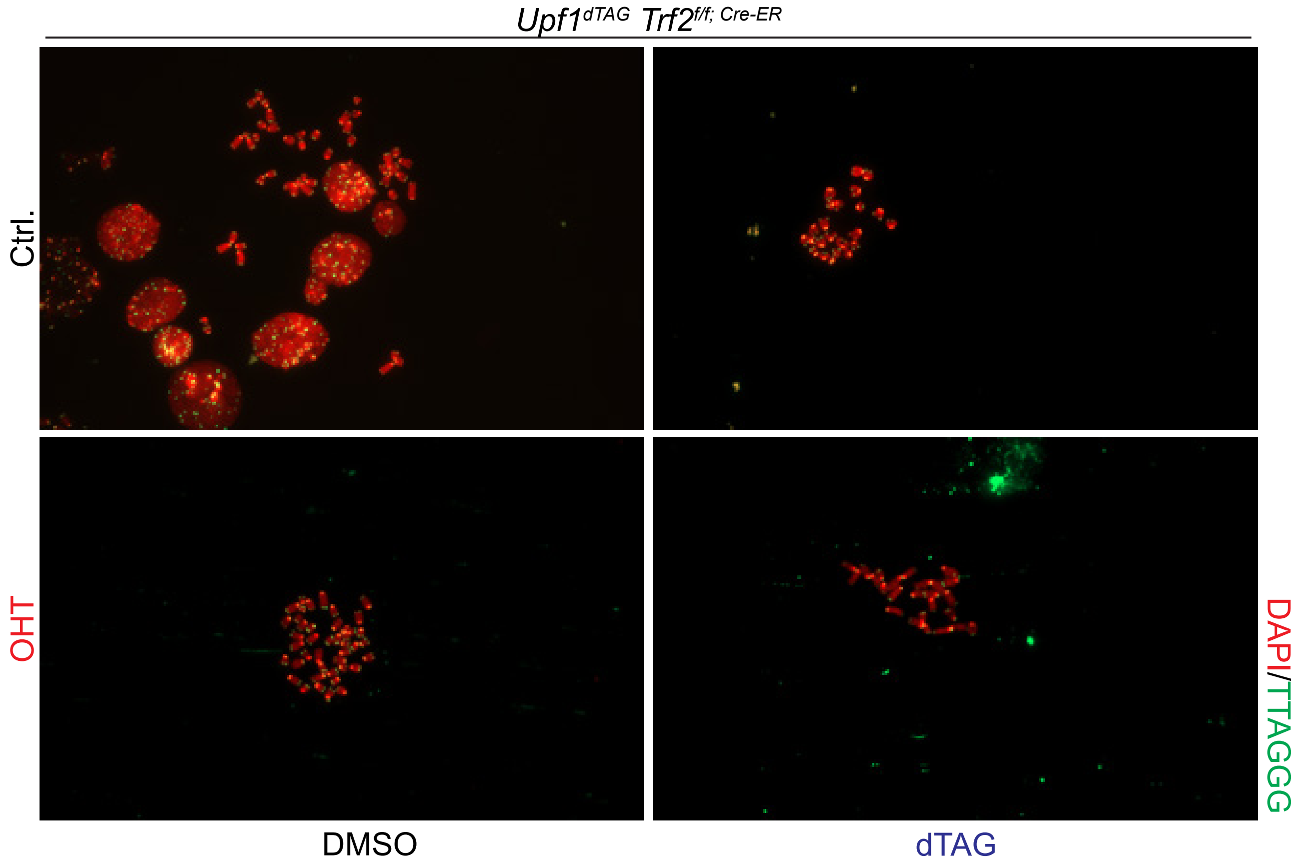

Figure 3e Source Data

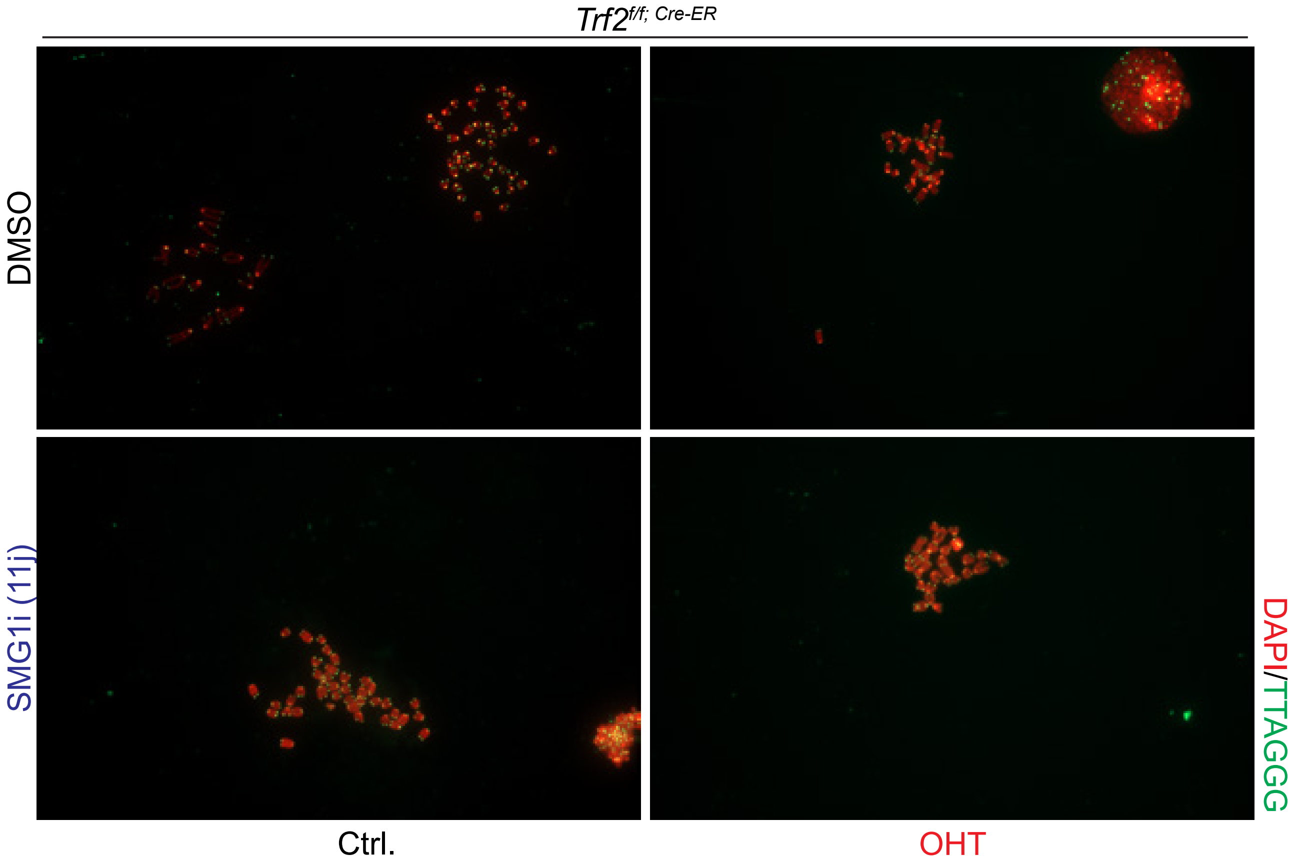

Figure 3g Source Data

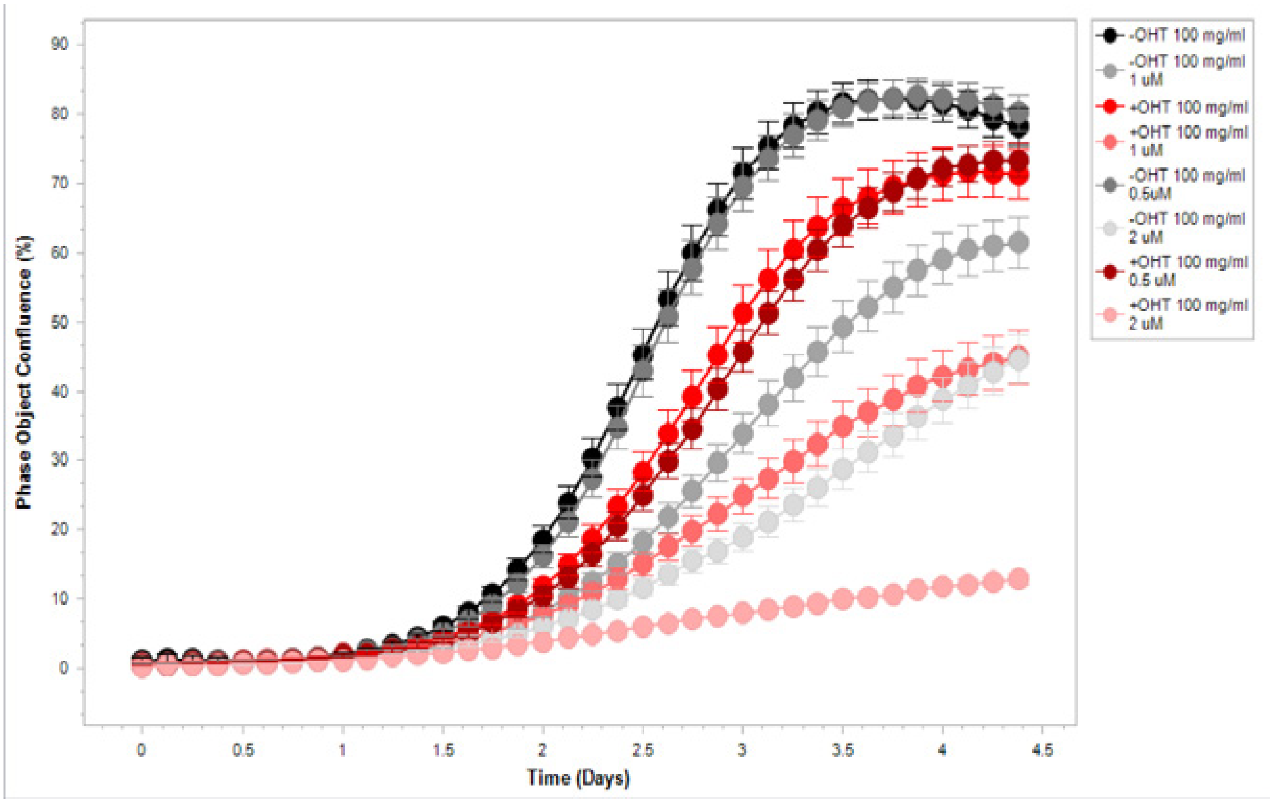

Figure 4g Source Data

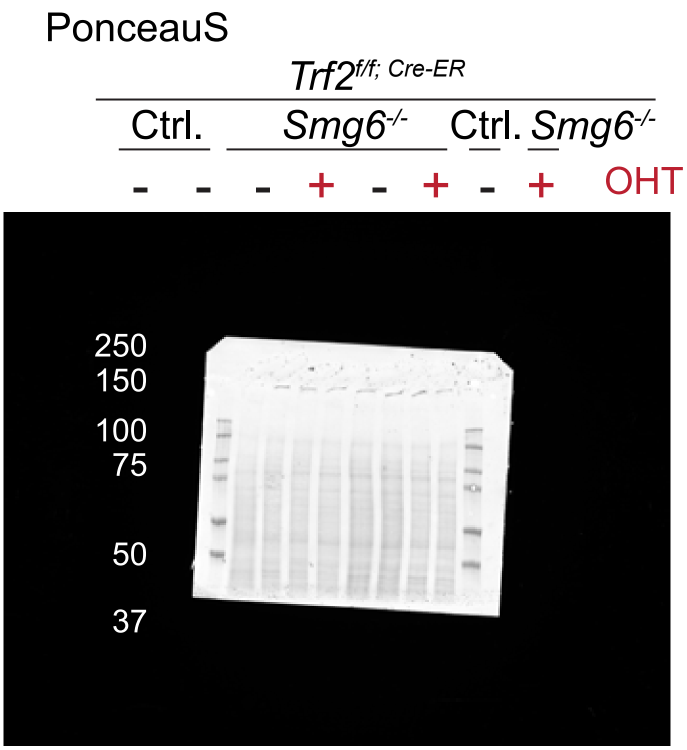


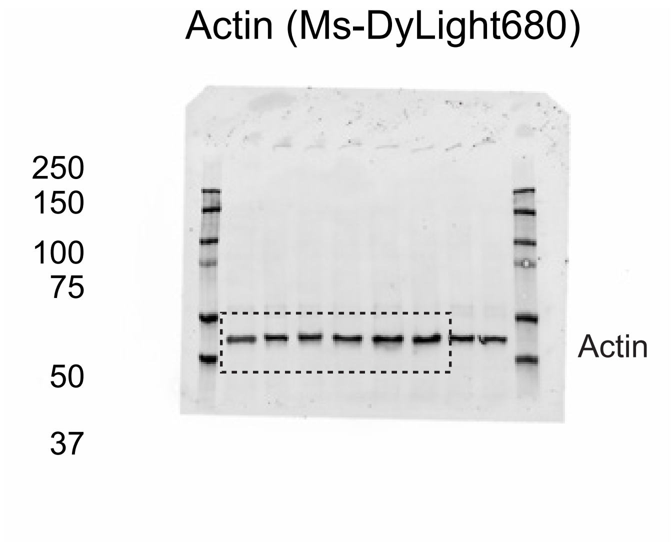


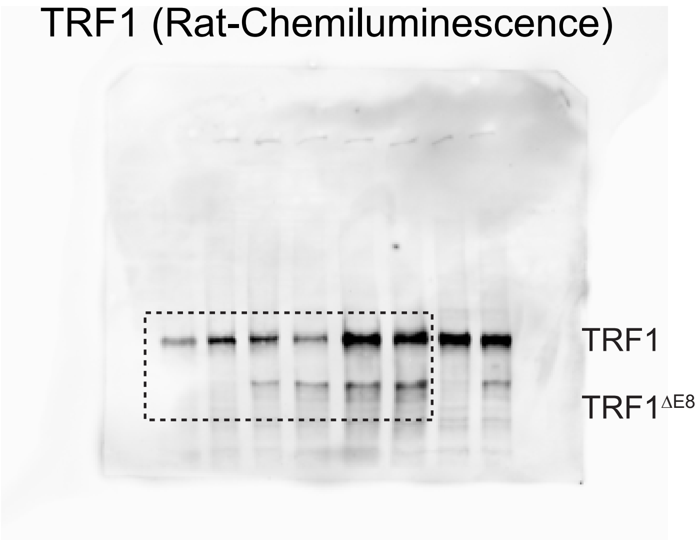


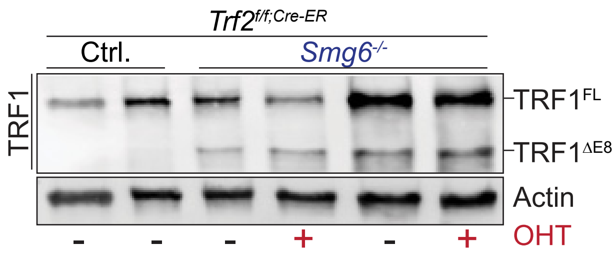

Figure 4h Source Data

**PonceauS**

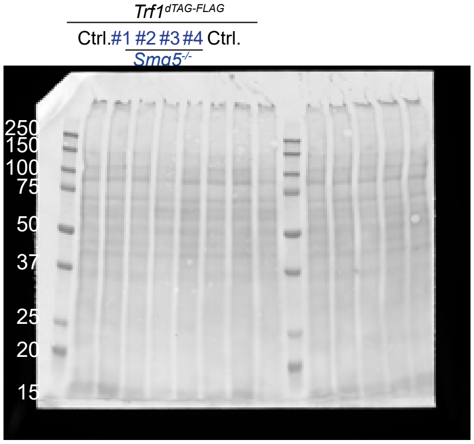


**Tubulin (Ms)**

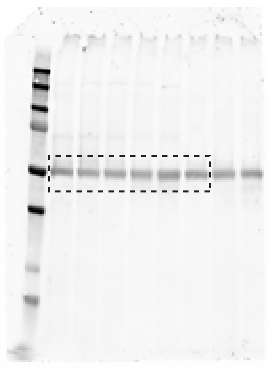

**FLAG-TRF1 (Ms)**

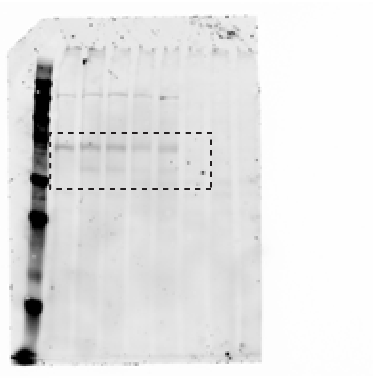

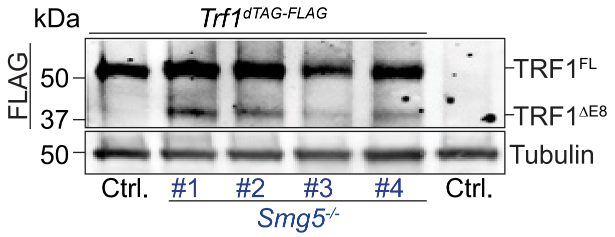

Figure 4i-j Source Data

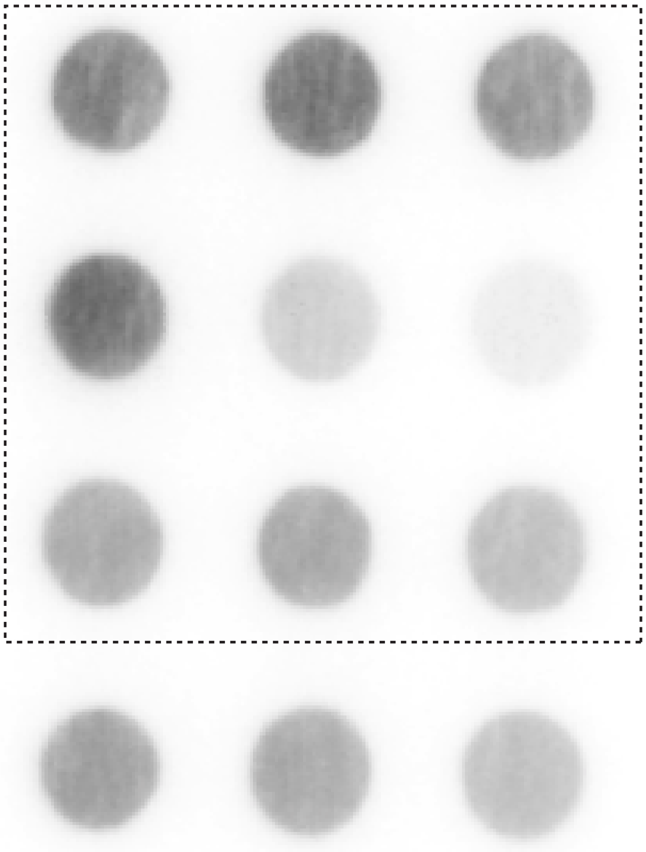

# Figure 5b Source Data

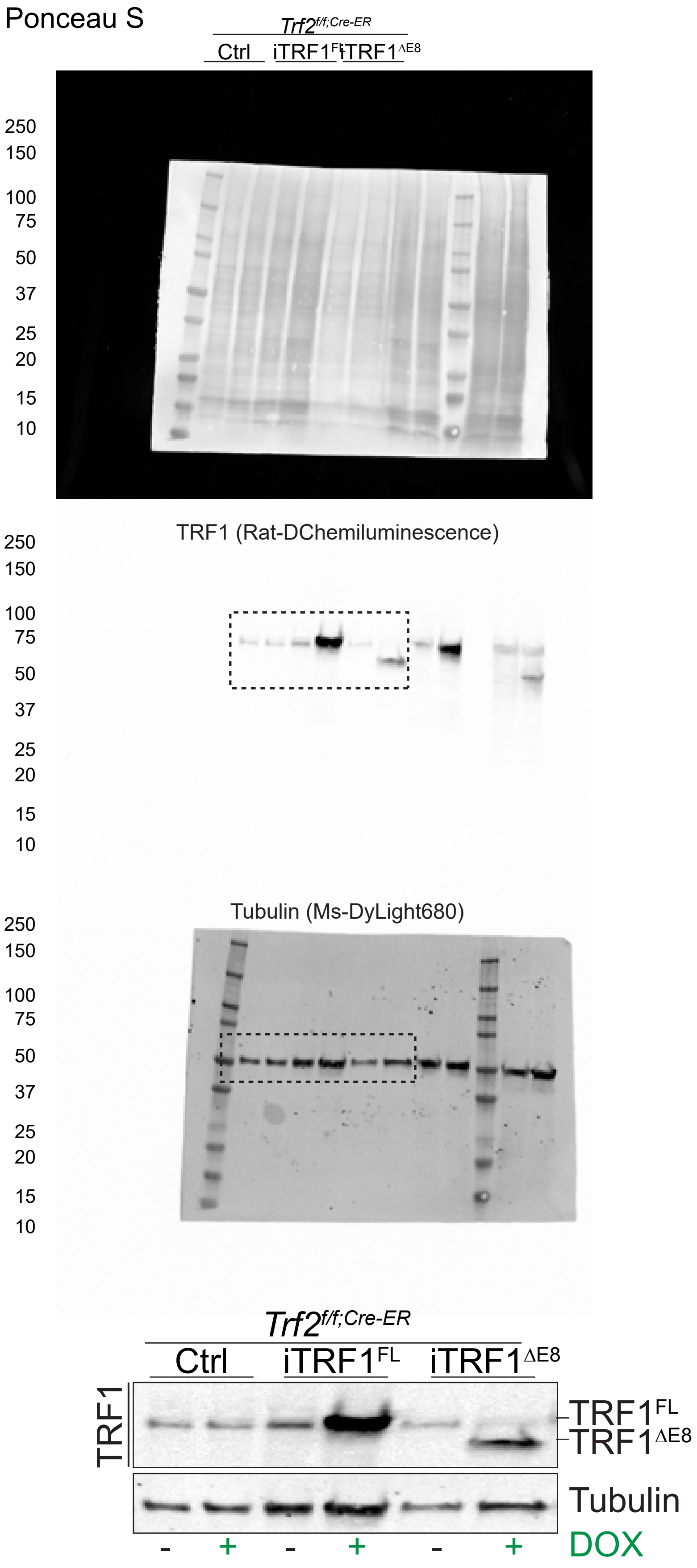

Figure 5c Source Data

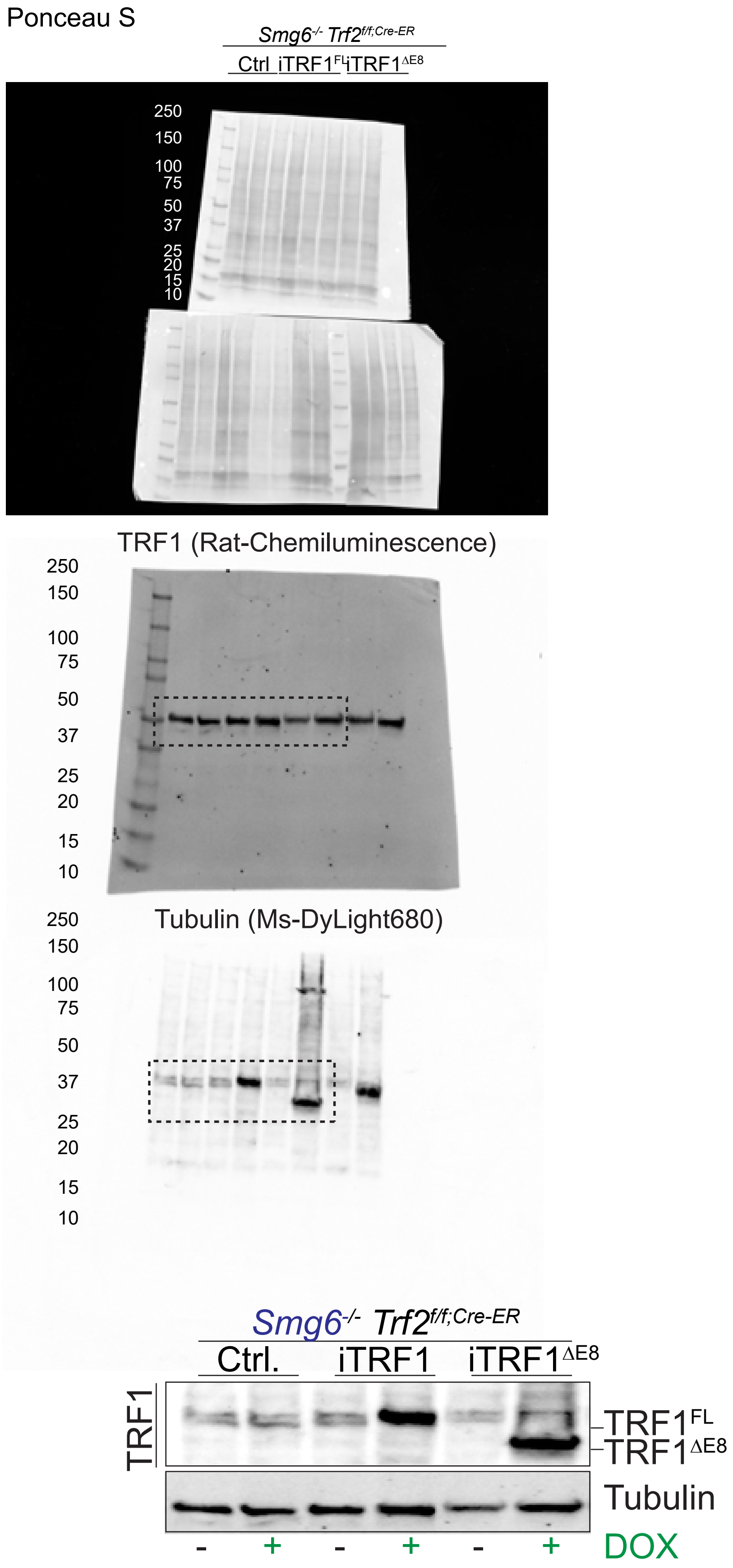

Figure 5d Source Data

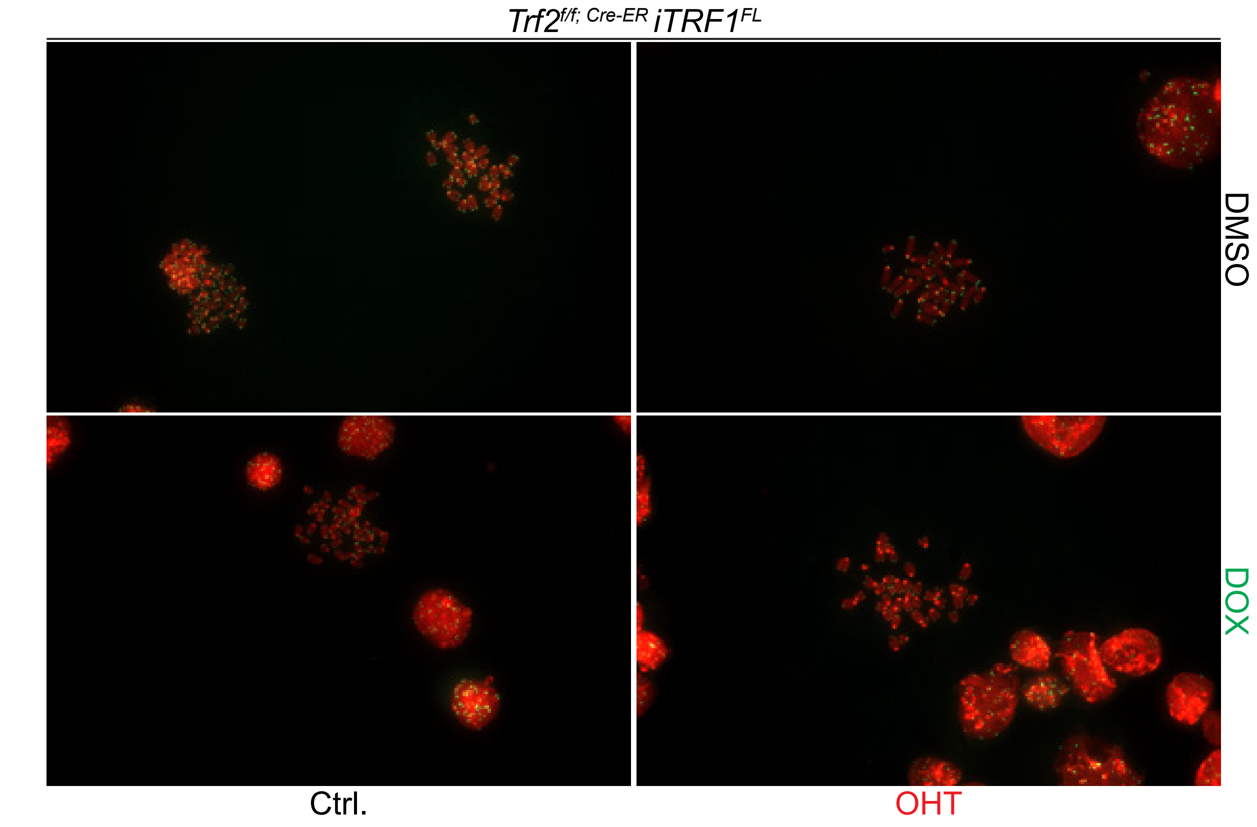


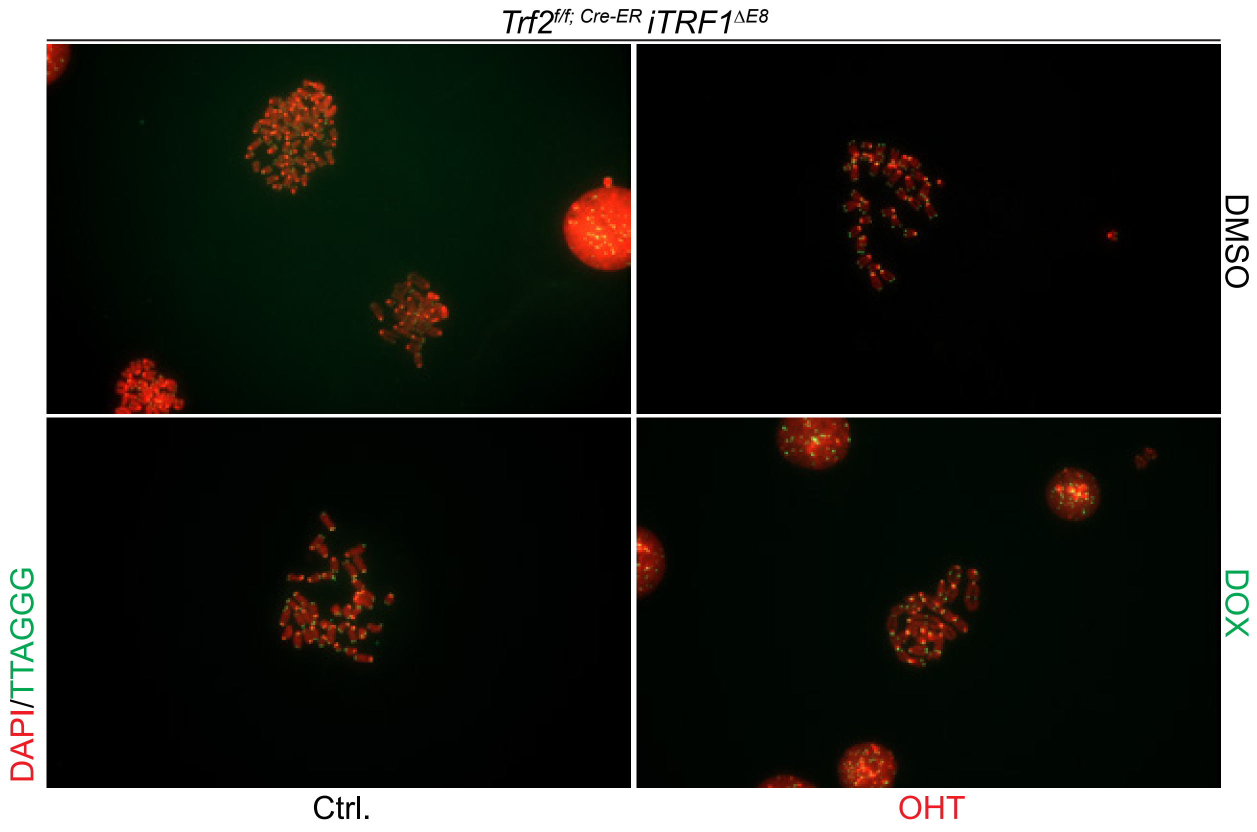

Figure 5e Source Data

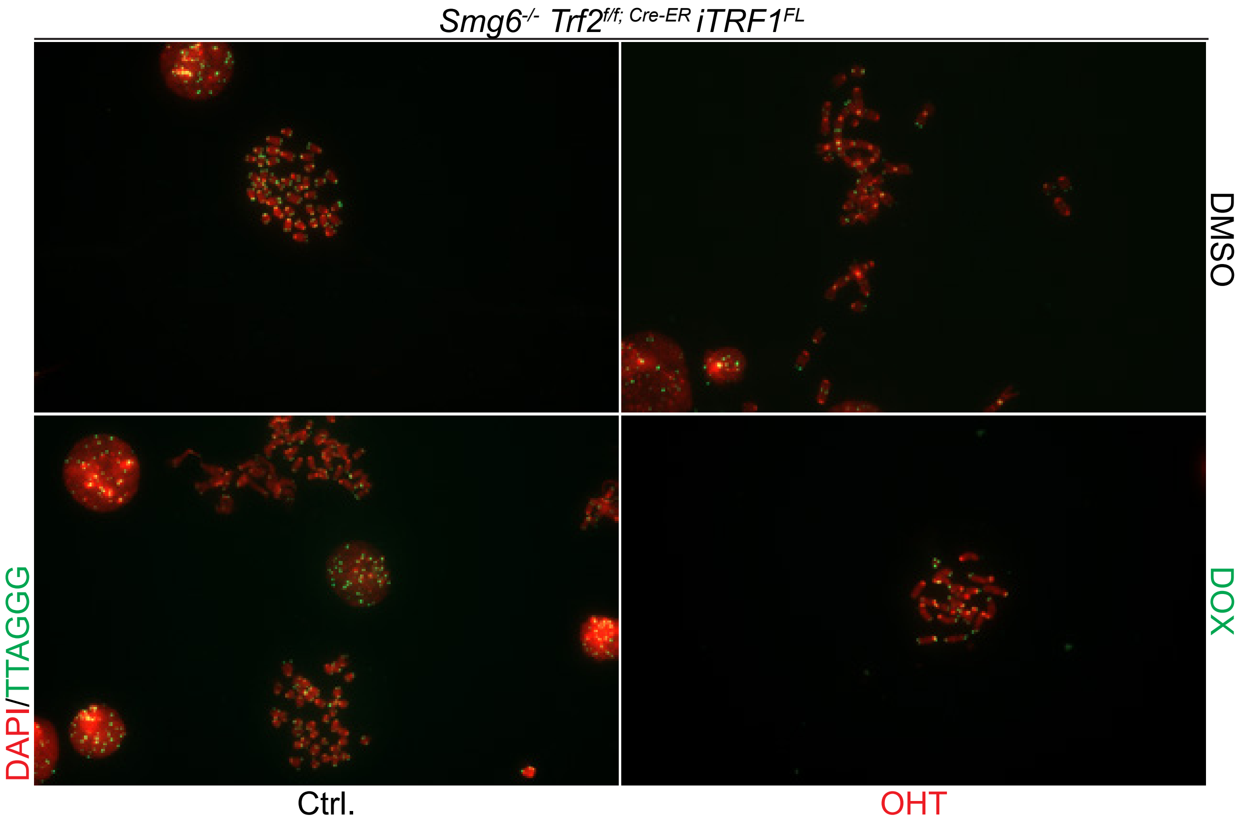

Figure 5f Source Data

PonceauS

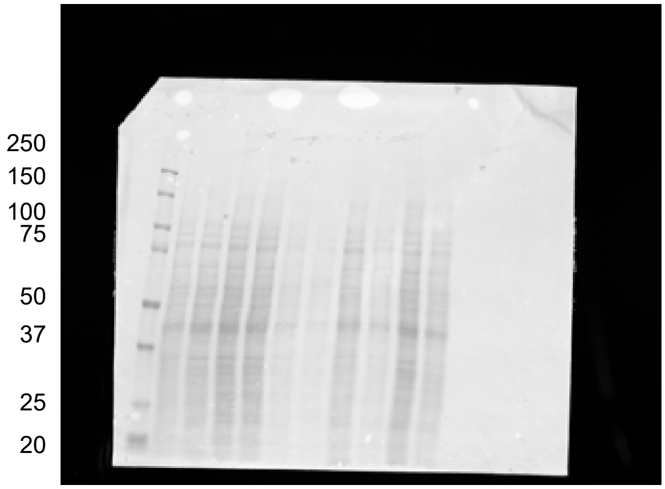


TRF1 (rat)

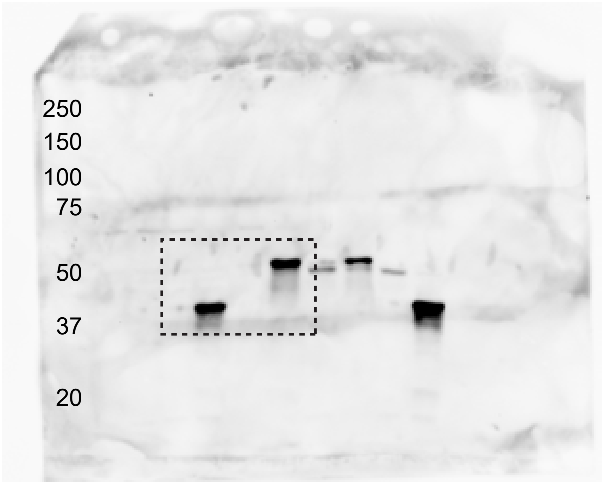


Actin (Ms)

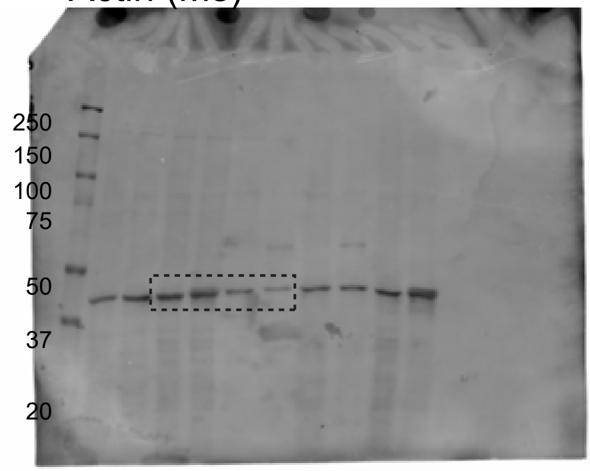


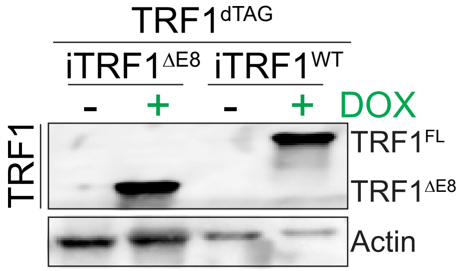

Figure 5g Source Data

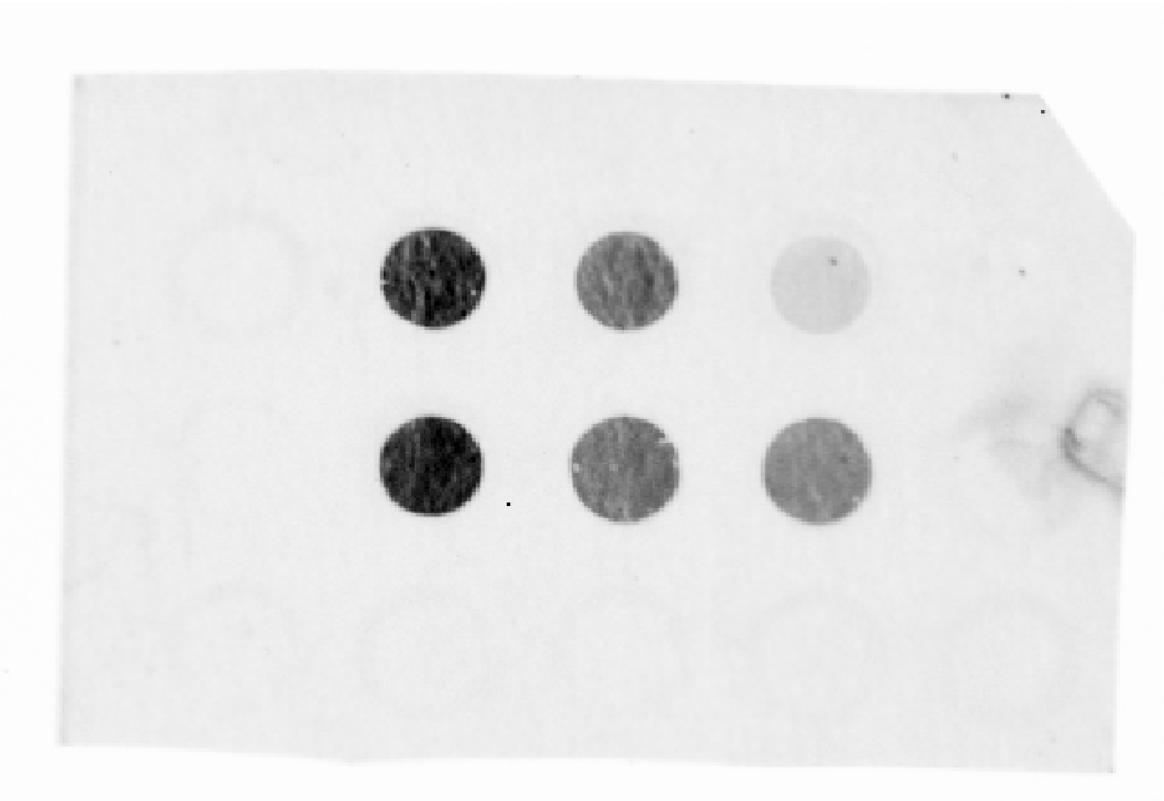

Extended Data Figure 1b Source Data

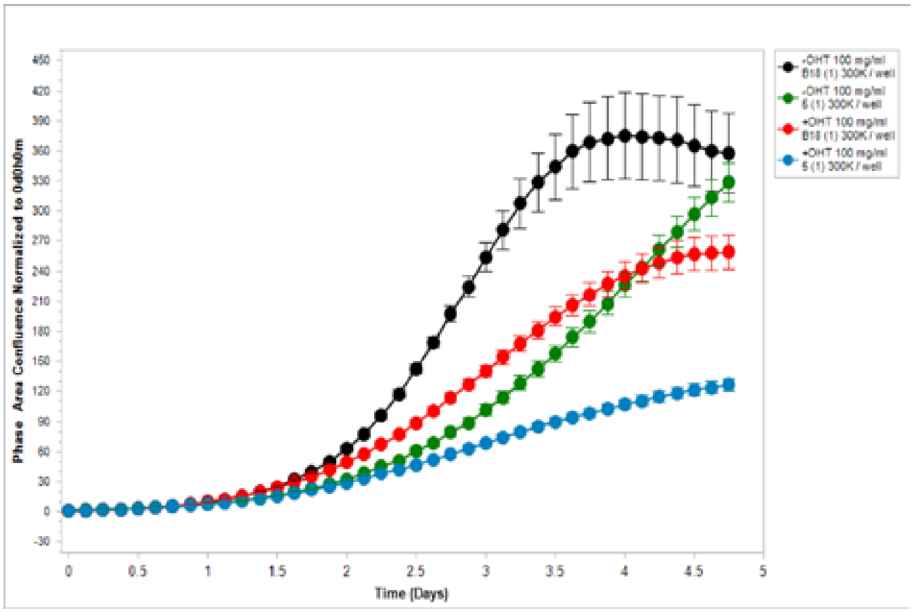


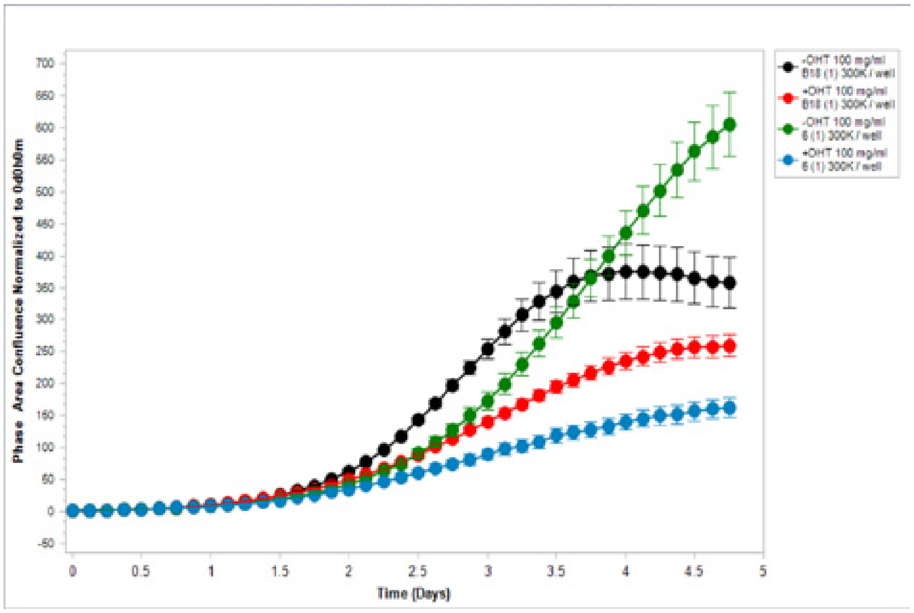


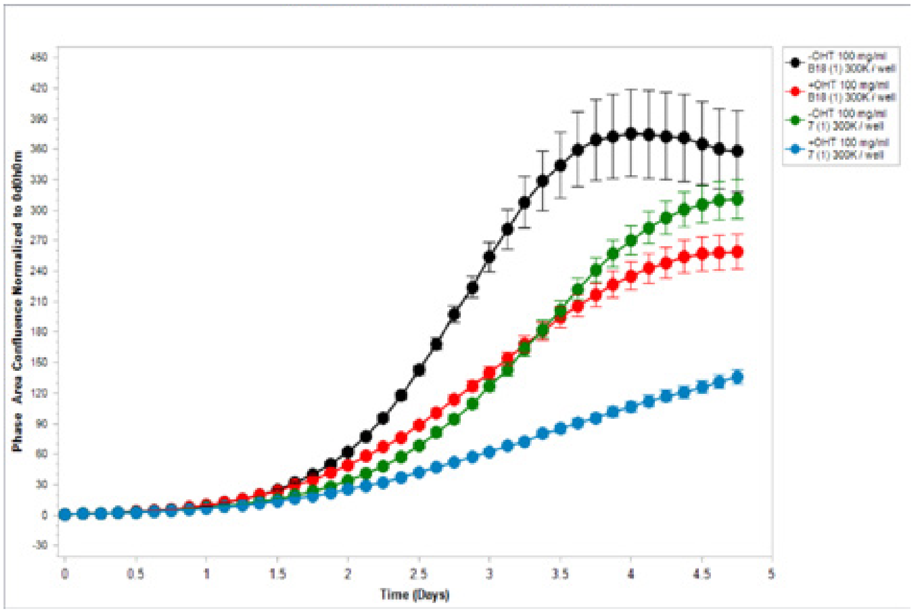

Extended Data Figure 2c Source Data

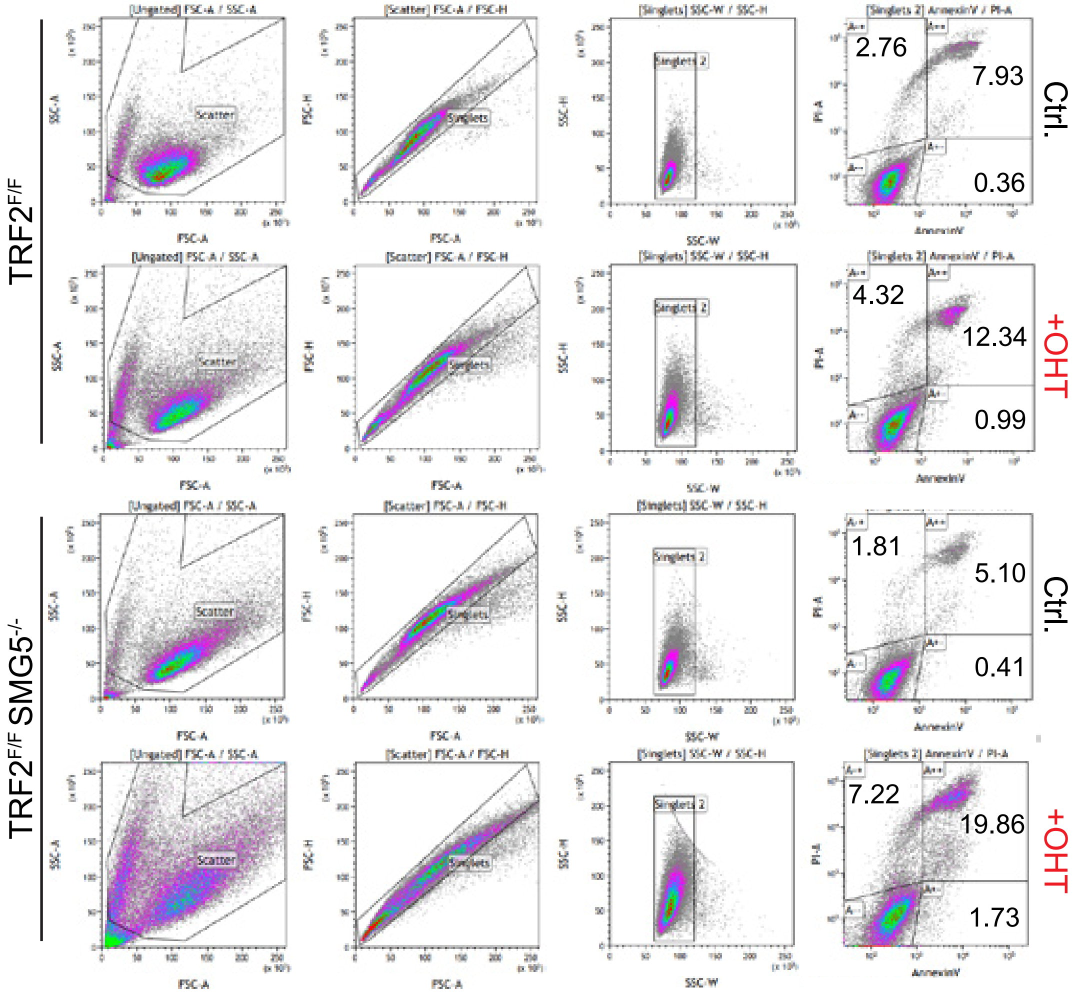


| Gate | Number | %Total | %Gated |
|---|---|---|---|
| All | 30,616 | 93.93 | 100.00 |
| A-- | 27,234 | 83.55 | 88.95 |
| A-+ | 844 | 2.59 | 2.76 |
| A+- | 111 | 0.34 | 0.36 |
| A++ | 2,427 | 7.45 | 7.93 |

8.29

| Gate | Number | %Total | %Gated |
|---|---|---|---|
| All | 31,130 | 83.88 | 100.00 |
| A-- | 25,638 | 69.08 | 82.36 |
| A-+ | 1,344 | 3.62 | 4.32 |
| A+- | 307 | 0.83 | 0.99 |
| A++ | 3,841 | 10.35 | 12.34 |

13.33

| Gate | Number | %Total | %Gated |
|---|---|---|---|
| All | 31,219 | 84.11 | 100.00 |
| A-- | 28,932 | 77.95 | 92.67 |
| A-+ | 565 | 1.52 | 1.81 |
| A+- | 129 | 0.35 | 0.41 |
| A++ | 1,593 | 4.29 | 5.10 |

5.51

| Gate | Number | %Total | %Gated |
|---|---|---|---|
| All | 34,352 | 69.27 | 100.00 |
| A-- | 24,454 | 49.31 | 71.19 |
| A-+ | 2,481 | 5.00 | 7.22 |
| A+- | 594 | 1.20 | 1.73 |
| A++ | 6,823 | 13.76 | 19.86 |

21.59

Extended Data Figure 2c-d Source Data cont.

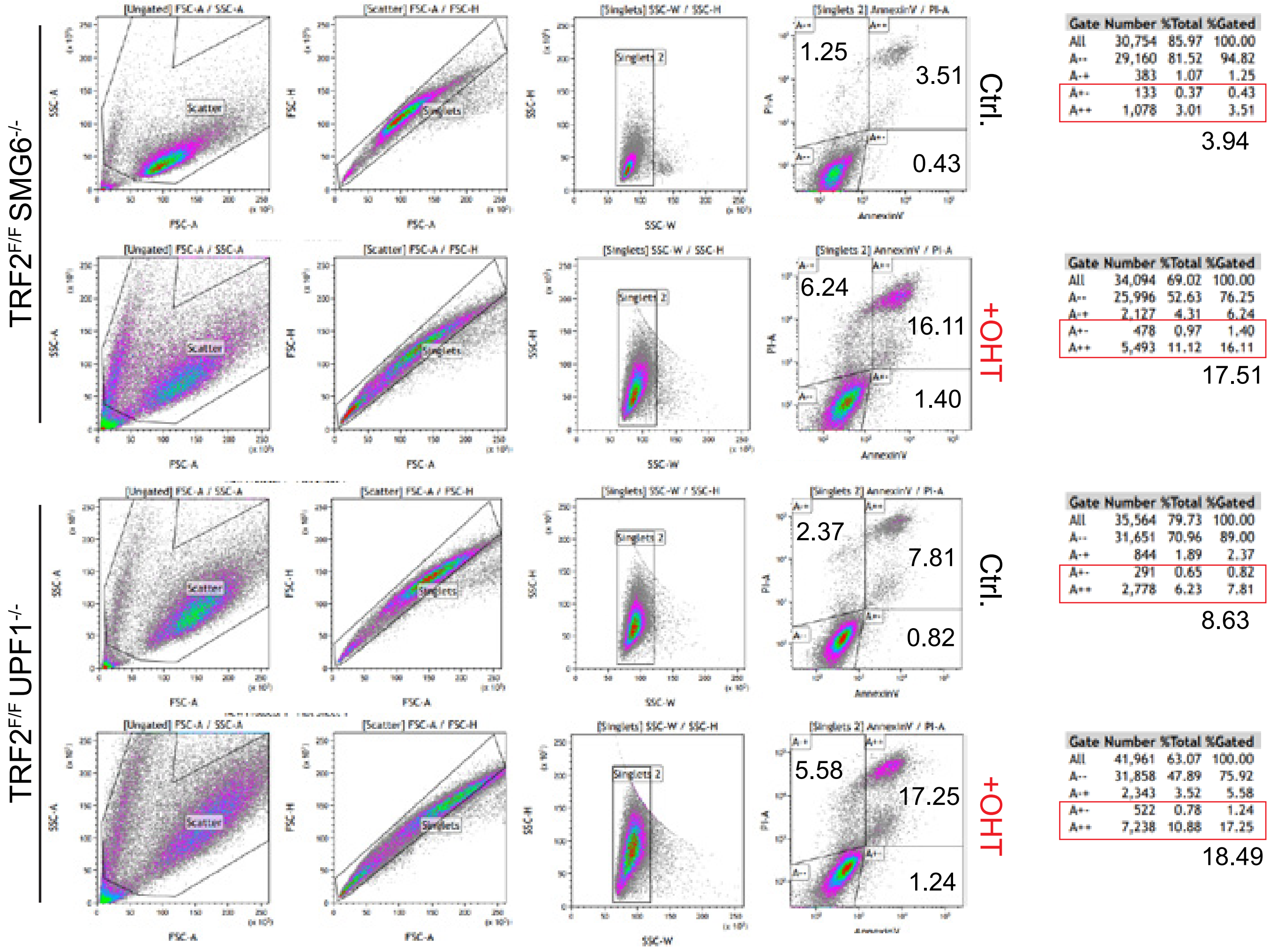


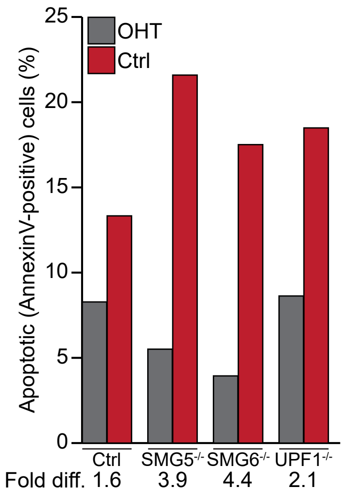

Extended Data Figure 2e Source Data cont. - *Trf2*$^{f/f; Cre-ER}$

## BD FACSDiva 9.0

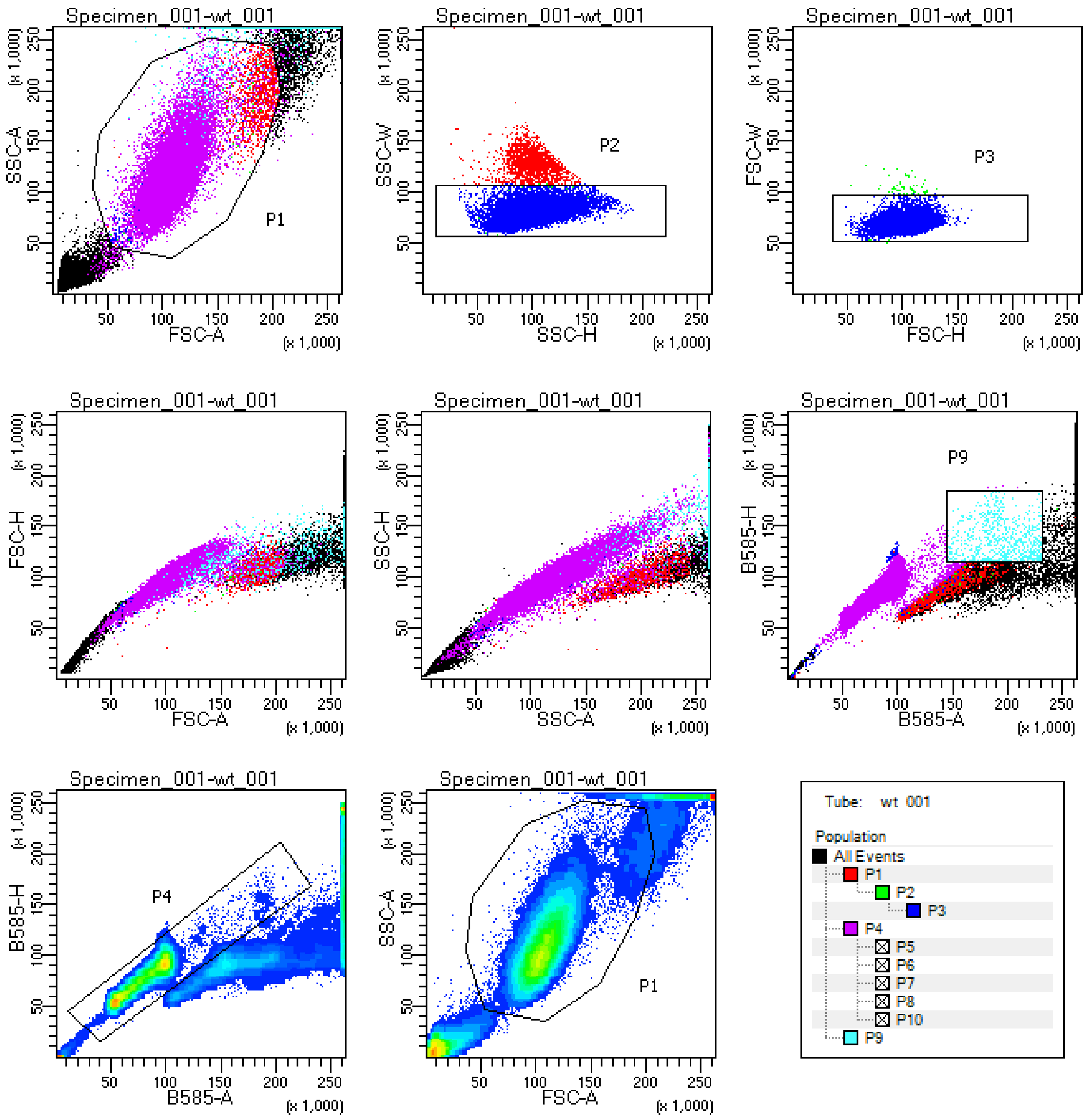


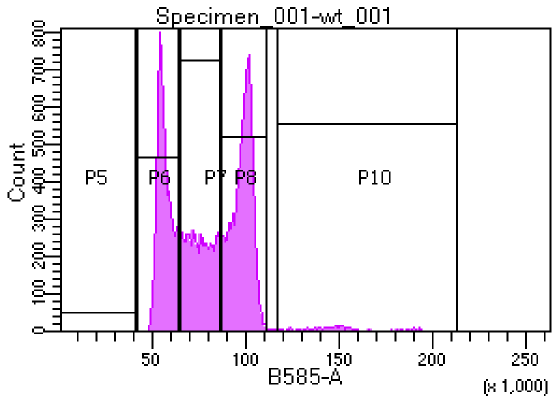


Specimen Name: Specimen_001
Tube Name: wt_001
Record Date: Sep 25, 2025 11:53:34 AM

| Population | #Events | %Parent | %Total | B585-A Median |
|---|---|---|---|---|
| All Events | 44,048 | #### | 100.0 | 88,743 |
| P1 | 21,920 | 49.8 | 49.8 | 82,277 |
| P2 | 20,251 | 92.4 | 46.0 | 79,094 |
| P3 | 20,178 | 99.6 | 45.8 | 78,987 |
| P4 | 20,775 | 47.2 | 47.2 | 80,010 |
| P5 | 41 | 0.2 | 0.1 | 34,405 |
| P6 | 6,200 | 29.8 | 14.1 | 53,881 |
| P7 | 5,108 | 24.6 | 11.6 | 74,124 |
| P8 | 8,029 | 38.6 | 18.2 | 97,454 |
| P10 | 780 | 3.8 | 1.8 | 149,590 |
| P9 | 734 | 1.7 | 1.7 | 193,155 |

Extended Data Figure 2e Source Data cont. - *Trf2*$^{-/-;\ Cre\text{-}ER}$ *Smg5*$^{-/-}$

## BD FACSDiva 9.0

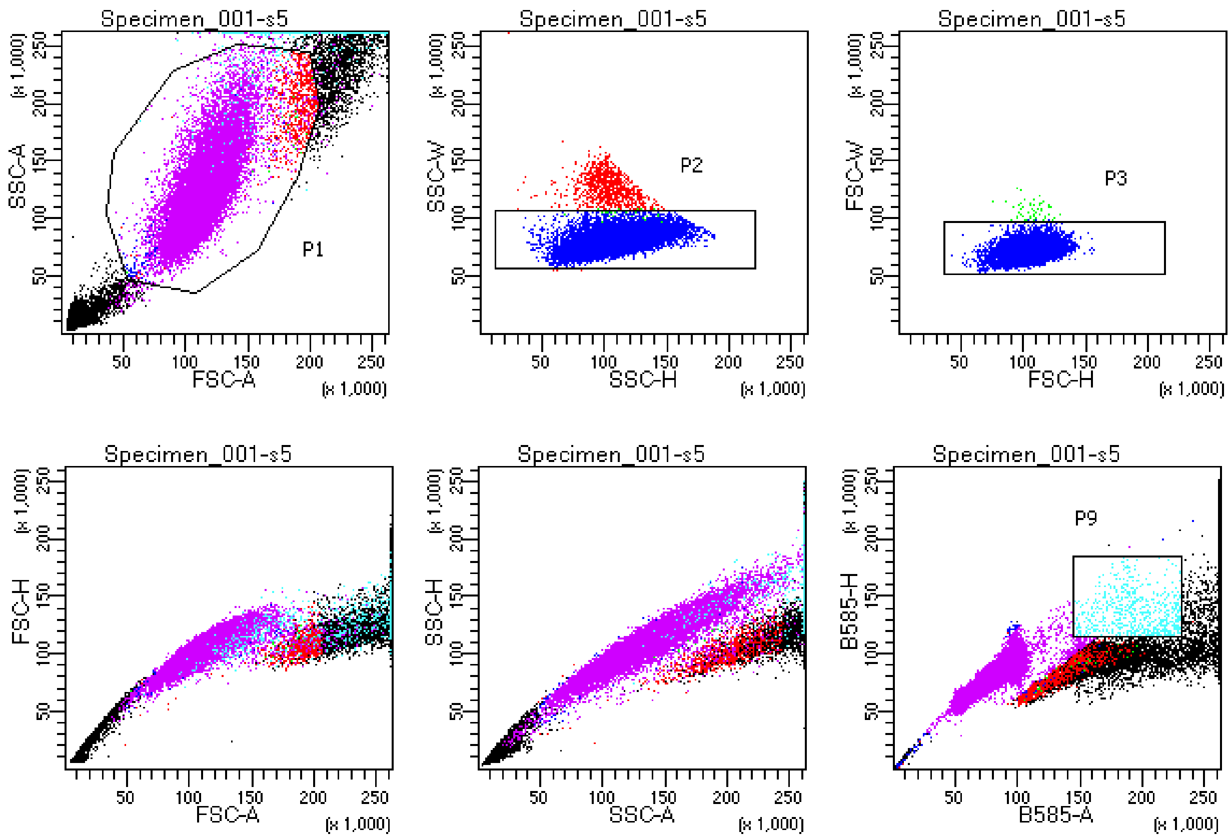


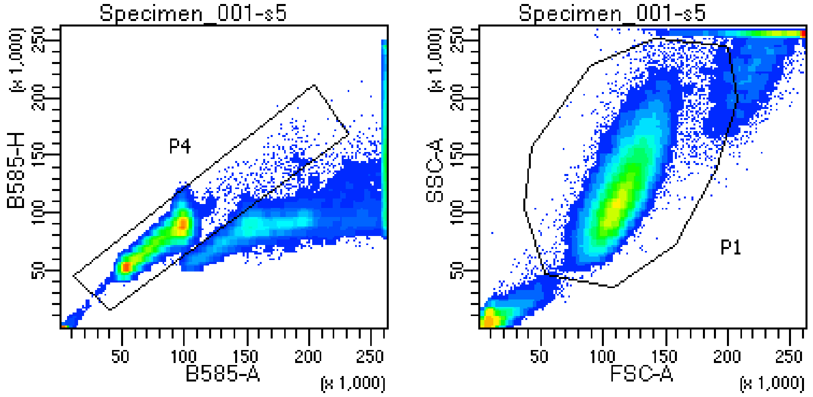


Tube: s5

Population
- All Events
  - P1
    - P2
      - P3
  - P4
    - P5
    - P6
    - P7
    - P8
    - P10
  - P9

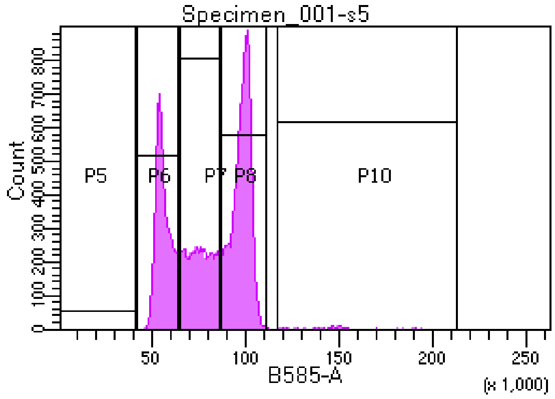


Specimen Name: Specimen_001
Tube Name: s5
Record Date: Sep 25, 2025 11:57:24 AM

| Population | #Events | %Parent | %Total | B585-A Median |
|---|---|---|---|---|
| All Events | 38,930 | #### | 100.0 | 93,499 |
| P1 | 21,118 | 54.2 | 54.2 | 83,626 |
| P2 | 20,170 | 95.5 | 51.8 | 81,688 |
| P3 | 20,111 | 99.7 | 51.7 | 81,573 |
| P4 | 20,597 | 52.9 | 52.9 | 82,766 |
| P5 | 30 | 0.1 | 0.1 | 32,519 |
| P6 | 5,744 | 27.9 | 14.8 | 53,322 |
| P7 | 4,773 | 23.2 | 12.3 | 74,167 |
| P8 | 8,945 | 43.4 | 23.0 | 97,041 |
| P10 | 581 | 2.8 | 1.5 | 150,061 |
| P9 | 652 | 1.7 | 1.7 | 195,365 |

Extended Data Figure 2e Source Data cont. - *Trf2*$^{f/f;\ Cre\text{-}ER}$ *Smg6*$^{-/-}$

## BD FACSDiva 9.0

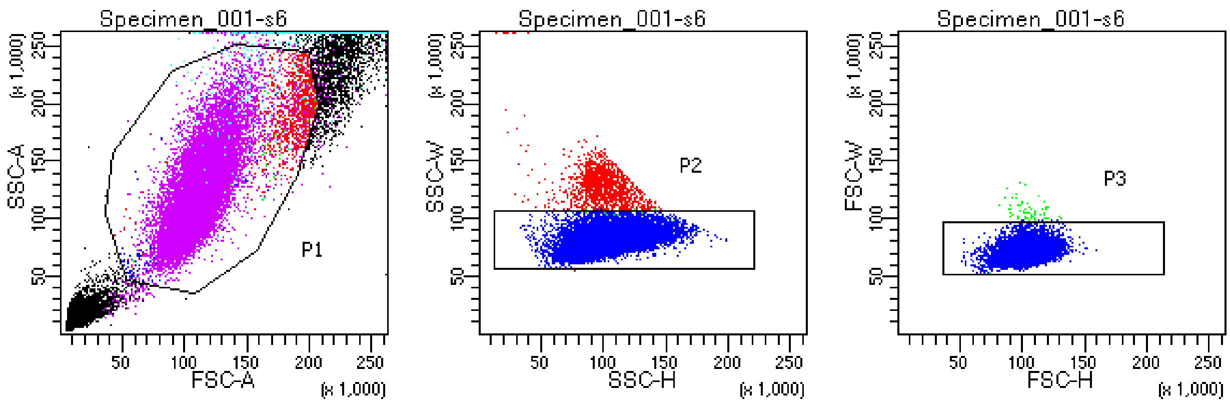


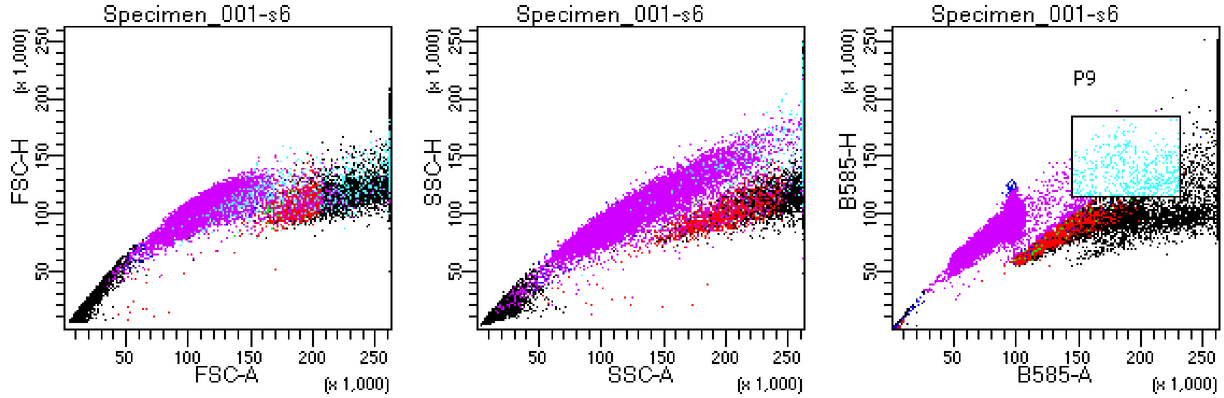


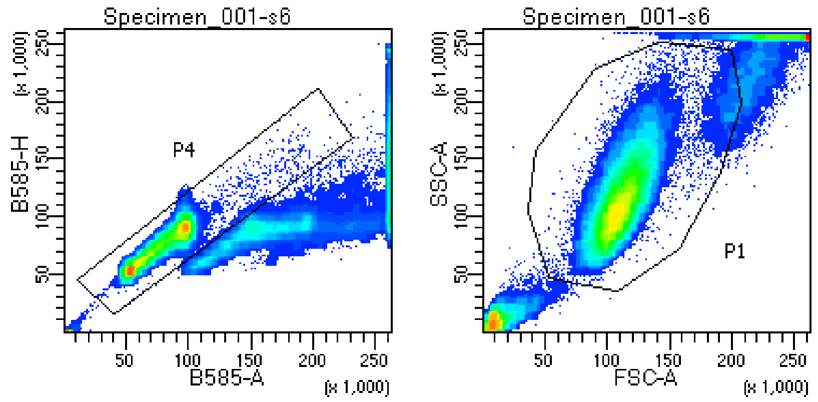


Tube: s6

Population
- All Events
  - P1
    - P2
      - P3
  - P4
    - P5
    - P6
    - P7
    - P8
    - P10
  - P9

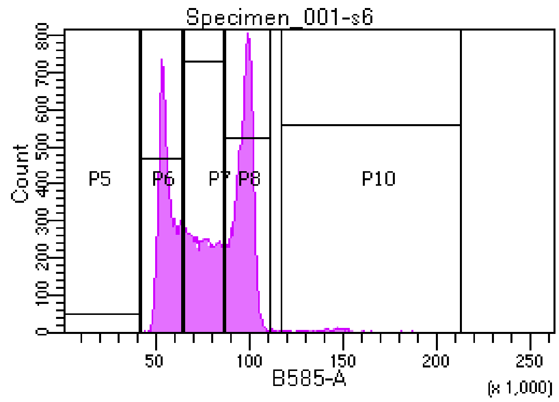


Specimen Name: Specimen_001
Tube Name: s6
Record Date: Sep 25, 2025 11:59:23 AM

| Population | #Events | %Parent | %Total | B585-A Median |
|---|---|---|---|---|
| All Events | 36,472 | #### | 100.0 | 88,735 |
| P1 | 21,450 | 58.8 | 58.8 | 80,548 |
| P2 | 20,042 | 93.4 | 55.0 | 77,944 |
| P3 | 19,977 | 99.7 | 54.8 | 77,805 |
| P4 | 20,605 | 56.5 | 56.5 | 78,875 |
| P5 | 29 | 0.1 | 0.1 | 33,163 |
| P6 | 6,278 | 30.5 | 17.2 | 53,157 |
| P7 | 5,186 | 25.2 | 14.2 | 74,025 |
| P8 | 7,929 | 38.5 | 21.7 | 95,930 |
| P10 | 596 | 2.9 | 1.6 | 146,604 |
| P9 | 577 | 1.6 | 1.6 | 195,056 |

Extended Data Figure 2e Source Data cont. - *Trf2*$^{-/-; Cre\text{-}ER}$ *Upf1*$^{HM}$

BD FACSDiva 9.0

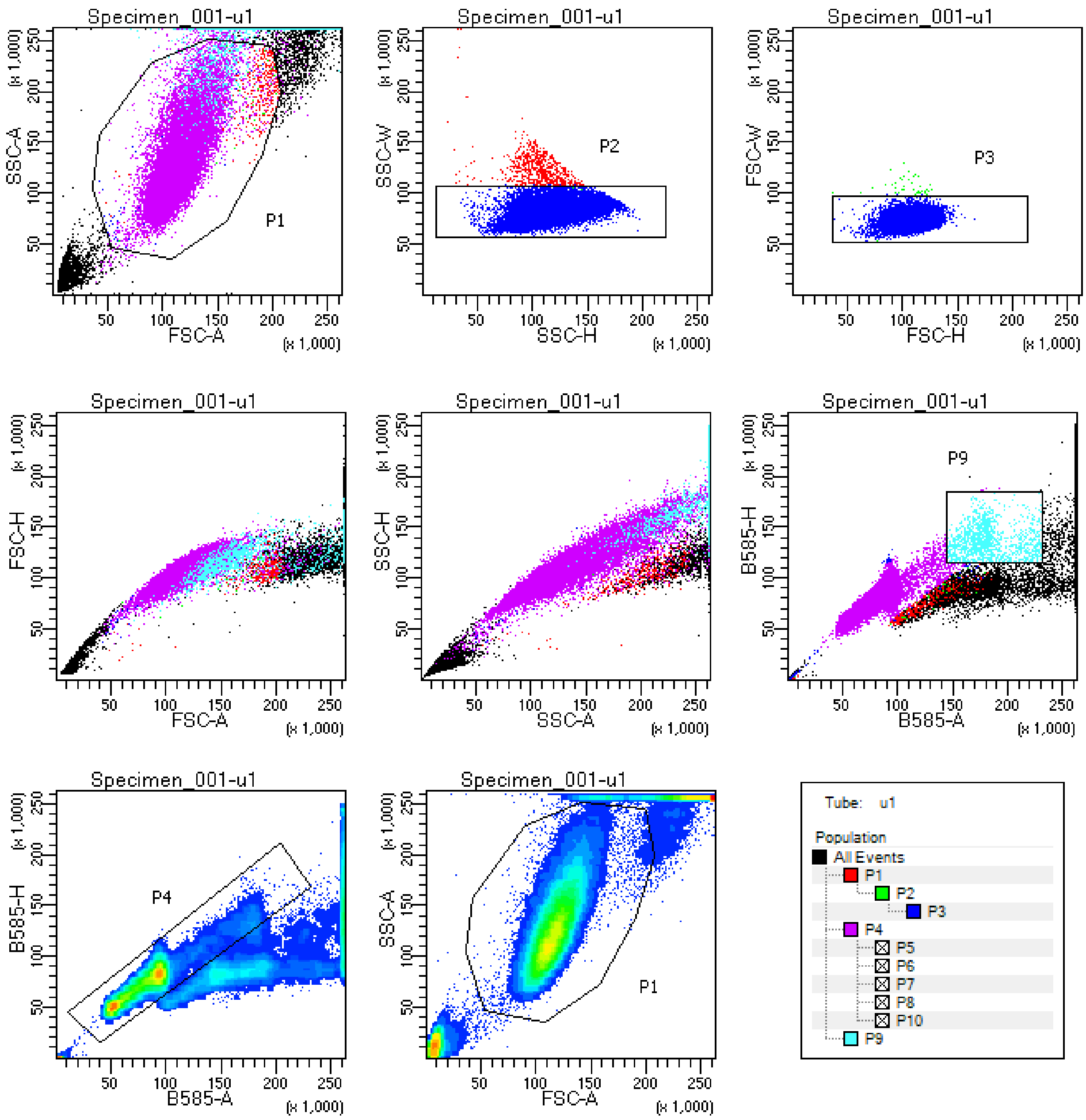


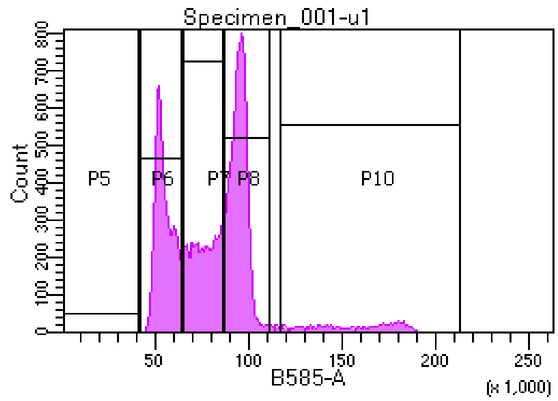


Specimen Name: Specimen_001
Tube Name: u1
Record Date: Sep 25, 2025 12:01:38 PM

| Population | #Events | %Parent | %Total | B585-A Median |
|---|---|---|---|---|
| All Events | 35,978 | #### | 100.0 | 88,685 |
| P1 | 20,875 | 58.0 | 58.0 | 81,254 |
| P2 | 20,245 | 97.0 | 56.3 | 80,301 |
| P3 | 20,203 | 99.8 | 56.2 | 80,242 |
| P4 | 21,337 | 59.3 | 59.3 | 82,307 |
| P5 | 12 | 0.1 | 0.0 | 35,443 |
| P6 | 6,171 | 28.9 | 17.2 | 51,825 |
| P7 | 5,017 | 23.5 | 13.9 | 74,840 |
| P8 | 7,949 | 37.3 | 22.1 | 93,344 |
| P10 | 1,462 | 6.9 | 4.1 | 157,173 |
| P9 | 1,337 | 3.7 | 3.7 | 178,836 |

Extended Data Figure 2e Source Data cont. - *Trf2*$^{-/-; Cre-ER}$

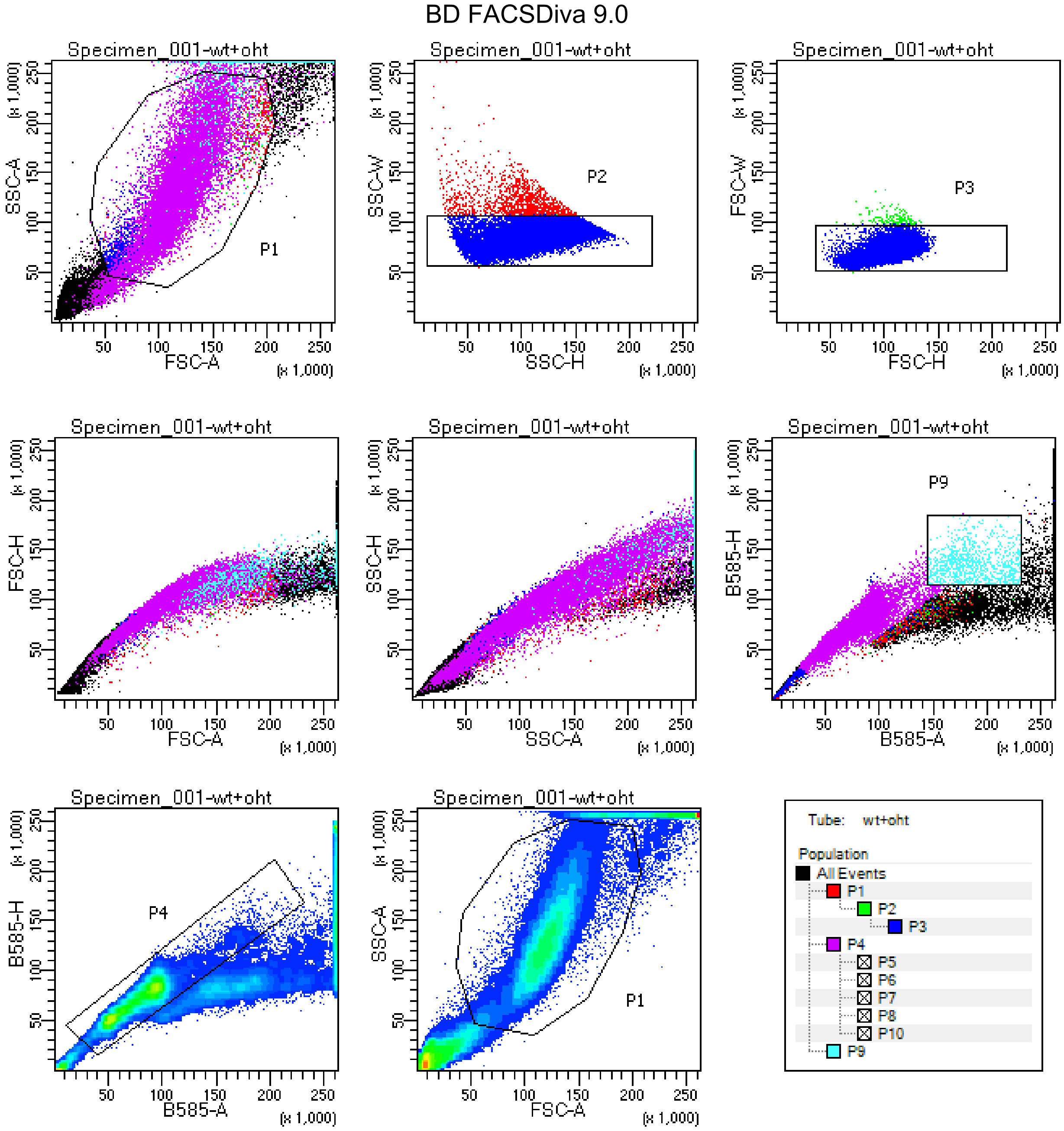


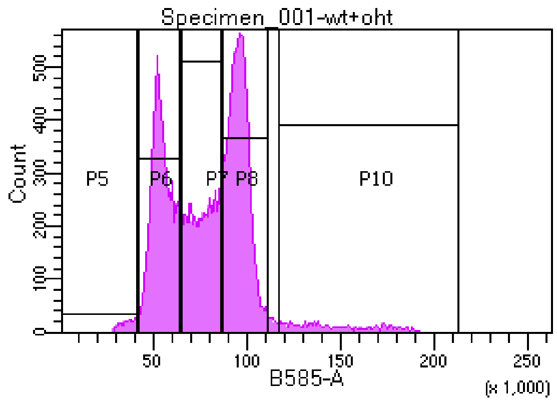


Specimen Name: Specimen_001
Tube Name: wt+oht
Record Date: Sep 25, 2025 12:07:35 PM

| Population | #Events | %Parent | %Total | B585-A Median |
|---|---|---|---|---|
| All Events | 62,840 | #### | 100.0 | 49,226 |
| P1 | 20,041 | 31.9 | 31.9 | 77,184 |
| P2 | 18,686 | 93.2 | 29.7 | 76,093 |
| P3 | 18,524 | 99.1 | 29.5 | 75,834 |
| P4 | 20,542 | 32.7 | 32.7 | 80,336 |
| P5 | 310 | 1.5 | 0.5 | 34,046 |
| P6 | 6,110 | 29.7 | 9.7 | 51,846 |
| P7 | 4,821 | 23.5 | 7.7 | 74,976 |
| P8 | 7,362 | 35.8 | 11.7 | 94,136 |
| P10 | 1,129 | 5.5 | 1.8 | 148,454 |
| P9 | 1,002 | 1.6 | 1.6 | 183,747 |

Extended Data Figure 2e Source Data cont. - *Trf2*$^{-/-;\ Cre\text{-}ER}$ *Smg5*$^{-/-}$

BD FACSDiva 9.0

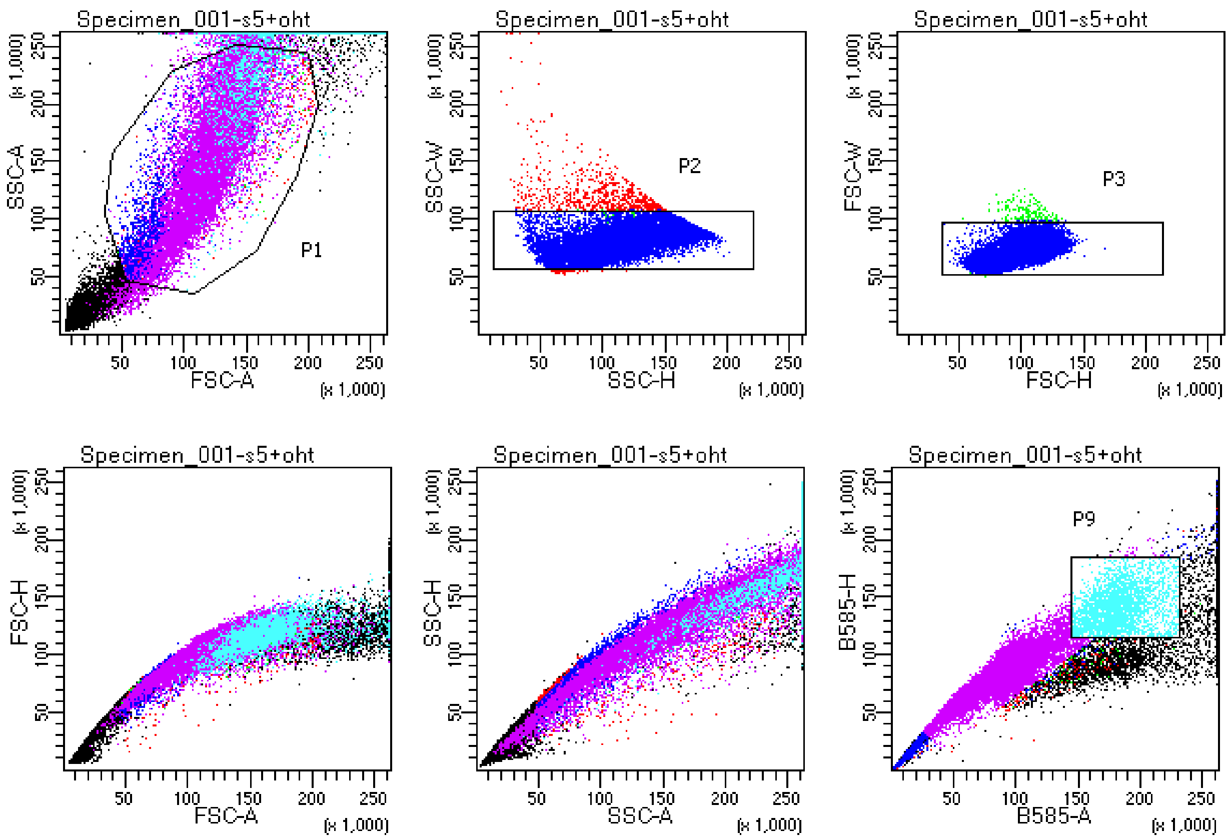


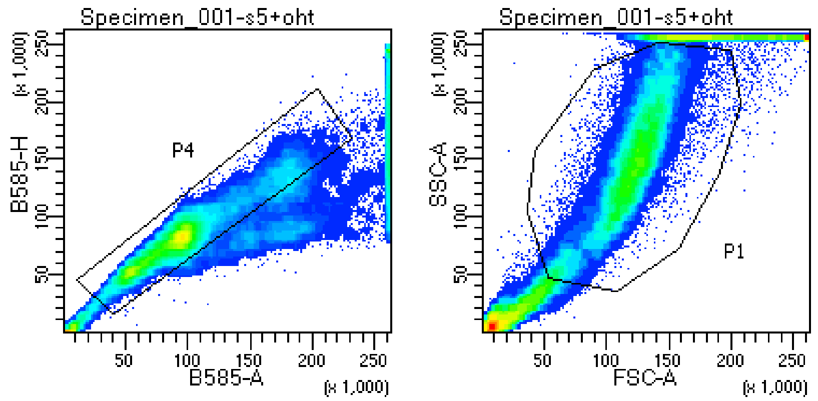


Tube: s5+oht

Population
- All Events
  - P1
    - P2
      - P3
  - P4
    - P5
    - P6
    - P7
    - P8
    - P10
  - P9

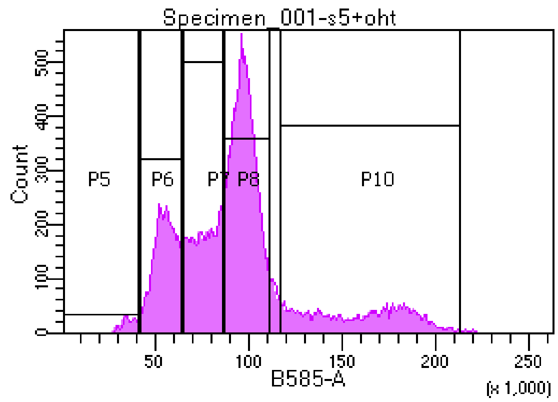


Specimen Name: Specimen_001
Tube Name: s5+oht
Record Date: Sep 25, 2025 12:13:30 PM

| Population | #Events | %Parent | %Total | B585-A Median |
|---|---|---|---|---|
| All Events | 43,959 | #### | 100.0 | 70,537 |
| P1 | 19,570 | 44.5 | 44.5 | 81,828 |
| P2 | 18,740 | 95.8 | 42.6 | 81,727 |
| P3 | 18,566 | 99.1 | 42.2 | 81,481 |
| P4 | 20,415 | 46.4 | 46.4 | 91,002 |
| P5 | 345 | 1.7 | 0.8 | 33,821 |
| P6 | 3,667 | 18.0 | 8.3 | 53,227 |
| P7 | 3,926 | 19.2 | 8.9 | 75,069 |
| P8 | 8,106 | 39.7 | 18.4 | 95,927 |
| P10 | 3,264 | 16.0 | 7.4 | 161,555 |
| P9 | 2,820 | 6.4 | 6.4 | 181,448 |

# Extended Data Figure 2e Source Data cont. - *Trf2$^{f/f; Cre-ER}$ Smg6$^{-/-}$*

BD FACSDiva 9.0

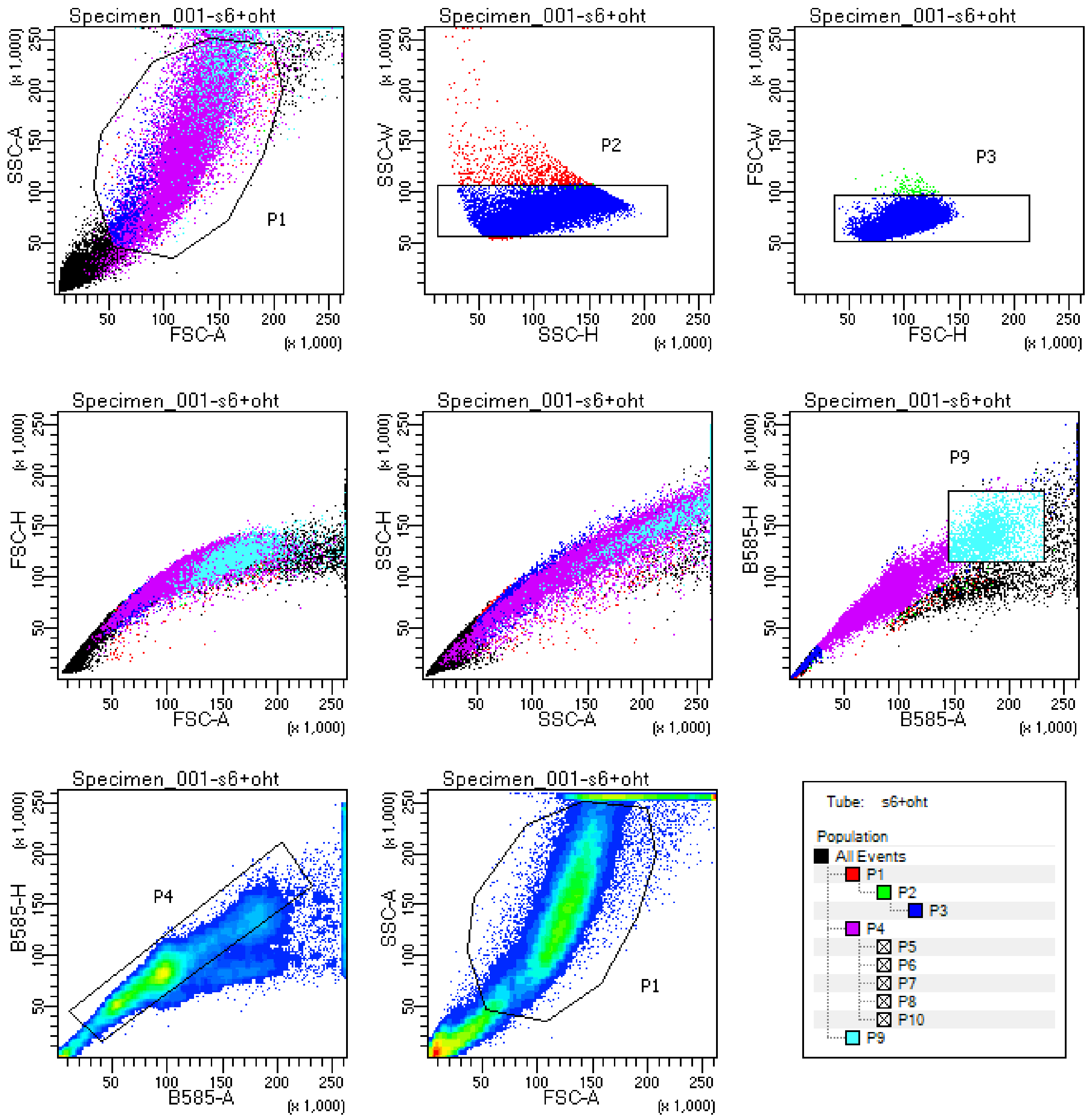


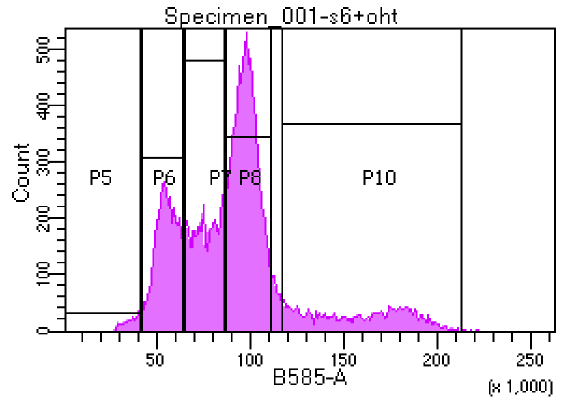


Specimen Name: Specimen_001
Tube Name: s6+oht
Record Date: Sep 25, 2025 12:15:54 PM

| Population | #Events | %Parent | %Total | B585-A Median |
|---|---|---|---|---|
| All Events | 42,084 | #### | 100.0 | 60,078 |
| P1 | 19,725 | 46.9 | 46.9 | 81,458 |
| P2 | 18,858 | 95.6 | 44.8 | 81,524 |
| P3 | 18,745 | 99.4 | 44.5 | 81,408 |
| P4 | 20,063 | 47.7 | 47.7 | 90,322 |
| P5 | 327 | 1.6 | 0.8 | 34,458 |
| P6 | 3,915 | 19.5 | 9.3 | 53,083 |
| P7 | 3,869 | 19.3 | 9.2 | 74,801 |
| P8 | 8,069 | 40.2 | 19.2 | 96,144 |
| P10 | 2,806 | 14.0 | 6.7 | 160,357 |
| P9 | 2,342 | 5.6 | 5.6 | 180,615 |

## Extended Data Figure 2e Source Data cont. - *Trf2*$^{-/-; Cre-ER}$ *Upf1*$^{HM}$

### BD FACSDiva 9.0

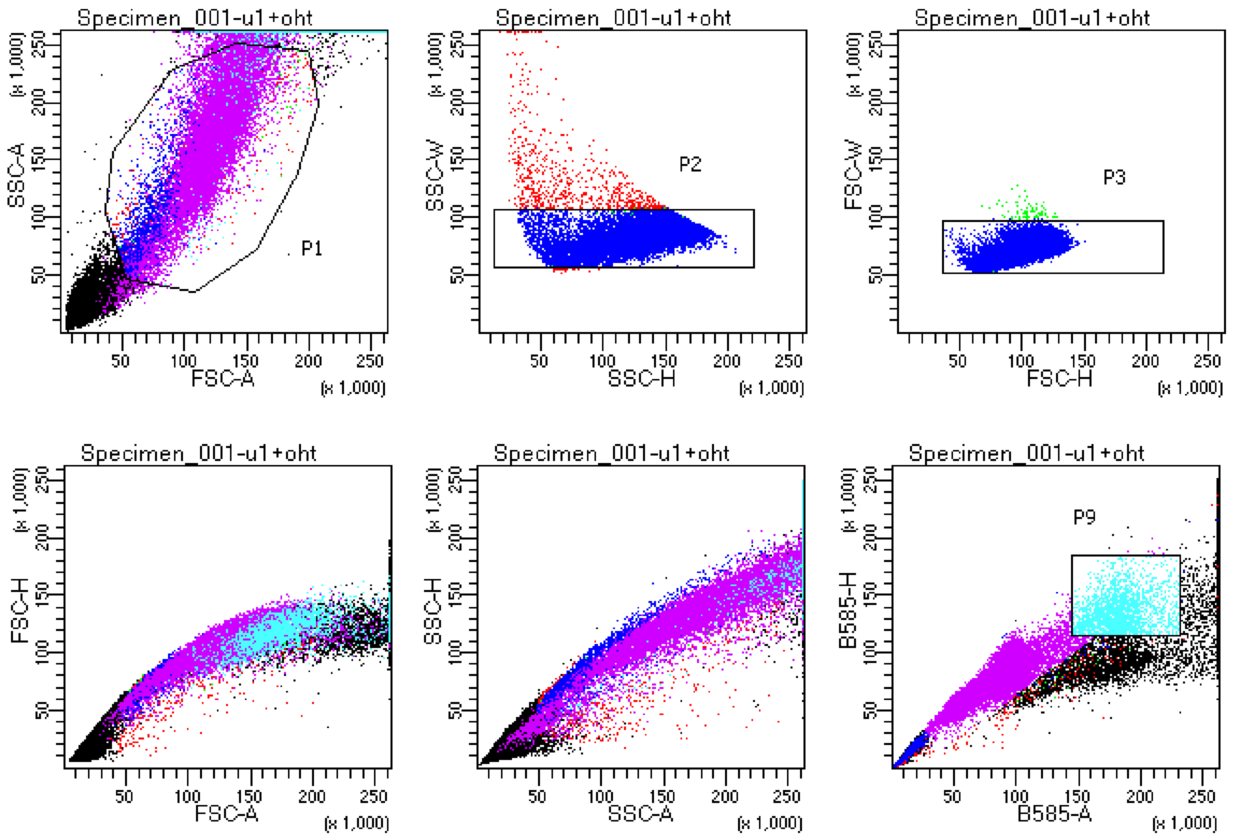


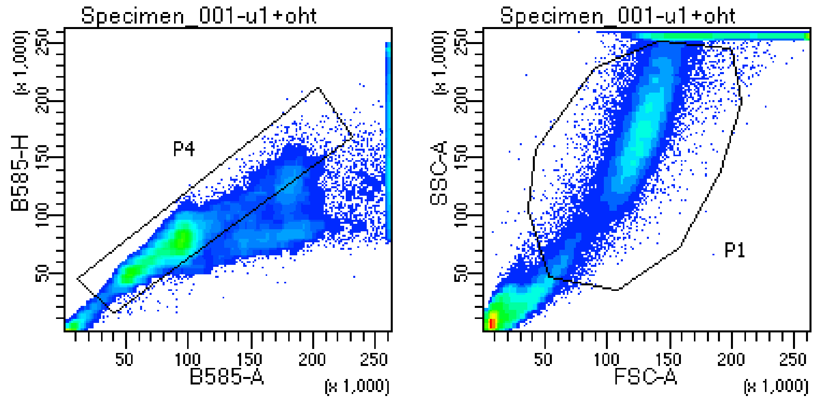


Tube: u1+oht

Population
- All Events
  - P1
    - P2
      - P3
  - P4
    - P5
    - P6
    - P7
    - P8
    - P10
  - P9

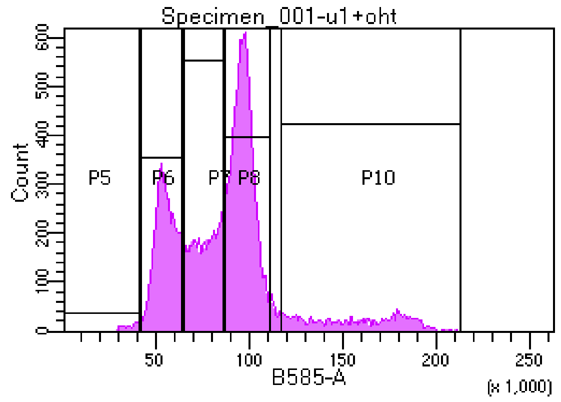


Specimen Name: Specimen_001
Tube Name: u1+oht
Record Date: Sep 25, 2025 12:23:22 PM

| Population | #Events | %Parent | %Total | B585-A Median |
|---|---|---|---|---|
| All Events | 72,524 | #### | 100.0 | 666 |
| P1 | 17,516 | 24.2 | 24.2 | 70,163 |
| P2 | 16,705 | 95.4 | 23.0 | 70,045 |
| P3 | 16,614 | 99.5 | 22.9 | 69,996 |
| P4 | 20,018 | 27.6 | 27.6 | 88,272 |
| P5 | 197 | 1.0 | 0.3 | 33,876 |
| P6 | 4,575 | 22.9 | 6.3 | 52,828 |
| P7 | 3,996 | 20.0 | 5.5 | 75,272 |
| P8 | 8,176 | 40.8 | 11.3 | 95,152 |
| P10 | 2,188 | 10.9 | 3.0 | 158,487 |
| P9 | 2,066 | 2.8 | 2.8 | 181,818 |

Extended Data Figure 3c Source Data

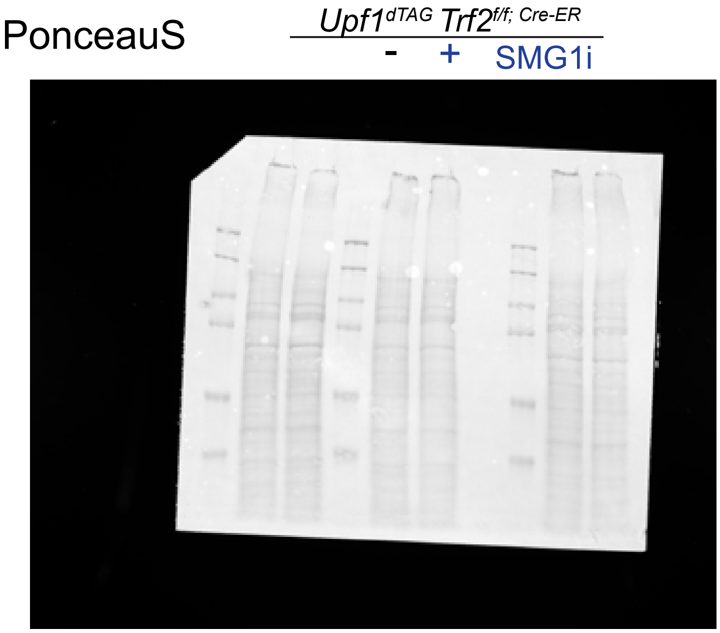


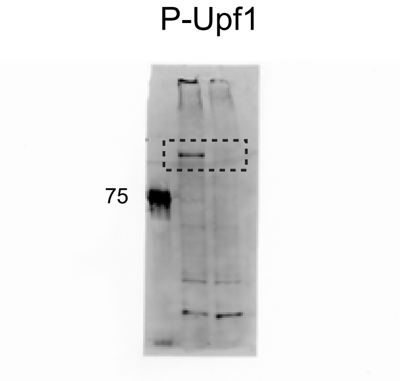


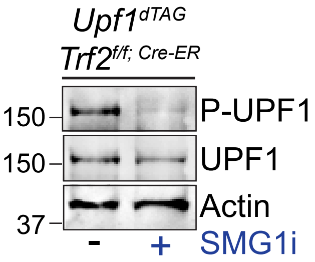


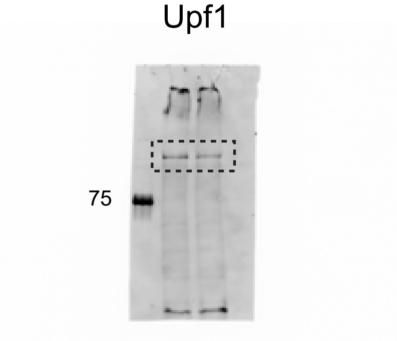


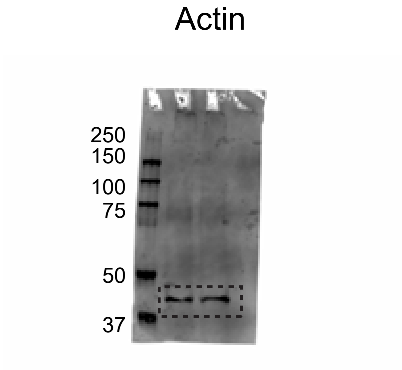

Extended Data Figure 4e Source Data

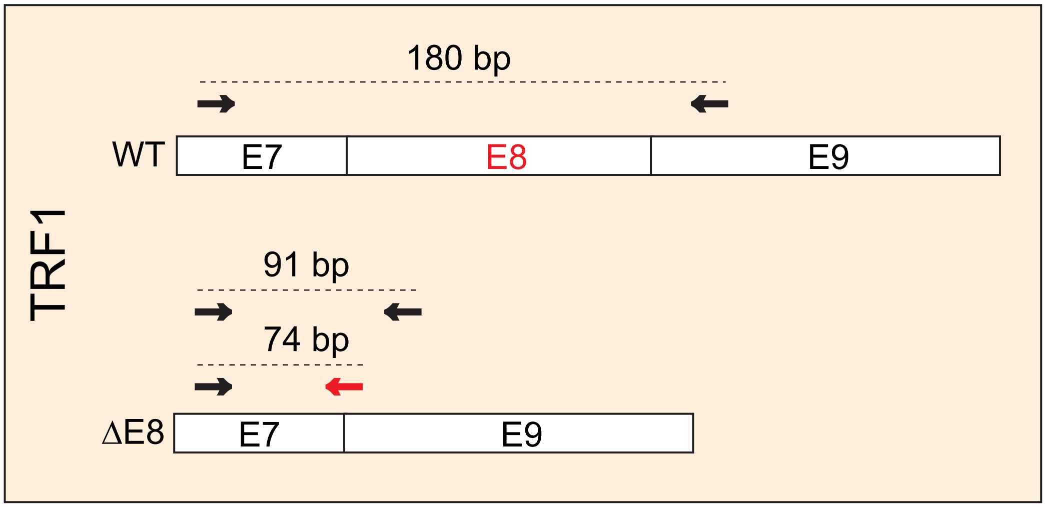


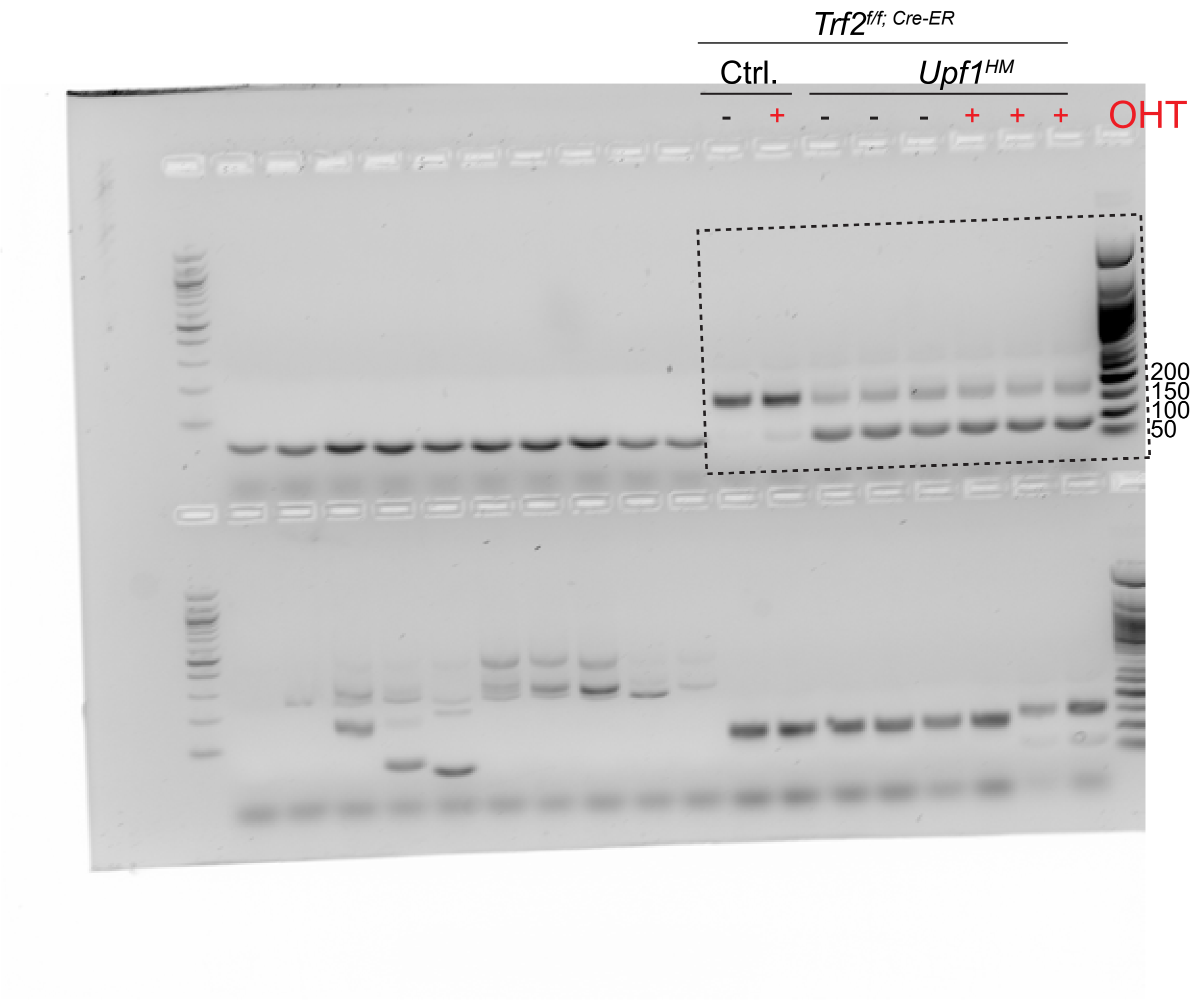

Extended Data Figure 4g Source Data

PonceauS

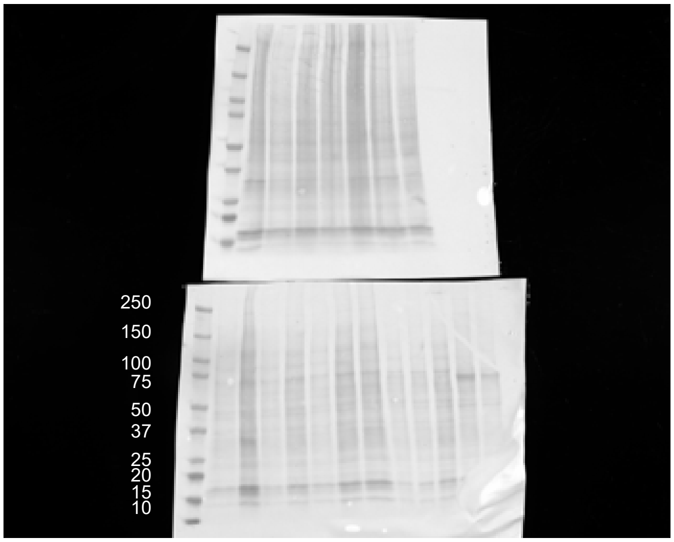


TRF1 (Rat-Chemiluminescence)

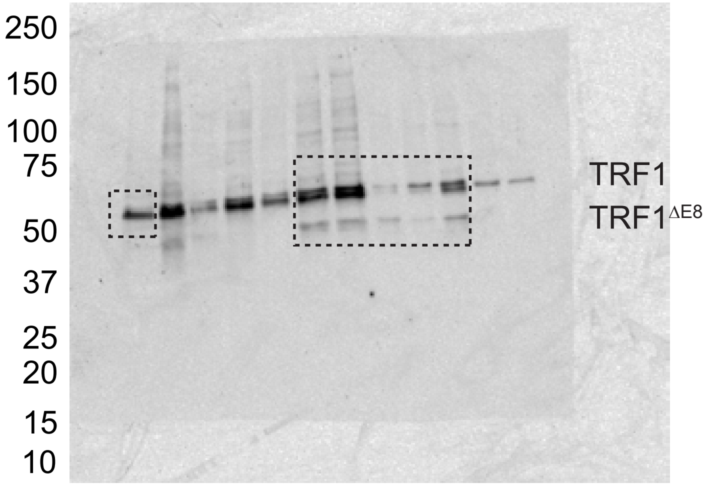


Tubulin (Ms-DyLight680)

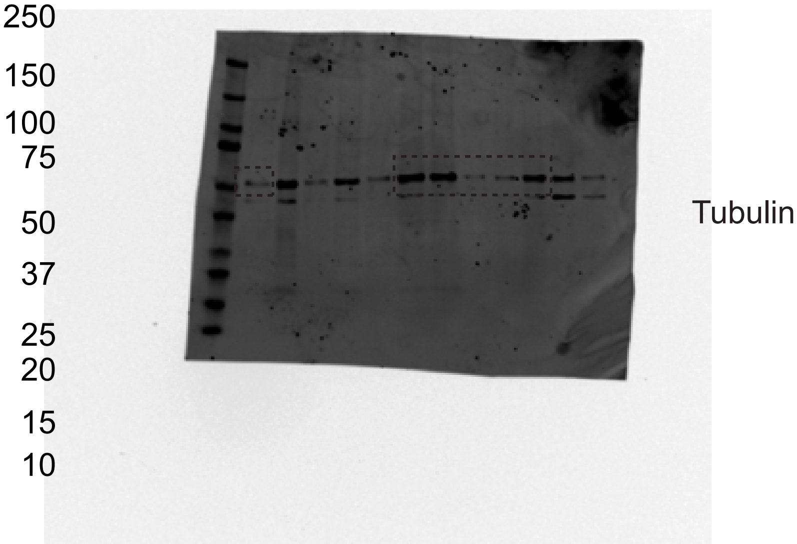


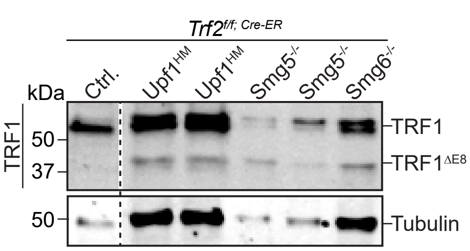

Extended Data Figure 4h Source Data

## PonceauS

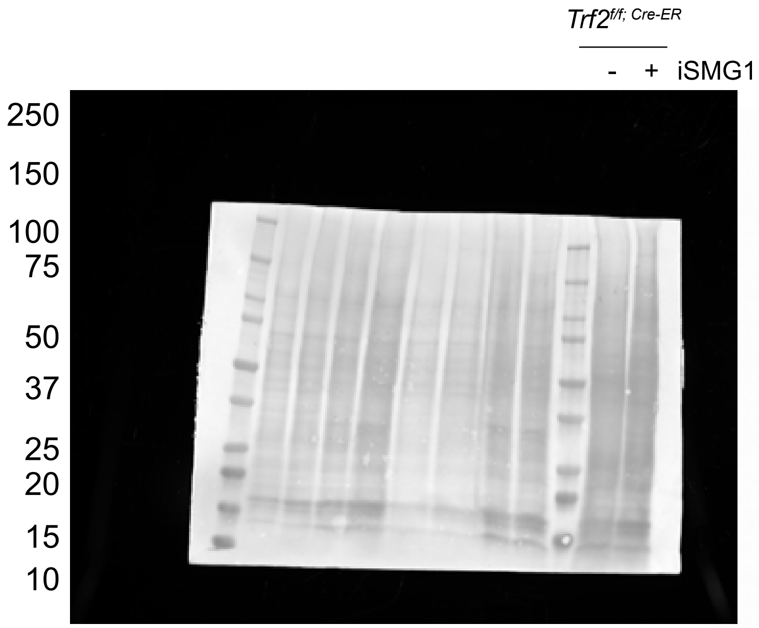


## Tubulin (Ms-DyLight680)

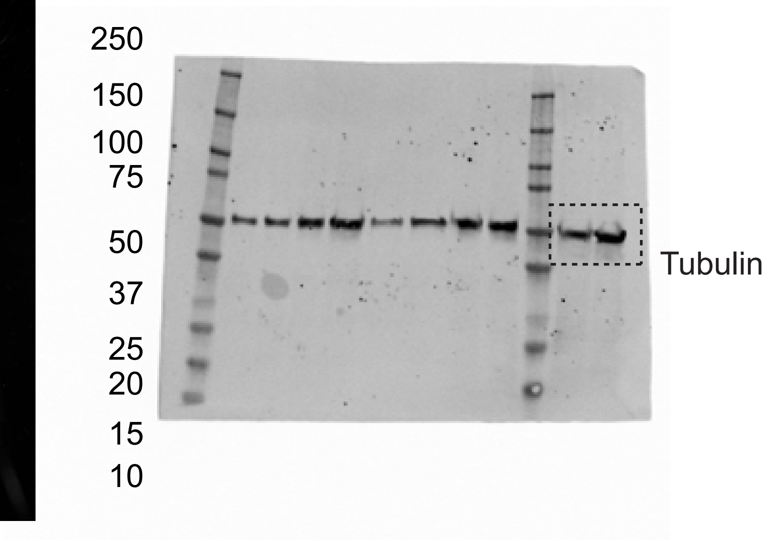


## TRF1 (Rat-Chemiluminescence)

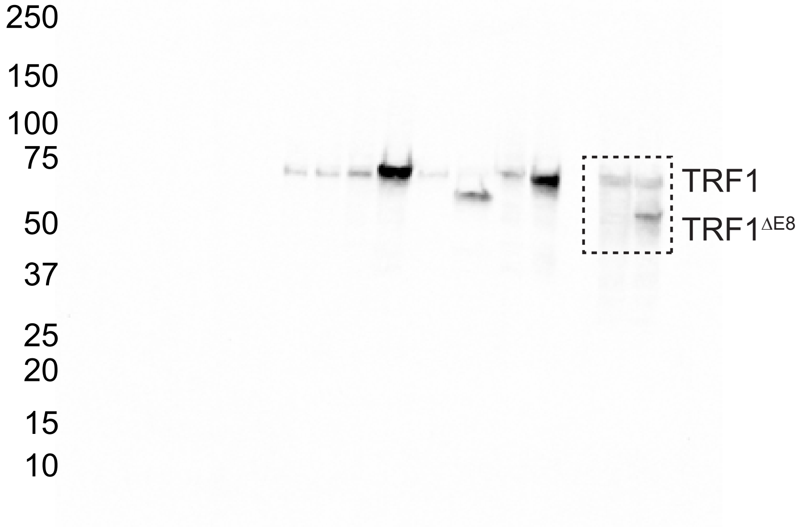


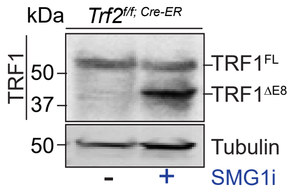

Extended Data Figure 4j Source Data

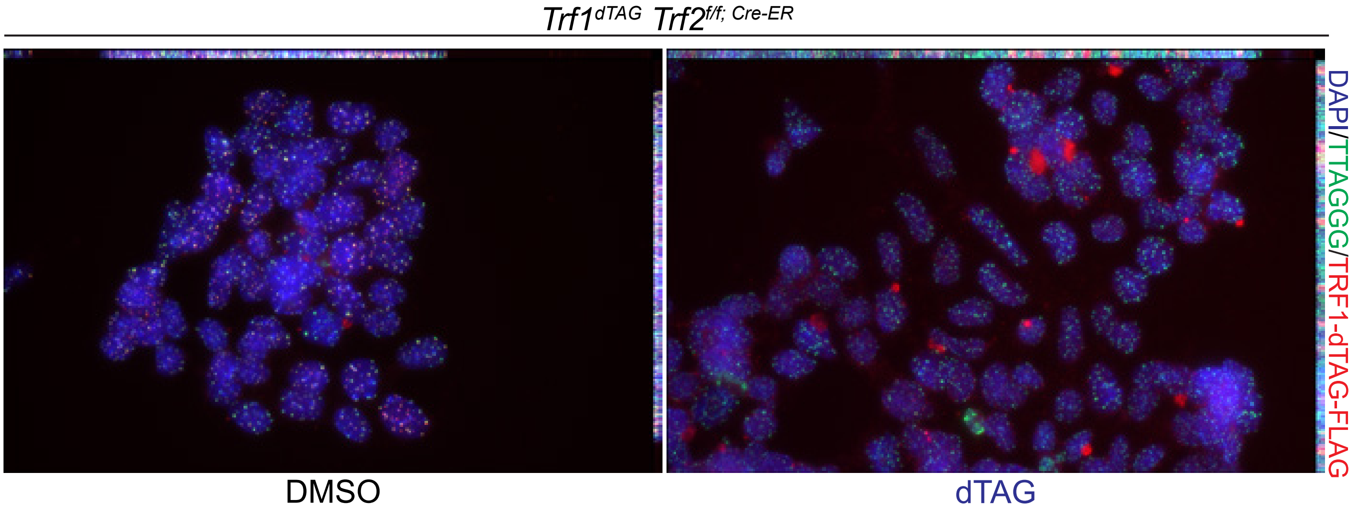

Extended Data Figure 5a Source Data

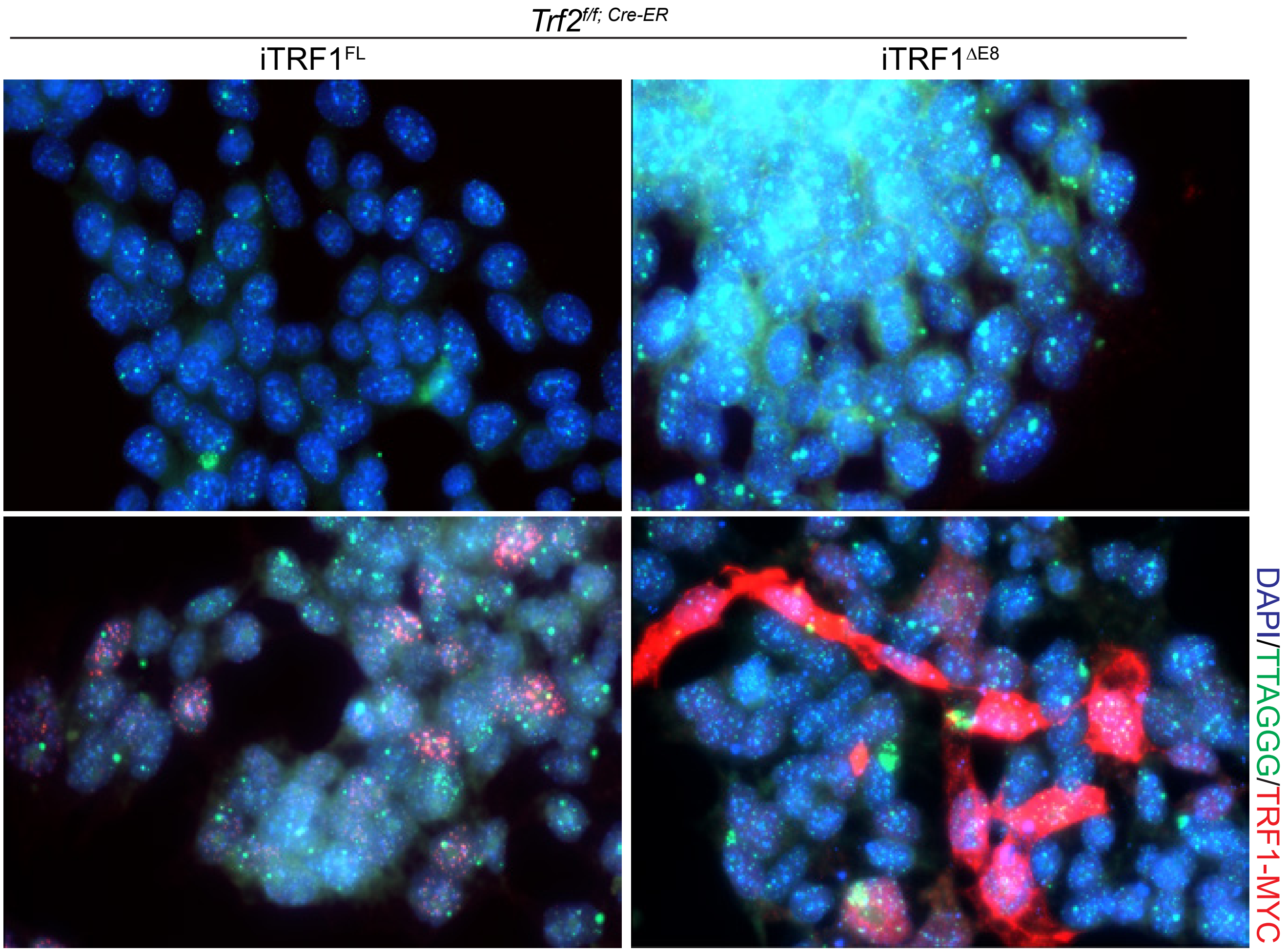

Extended Data Figure 5b Source Data

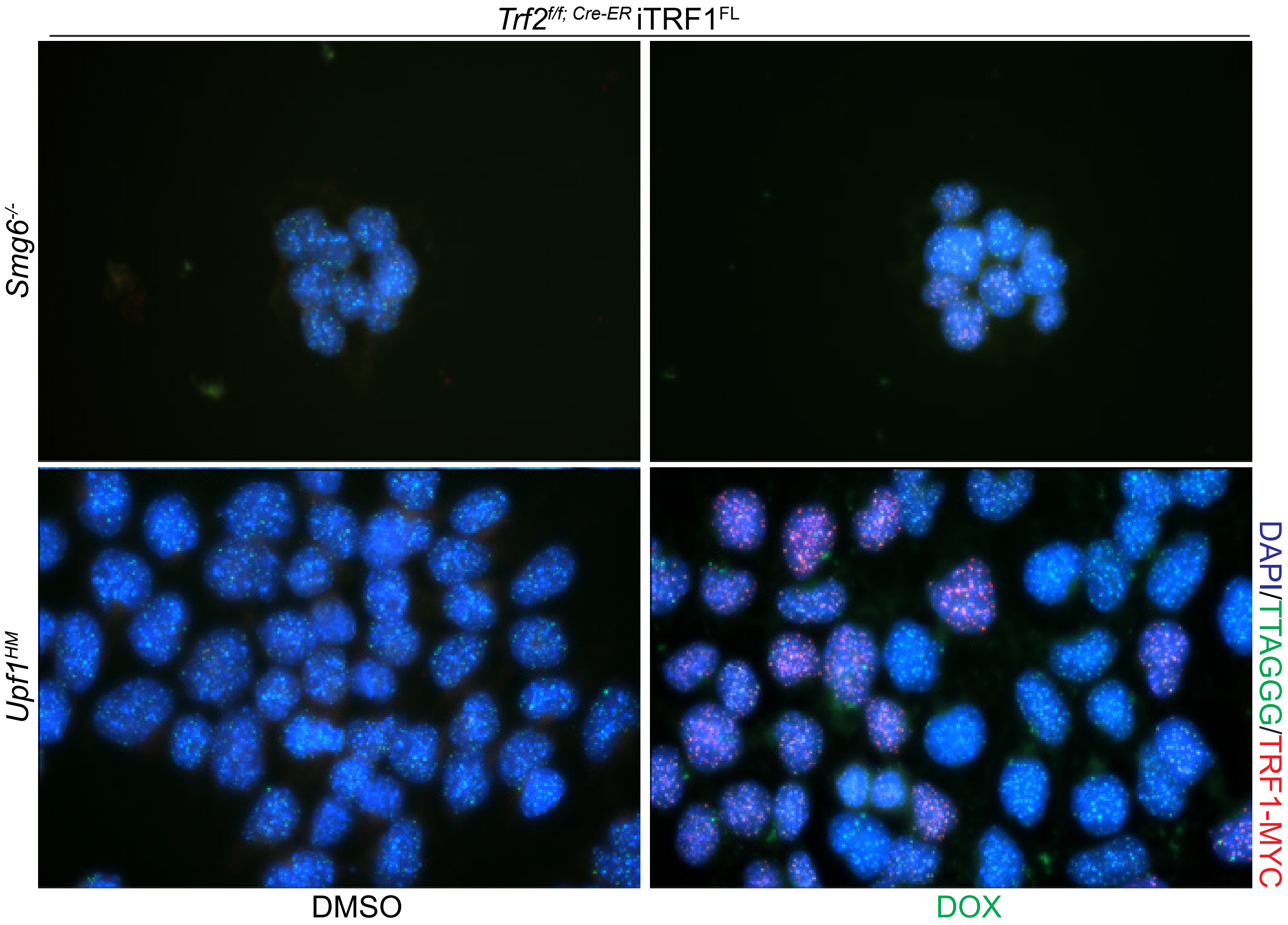

Extended Data Figure 5c Source Data

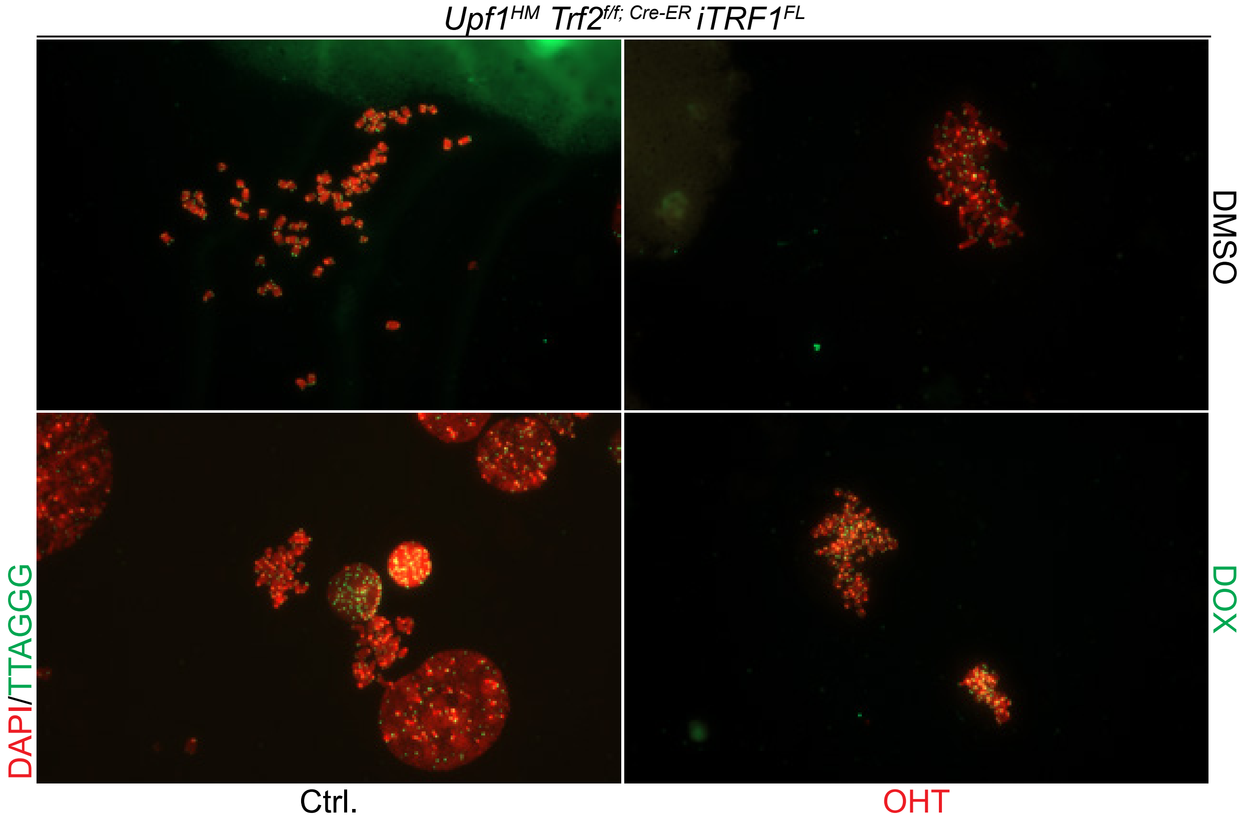

Extended Data Figure 5d Source Data

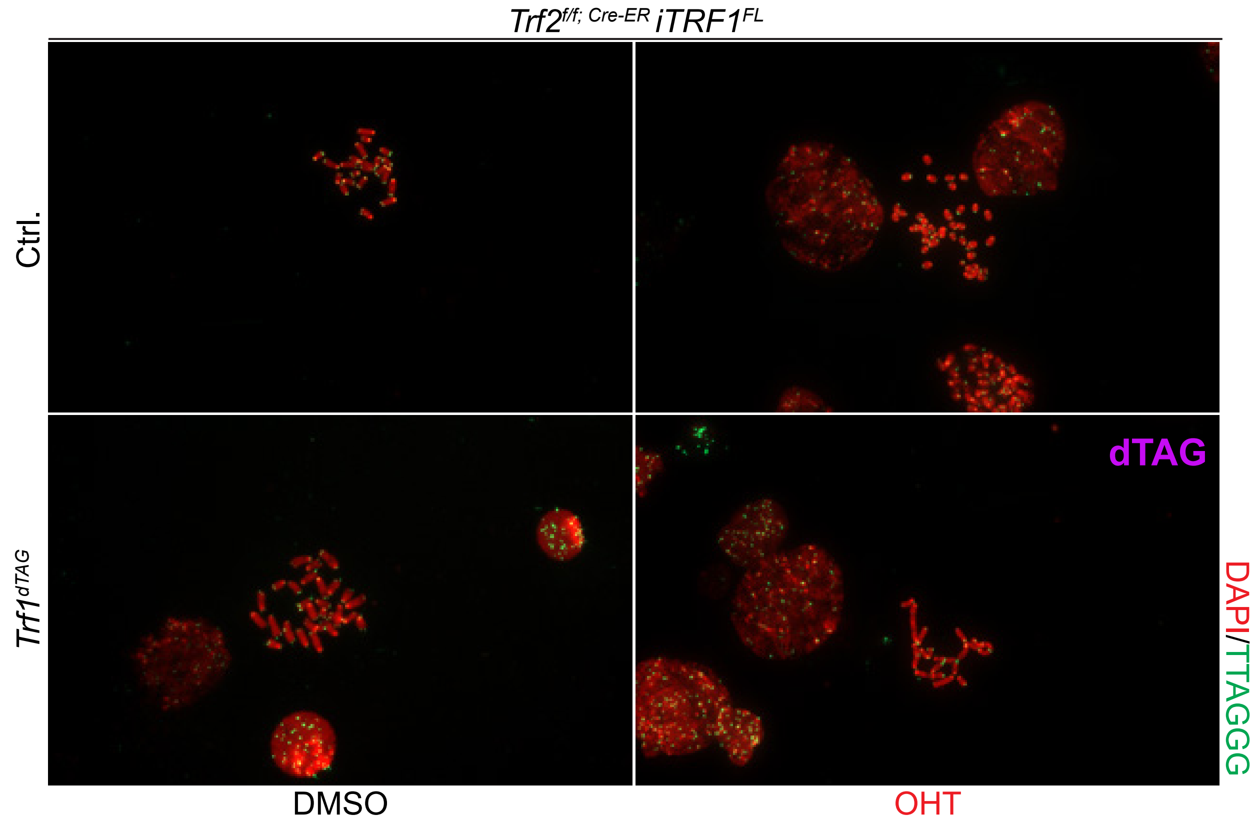

Extended Data Figure 5f Source Data

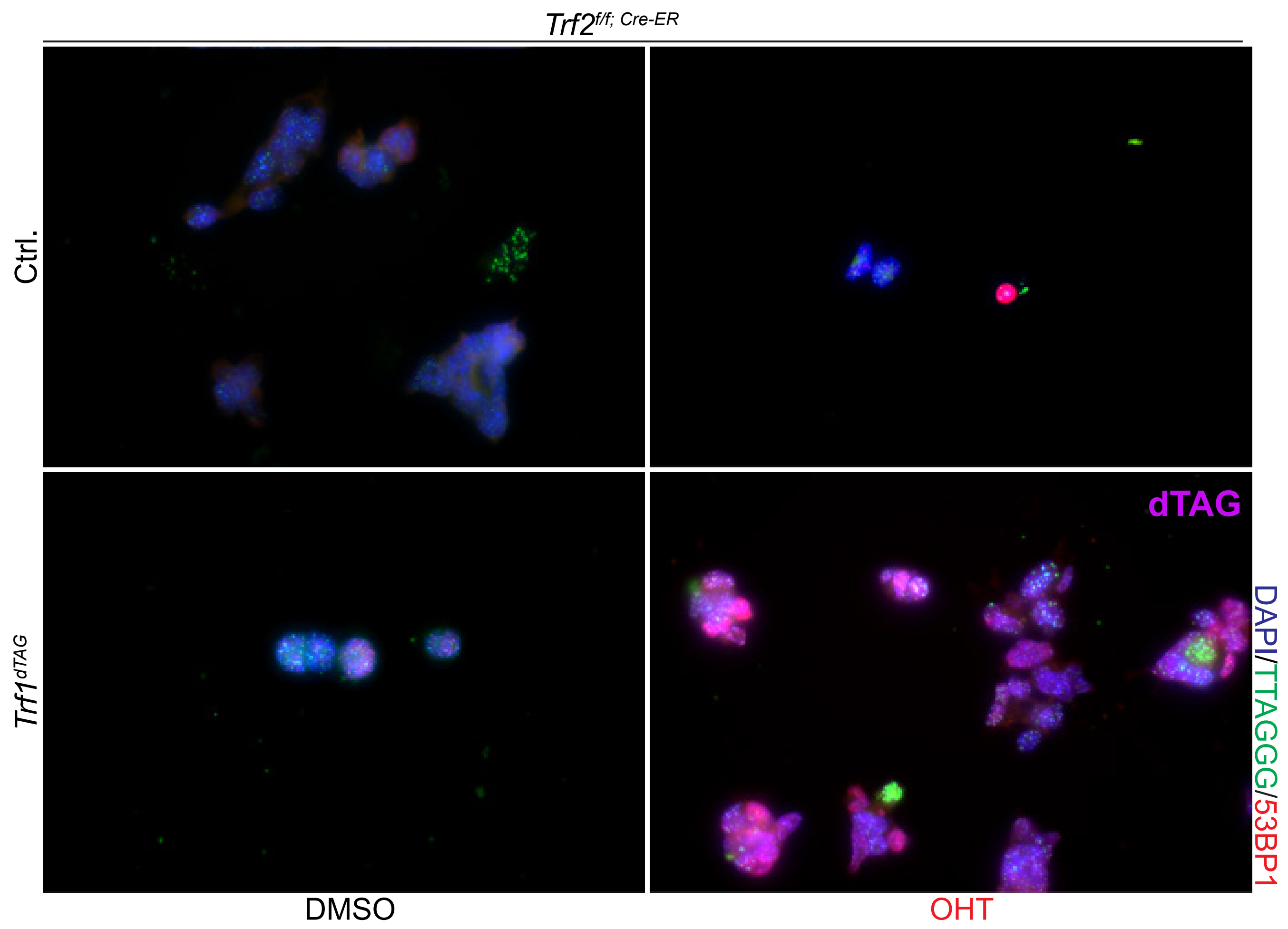

Extended Data Figure 5h Source Data

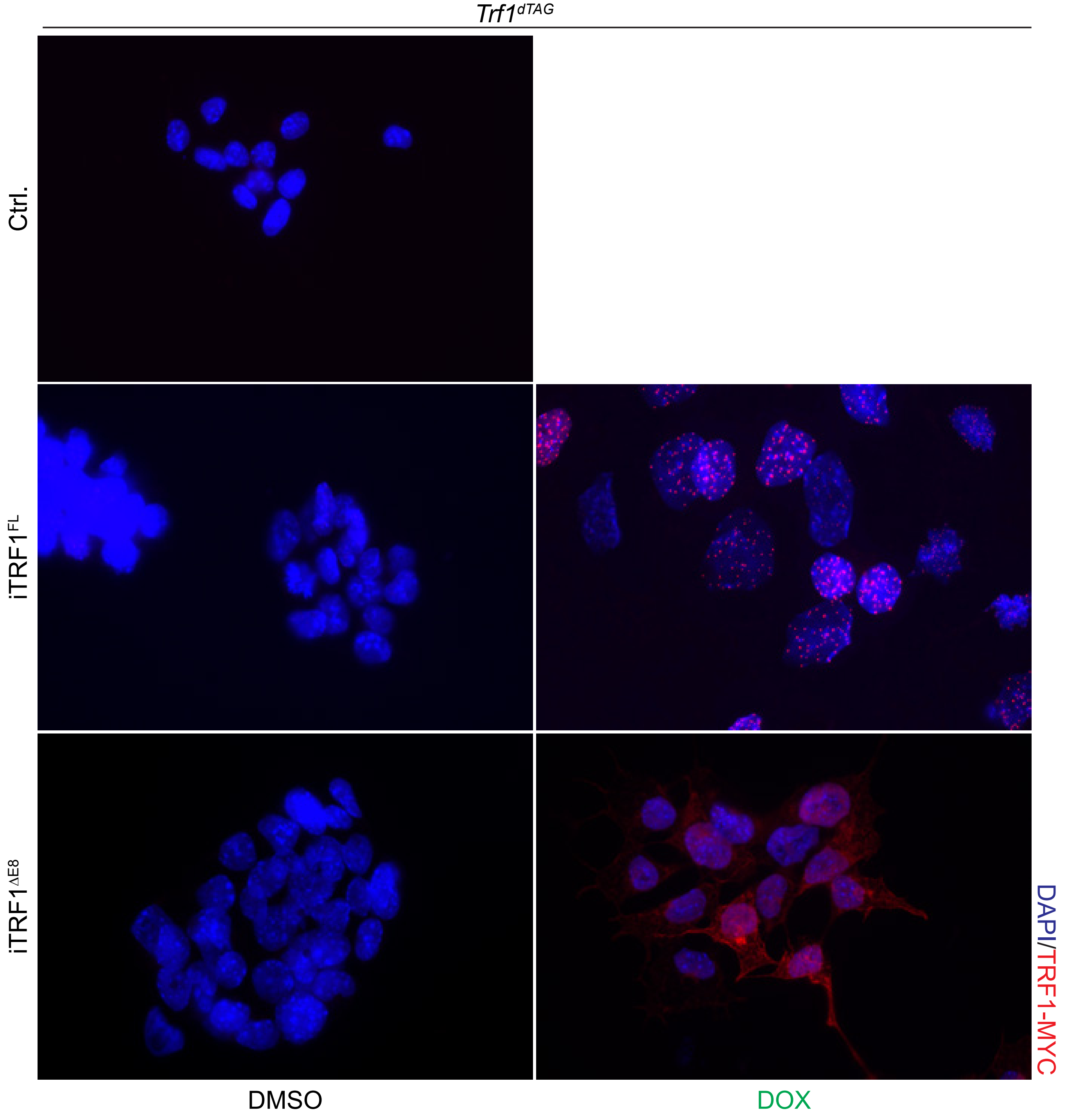

Extended Data Figure 5i Source Data

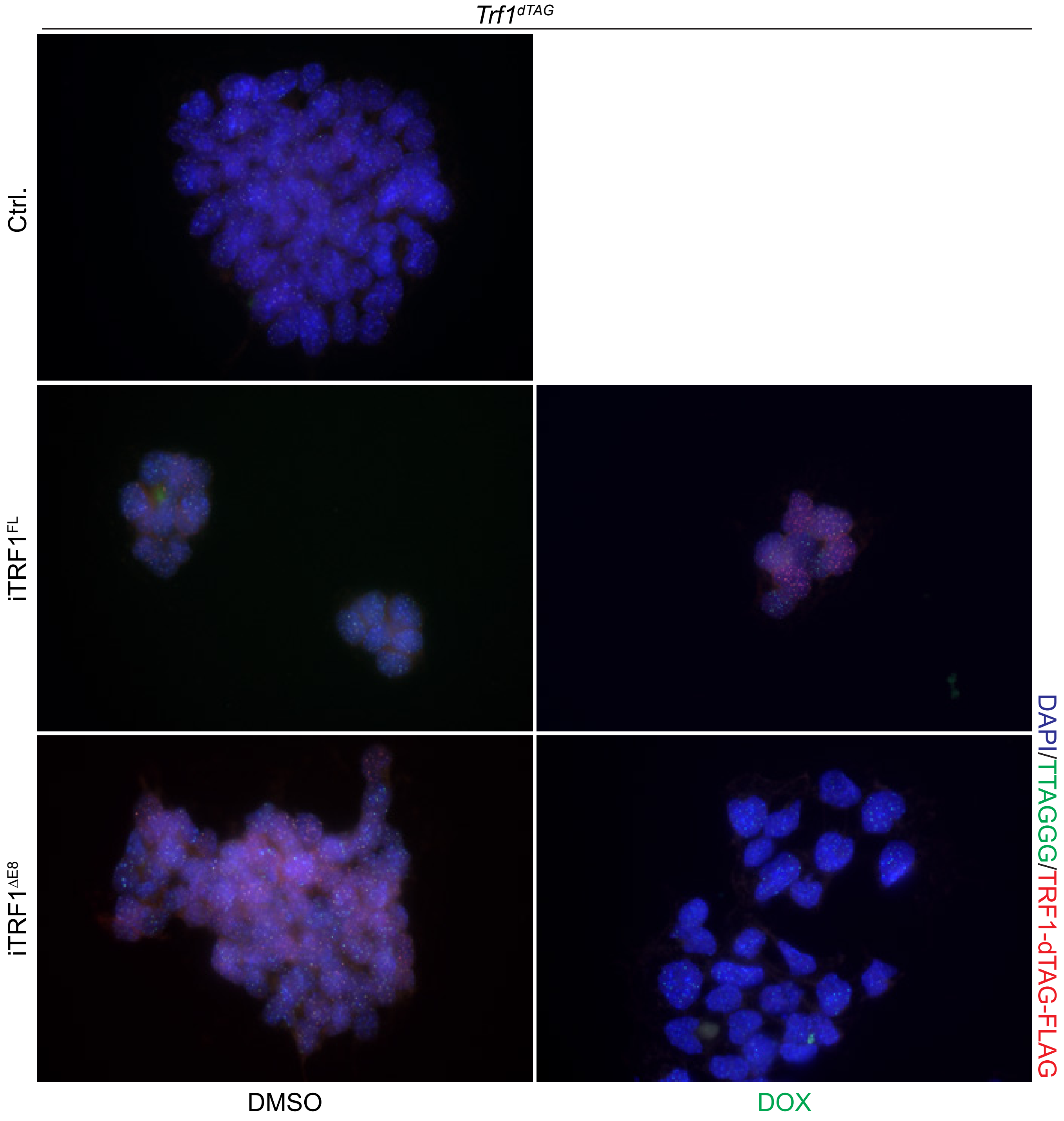

Supplement: Supplementary file 3 — Statistical source data. [file 41556_2026_1912_MOESM3_ESM.pdf]
